# Cancer-specific CTCF binding facilitates oncogenic transcriptional dysregulation

**DOI:** 10.1186/s13059-020-02152-7

**Published:** 2020-09-15

**Authors:** Celestia Fang, Zhenjia Wang, Cuijuan Han, Stephanie L. Safgren, Kathryn A. Helmin, Emmalee R. Adelman, Valentina Serafin, Giuseppe Basso, Kyle P. Eagen, Alexandre Gaspar-Maia, Maria E. Figueroa, Benjamin D. Singer, Aakrosh Ratan, Panagiotis Ntziachristos, Chongzhi Zang

**Affiliations:** 1 Department of Biochemistry and Molecular Genetics, Northwestern University, Chicago, IL USA; 2 Simpson Querrey Center for Epigenetics, Feinberg School of Medicine, Northwestern University, Chicago, IL USA; 3 Center for Public Health Genomics, University of Virginia School of Medicine, Charlottesville, VA USA; 4 Division of Experimental Pathology and Laboratory Medicine, Department of Laboratory Medicine and Pathology, Mayo Clinic, Rochester, MN USA; 5 Department of Medicine, Division of Pulmonary and Critical Care, Feinberg School of Medicine, Northwestern University, Chicago, IL USA; 6 Sylvester Comprehensive Cancer Center, Miller School of Medicine, University of Miami, Miami, FL USA; 7 Department of Human Genetics, Miller School of Medicine, University of Miami, Miami, FL USA; 8 Oncohematology Laboratory, Department of Women’s and Children’s Health, University of Padova, Padova, Italy; 9 Italian Institute for Genomic Medicine, 10060 Torino, Italy; 10 Department of Public Health Sciences, University of Virginia, Charlottesville, VA USA; 11 UVA Cancer Center, University of Virginia, Charlottesville, VA USA; 12 Robert H. Lurie Comprehensive Cancer Center, Northwestern University, Chicago, IL USA

**Keywords:** CTCF, 3D genome organization, Integrative analysis, Gene regulation, Transcription factor, Enhancer, T cell lymphoblastic leukemia, NOTCH1

## Abstract

**Background:**

The three-dimensional genome organization is critical for gene regulation and can malfunction in diseases like cancer. As a key regulator of genome organization, CCCTC-binding factor (CTCF) has been characterized as a DNA-binding protein with important functions in maintaining the topological structure of chromatin and inducing DNA looping. Among the prolific binding sites in the genome, several events with altered CTCF occupancy have been reported as associated with effects in physiology or disease. However, hitherto there is no comprehensive survey of genome-wide CTCF binding patterns across different human cancers.

**Results:**

To dissect functions of CTCF binding, we systematically analyze over 700 CTCF ChIP-seq profiles across human tissues and cancers and identify cancer-specific CTCF binding patterns in six cancer types. We show that cancer-specific lost and gained CTCF binding events are associated with altered chromatin interactions, partially with DNA methylation changes, and rarely with sequence mutations. While lost bindings primarily occur near gene promoters, most gained CTCF binding events exhibit enhancer activities and are induced by oncogenic transcription factors. We validate these findings in T cell acute lymphoblastic leukemia cell lines and patient samples and show that oncogenic NOTCH1 induces specific CTCF binding and they cooperatively activate expression of target genes, indicating transcriptional condensation phenomena.

**Conclusions:**

Specific CTCF binding events occur in human cancers. Cancer-specific CTCF binding can be induced by other transcription factors to regulate oncogenic gene expression. Our results substantiate CTCF binding alteration as a functional epigenomic signature of cancer.

## Background

The eukaryotic genome is known to fold into a hierarchical three-dimensional (3D) structure organized by numerous chromatin and transcription factor (TF) proteins [1]. High-throughput technologies such as Hi-C have helped delineate components of 3D genome organization, including topologically associating domains (TADs) [2–4] and DNA loops [5]. Studies have shown that various protein factors have roles in chromatin folding that is required for proper gene expression [3, 6–9]. One such factor is CCCTC-binding factor (CTCF), a DNA-binding protein that induces chromatin looping and binds at TAD boundaries [10]. CTCF is integral to cell survival as total knockouts in mice are lethal in early embryogenesis and heterozygous knockouts are predisposed to cancer [10–12]. Our previous studies using T cell acute lymphoblastic leukemia (T-ALL) models have shown that cell-type conserved constitutive CTCF binding sites frequently occur at chromatin domain boundaries and facilitate interactions between TF-bound distal enhancers and their target genes [13]. We demonstrated that TAD boundary intensity associates with CTCF levels, which might also serve to isolate super-enhancers [14]. While CTCF binding at TAD boundaries is usually conserved across diverse cell types and throughout development [15], we and others have shown that CTCF binding within TADs can also exhibit tissue specificity [14, 16–18].

The functional importance of CTCF is highlighted in disruptions to CTCF binding coupled with alterations in gene expression, which have been widely observed [19–22]. Deletions of insulator CTCF binding sites can cause aberrant chromatin interactions and differential expression of genes within TADs in developmental disorders and cancers [19, 20, 23–26]. Many cases of CTCF disruption have been associated with changes in DNA methylation such as in isocitrate dehydrogenase (IDH) mutant gliomas [21], succinate dehydrogenase (SDH)-deficient gastrointestinal stromal tumors (GIST) [22], and immunoglobulin or T cell receptors [18]. Additionally, the CTCF gene itself or its binding sequence can be mutated and has been suggested to be a haploinsufficient tumor suppressor [12]. CTCF mutations affecting the DNA-binding zinc finger domains compromise binding to the genome [27] and can occur in cancer [20, 28–30] or abnormal limb development [19]. Mispositioning of even one CTCF binding locus can trigger interactions leading to oncogene activation [20].

While specific CTCF binding sites have been shown to affect gene expression in development, physiology, and cancers [31–35], most others have seemingly little effect on chromatin interaction or gene expression [9, 36]. To date, there are few comprehensive analyses of global and cancer-specific CTCF binding patterns and their functional links to disease-related phenotypes. Here, we performed an integrative analysis of a large number of genomic profiles for CTCF as well as other TFs, chromatin marks, chromatin accessibility, gene expression, DNA methylation, somatic mutation, and in situ Hi-C to infer CTCF binding site functions. In six different cancer types, we identified cancer-specific gained and lost CTCF binding sites and showed that gain of CTCF binding in cancer associates with increased chromatin interactions and cancer-specific gene activation, while loss of CTCF binding occurred at promoters of genes present with lower expression in cancer compared to normal cells. We validated our findings in T-ALL and found that gained CTCF binding sites are potentially incurred by the activity of oncogenic TFs such as NOTCH1. These findings show that cancers exhibit an oncogenic CTCF binding signature that is intimately tied to chromatin topology and dysregulated gene expression. The putative causative link to oncogenic transcriptional program suggests that altered CTCF binding is an important component of the mechanism of cancer pathogenesis.

## Results

### Cancers exhibit unique CTCF binding patterns in the genome

**Fig. 1:**
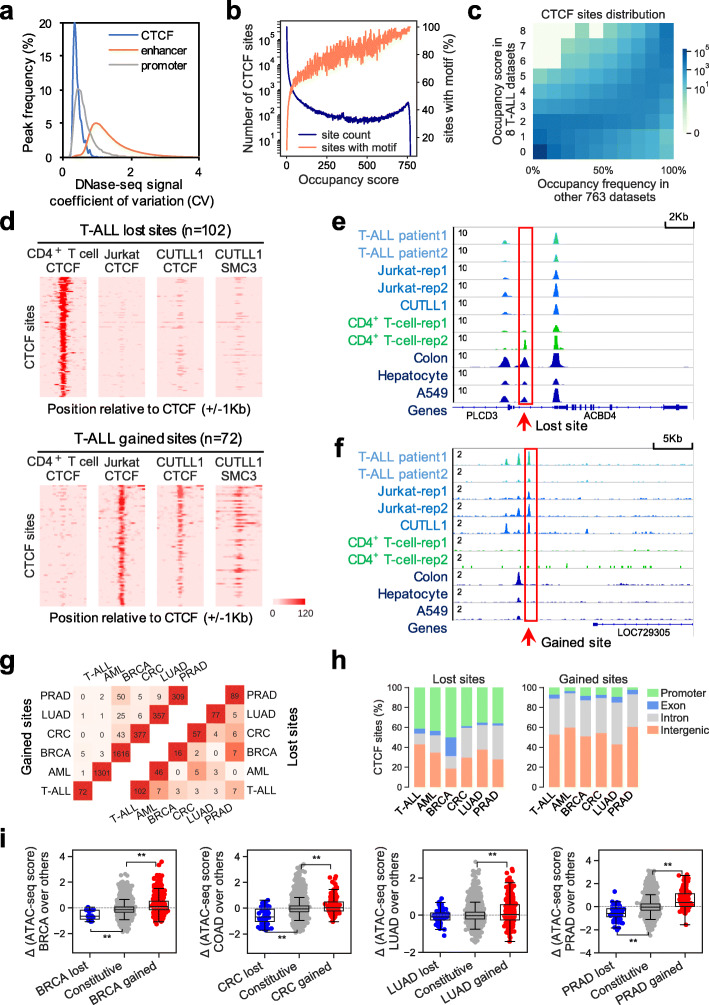
Identification of cancer-specific CTCF binding patterns in the human genome. **a** Distribution of coefficient of variation of chromatin accessibility at different genomic features, calculated using DNase-seq data from over 60 cell lines from ENCODE. **b** Distribution of occupancy score for all 688,429 union CTCF binding sites (blue), and percentage of CTCF sites that contain a CTCF motif at each occupancy score (orange). **c** Distribution of CTCF binding occupancy score in 8 ChIP-seq datasets for T-ALL cell lines (*y*-axis) and the occupancy frequency score in the other 763 ChIP-seq datasets (*x*-axis). Color density in each unit represents the number of CTCF binding sites with designated scores. **d** CTCF ChIP-seq signals at a 2-kb region surrounding T-ALL_lost_ (top) and T-ALL_gained_ (bottom) binding sites in normal CD4+ T cells and the T-ALL cell lines Jurkat and CUTLL1, and SMC3 signals at the same regions in CUTLL1. **e** Example of CTCF ChIP-seq signals around a T-ALL-specific lost CTCF binding site. **f** Example of CTCF ChIP-seq signals around a T-ALL-specific gained CTCF binding site. **g** Number of identified gained (left) and lost (right) CTCF binding sites in each of the 6 cancer types and number of shared sites between each pair of cancer types. Color density of each element represents the level of similarity measured by Jaccard index. **h** Genomic distribution of identified lost (left) and gained (right) CTCF binding sites in the 6 cancer types. Promoter regions are defined as ± 2 kb from any TSS in the genome. **i** Differential chromatin accessibility (ATAC-seq) in TCGA patient samples at identified cancer-specific lost (blue), gained (red), and constitutive (gray) CTCF binding sites in each of the 4 cancer types compared to all other TCGA samples. *, *p* < 0.05, **, *p* < 0.001, by two-tailed unpaired Student’s *t* test

CTCF binding sites are among the most stable regulatory elements in the human genome across cell types, compared to gene promoters and distal enhancers (Fig. 1a). To comprehensively study the genomic repertoire of CTCF binding, we collected 771 high-quality CTCF ChIP-seq datasets from the public domain. These datasets cover over 200 human cell types, including normal tissues and multiple cancer types (Additional file 1: Fig. S1a, Additional file 2: Table S1). We collectively identified 688,429 distinct CTCF binding sites by merging shared peaks called from each dataset (Additional file 1: Fig. S1b,c). To study the binding occupancy pattern across cell types, we assigned an occupancy score to each CTCF site by tallying the ChIP-seq datasets exhibiting peaks within the site (Additional file 1: Fig. S1b). We obtained a broad spectrum of CTCF binding site distribution from sample-specific to cross-sample conserved (constitutive) (Fig. 1b) and focused on the 285,467 high-confidence CTCF binding sites with occupancy score ≥ 3. We identified 22,097 constitutive CTCF binding sites, which were defined as binding present in at least 80% of all 771 datasets determined by an empirical model (Fig. 1b, Additional file 1: Fig. S1d).

To identify cancer-specific CTCF binding patterns, we surveyed six cancer types: T cell acute lymphoblastic leukemia (T-ALL), acute myeloid leukemia (AML), breast cancer (BRCA), colorectal cancer (CRC), lung cancer (LUAD), and prostate cancer (PRAD). These cancers have CTCF ChIP-seq data available in both cell lines and corresponding normal tissues (Additional file 3: Table S2). We compared both CTCF occupancy frequencies and normalized CTCF binding levels in samples from each cancer type versus all other samples (Fig. 1c, Additional file 1: Fig. S1e-i) as well as their corresponding normal tissue (Additional file 1: Fig. S1j-p) to account for variations due to sample specificity. We categorized a CTCF binding event as lost in a cancer if it had a lower occupancy score and lower binding level in the cancer cell lines compared to the corresponding normal tissue and to all other samples. A site was characterized as gained in a cancer if it had a higher occupancy score and higher binding level in the cancer cell lines compared to the corresponding normal tissue as well as to all other samples (see detailed statistical assessment in “Methods”). Using this approach, we identified lost and gained CTCF binding sites in each of the six cancer types (Additional file 4: Table S3) and confirmed that most identified lost/gained sites have significantly reduced/elevated CTCF binding levels (FDR ≤ 0.05, fold change ≥ 2) in that cancer compared to all other samples (Additional file 1: Fig. S2a), with absolute effect sizes ranging between 0.93 and 1.87 and an average of 1.46 (Additional file 1: Fig. S2b,c). As an example, the CTCF binding patterns at 102 lost and 72 gained sites identified in T-ALL are shown in Fig. 1d–f, comparing normal CD4^+^ T cell with two T-ALL cell lines, Jurkat and CUTLL1 [34, 35, 37–40]. SMC3, component of the cohesin complex, exhibits weak/substantial signals at lost/gained CTCF sites in CUTLL1, respectively, indicating the CTCF binding alteration is a functional event (Fig. 1d).

Comparing specific lost/gained CTCF sites from the six cancer types, we observed that different cancer types share few commonly gained sites (Jaccard index < 0.03), much less than shared all CTCF sites between each other (Jaccard index between 0.33 and 0.71), indicating cancer-type specificity of the identified CTCF sites (Fig. 1g, Additional file 1: Fig. S2d, Additional file 5: Table S4). Interestingly, although commonly lost sites comparing each pair of cancer types are also few, 7 cases out of 15 pair-wise comparisons do show a significant commonality of CTCF binding loss under the background of all constitutively bound sites (Additional file 5: Table S4, *P* < 0.01, by Fisher’s exact test). It is worth noting that the large difference across cancer types on the number of lost/gained CTCF sites identified under the same statistical criteria is possibly due to the various number of available samples for different cancer types (Additional file 1: Fig. S1e-o) and the wide range of CTCF peak numbers across samples (Additional file 1: Fig. S1a). Across cancer types, most altered sites contain CTCF motifs in the binding sequences (Additional file 1: Fig. S3a), consistent with the motif occurrence association with the global occupancy distribution (Fig. 1b). Lost sites are enriched at gene promoter regions (< 2 kb from any transcription start site, TSS), while gained sites are primarily located at distal and non-coding regions (Fig. 1h), regardless of CTCF motif presence or absence within the binding sites (Additional file 1: Fig. S3b). This suggests that different cancers may employ similar mechanisms leading to CTCF binding loss or gain.

We further explored these lost and gained CTCF binding events identified from cancer cell lines in patient samples, to confirm that these unique patterns are not cell line-specific phenomena. In two T-ALL patient samples, 78 of the 102 lost sites (T-ALL_lost_) were also depleted in at least one sample, and 33 of the 72 gained sites (T-ALL_gained_) are present in at least one sample (Additional file 1: Fig. S3c). As CTCF binding positively correlates with chromatin accessibility [41] (Additional file 1: Fig. S3d, Additional file 6: Table S5), we systematically surveyed ATAC-seq data in BRCA, COAD, LUAD, and PRAD patient samples from The Cancer Genome Atlas (TCGA) [42] and consistently observed that lost or gained CTCF sites identified from cell lines exhibit lower or higher chromatin accessibility, respectively, compared to the entire TCGA cohort (Fig. 1i)(*P* < 0.001 by *t*-test, except LUAD_lost_), regardless of CTCF motif occurrence (Additional file 1: Fig. S3e,f), indicating that unique losses or gains in CTCF binding exist extensively in cancer patients.

### Unique CTCF binding sites link to patterns of chromatin interaction

**Fig. 2:**
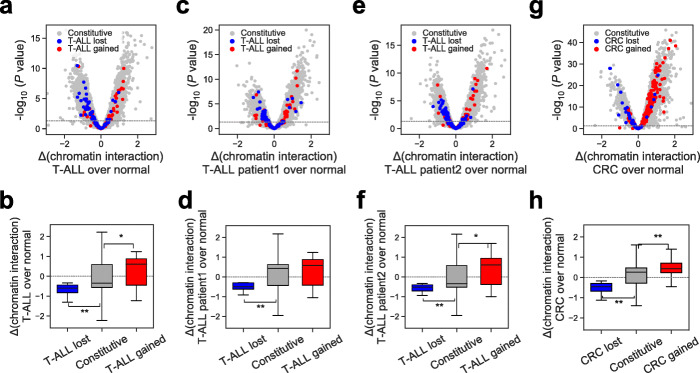
Gained/lost CTCF binding events associate with chromatin dynamics. **a**, **c**, **e**, **g** Volcano plots showing differential chromatin interaction levels between cancer and normal cells at cancer-specific lost (blue), gained (red), and constitutive (gray) CTCF binding sites, measured by Hi-C. Each point represents the interaction changes between a CTCF binding site and 5-kb bins located within 500 kb from the site. Horizontal dotted line represents *p* value cutoff of 0.05, by two-tailed paired Student’s *t* test. **b**, **d**, **f**, **h** Boxplots showing differential interaction frequencies between cancer and normal matched tissues for each group of CTCF binding sites. *, *p* < 0.05, **, *p* < 0.001, by two-tailed unpaired Student’s *t* test

As CTCF was known to induce DNA looping and is enriched at TAD boundaries [1], we then interrogated the relationship between altered CTCF occupancy and chromatin conformation in cancer. We performed in situ Hi-C in Jurkat, healthy donor CD4^+^ T cells, and patient T-ALL cells [5, 43, 44]. Differential analysis of our Hi-C data revealed that T-ALL_lost_ sites and T-ALL_gained_ sites have decreased or increased contact frequencies with their flanking regions, respectively, in T-ALL compared to normal T cells, using constitutive CTCF sites as controls (Fig. 2a,b, Additional file 1: Fig. S4a) (*P* < 0.05 by *t*-test). These findings were corroborated in our two T-ALL patient samples (Fig. 2c–f, Additional file 1: Fig. S4b,c) and in other malignancies such as CRC (*P* < 0.001 by *t*-test) (Fig. 2g,h, Additional file 1: Fig. S4d, Additional file 6: Table S5), regardless of presence or absence of CTCF motifs (Additional file 1: Fig. S5). Together, these results suggest that cancer-specific alterations to CTCF binding highly associate with changes in local chromatin contacts relative to their normal physiological state.

In addition to regulating chromatin conformation, CTCF occupancy was suggested to act as a boundary against spreading of histone modifications between loops and TADs [2, 5]. Therefore, we assessed whether cancer-specific CTCF binding is implicated in histone modification patterns. Using publicly available ChIP-seq data, we examined the activating histone marks H3K4me1, H3K27ac, and the repressive mark H3K27me3 and found that cancer-specific gained CTCF binding associates with increased levels of enhancer marks H3K4me1 and H3K27ac (*P* < 0.001 by *t*-test), while lost CTCF sites correlate with decreased level of these two enhancer marks (*P* < 0.05 by *t*-test) (Additional file 1: Fig. S6). Decreased H3K4me1 and H3K27ac at lost sites were also observed in T-ALL patient samples (*P* < 0.001 by *t*-test) (Additional file 1: Fig. S7). We did not observe a consistent trend of change in the H3K27me3 level at either gained or lost CTCF sites.

### Cancer-specific CTCF binding gain and loss associate with differential gene expression within chromatin domains

**Fig. 3:**
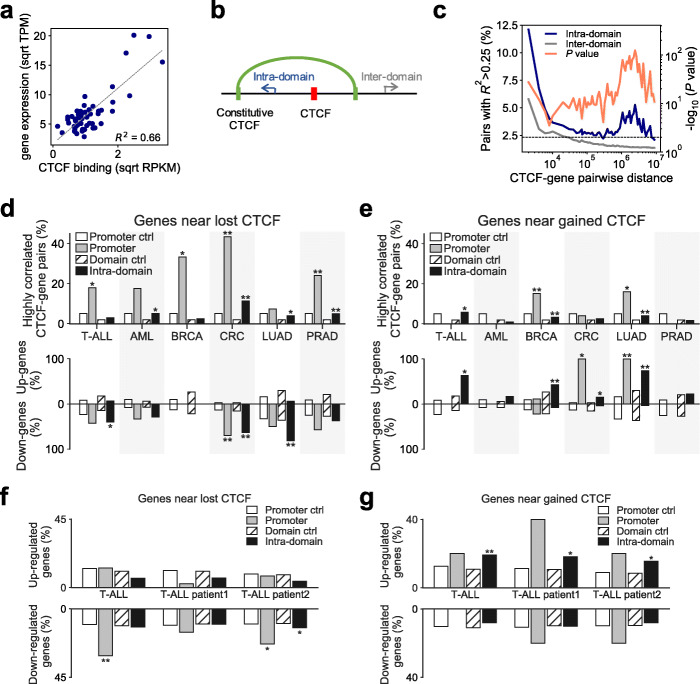
Gained/lost CTCF binding events associate with differential gene expression in cancer. **a** CTCF ChIP-seq signals (*x*-axis) and gene expression levels (*y*-axis) for one CTCF site-gene pair in 54 cell types with matched data available. *R*^2^ is calculated as the association score. Sqrt, Square root; TPM, transcript count per million reads; RPKM, read count per kilobases per million reads. **b** Schematic of categories of intra-chromatin-domain and inter-chromatin-domain gene-CTCF pairs. **c** Distribution of highly correlated CTCF-gene pairs (defined as *R*^2^ > 0.25) as a function of the distance between the CTCF binding site and the gene’s TSS. Pairs located within the same CTCF domain (intra-domain, blue) and across different CTCF domains (inter-domain, gray) are plotted separately. *P* values were obtained using two-tailed Fisher’s exact test. Dashed line represents *P* = 0.01. **d**, **e** Top: Percentage of highly correlated CTCF-gene pairs in which the gene sits within the same domain as a cancer-specific lost (**d**) or gained (**e**) binding site, with constitutive sites as control. “Promoter” refers to genes whose promoter region (TSS ± 2 kb) contains a CTCF binding site from a certain category. “Promoter ctrl” refers to genes whose promoter region contains a constitutive CTCF binding site as the control for cancer-specific gained/lost sites. “Intra-domain” refers to genes whose chromatin domain contains a CTCF binding site. “Domain ctrl” refers to genes whose chromatin domain contains a constitutive CTCF site as the control for those with cancer-specific gained/lost sites. Bottom: Percentage of differentially expressed genes (|log2FC| > 1, FDR < 1e−5) contained within the corresponding group of either Promoter or Intra-domain highly correlated CTCF-gene pairs in the corresponding cancer type. *, *p* < 0.05, **, *p* < 0.001, by two-tailed Fisher’s exact test. **f**, **g** Percentage of genes that are upregulated (top, log2FC > 1, FDR < 1e−5) or downregulated (bottom, log2FC < − 1, FDR < 1e−5) located in the chromatin domains containing certain group of lost (**f**) or gained (**g**) CTCF sites. *, *p* < 0.05, **, *p* < 0.001, by two-tailed Fisher’s exact test

To study the effects of CTCF binding on gene expression, we used an unbiased approach and compiled a comprehensive list of all possible combinations of CTCF site and gene pairs that are located within the same chromosome [4, 12, 13]. We measured both CTCF binding level and gene expression level for each CTCF-gene pair and calculated their correlation across 54 cell types for which both CTCF ChIP-seq and RNA-seq data are available (Additional file 6: Table S5). The coefficient of determination (*R*^2^) value can represent the association between CTCF binding and gene expression (Fig. 3a). Upon dividing the CTCF-gene pairs into two groups based on whether the paired loci are in the same or different divergently oriented constitutive CTCF-bound chromatin domains [13] (Fig. 3b, hereafter referred to as “chromatin domains”), we found that pairs in the same domain are more likely to be highly correlated (*R*^2^ > 0.25, Additional file 1: Fig. S8a), regardless of genomic distance (Fig. 3c). This indicates that any effect of CTCF binding in regulating gene expression tends to be confined within chromatin domains [13]. These domains are highly consistent with TADs identified from Hi-C maps [45] (Additional file 1: Fig. S8b,c).

We then tested whether those CTCF binding sites specifically lost or gained in cancer associate with expression of genes within the same chromatin domains. If a CTCF binding site is located in a gene promoter region, we directly used that gene as the promoter candidate target. Otherwise, we assigned all genes located within the same domain as the CTCF site as intra-domain candidate target genes. Using this rubric, we found that cancer-specific lost CTCF binding events tend to have higher correlation (*R*^2^ > 0.25) with their promoter target genes (Fig. 3d top, gray bars), which are also more likely to be downregulated in cancer (Fig. 3d bottom, gray bars). Genes that strongly associate (*R*^2^ > 0.25) with cancer-specific gained CTCF binding sites, on the other hand, tend to be upregulated in cancer (Fig. 3e, black bars). In general, even without considering CTCF-gene pair correlations, genes surrounding lost CTCF binding sites within the same chromatin domain tend to be down regulated (Additional file 1: Fig. S8d), while genes surrounding gained CTCF binding sites are more likely to be activated (Additional file 1: Fig. S8e). This relationship also holds in at least one of the two T-ALL patient samples (Fig. 3f,g). Furthermore, we found that genes highly correlated with intra-domain BRCA_gained_ CTCF sites are enriched for the essential genes identified using CRISPR-screen data from the breast cancer cell line T47D [46], suggesting that gained CTCF is involved in cancer functions (Additional file 1: Fig. S8f).

### Cancer-specific CTCF binding patterns associate partially with differential DNA methylation and rarely with sequence mutations

**Fig. 4:**
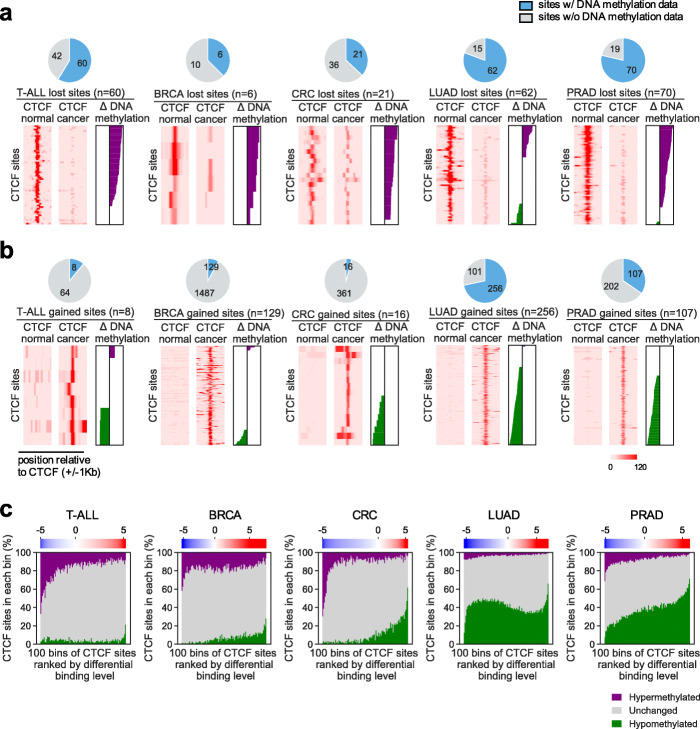
Patterns of differential DNA methylation near cancer-specific lost/gained CTCF sites. **a**,**b** ChIP-seq signals and differential DNA methylation levels surrounding specific lost (**a**) or gained (**b**) CTCF binding sites in cancer versus normal tissues for each of the 5 cancer types. Pie charts represent numbers of CTCF sites with (light blue) or without (gray) sufficient DNA methylation signals from bisulfite sequencing data. For sites with sufficient DNA methylation signals, heatmaps show CTCF ChIP-seq signals cover 2-kb regions centered at each site. Differential DNA methylation level at a 300 bp region centered at each CTCF binding site was calculated and presented as a waterfall plot. Purple bars represent increased and green bars represent decreased DNA methylation levels (with values in a range from 0 to 100). Rows in corresponding ChIP-seq and DNA methylation plots are ranked identically. **c** Association between CTCF binding specificity and differential DNA methylation for each cancer type. All CTCF sites with sufficient DNA methylation coverage in each cancer type were ranked based on their differential CTCF binding level in cancer compared to other samples and grouped into 100 equal-count bins. For each bin, the percentages of sites associated with DNA hypomethylation (green), unchanged methylation (gray), and hypermethylation (purple) were stacked in a column. Top color bar represents the median differential binding level of the CTCF sites in a bin, quantified as the *T*-test statistic

We next sought to identify determinants of cancer-specific CTCF binding. To date, the primary identified effectors of variation in CTCF binding at specific loci in cancers include altered DNA methylation at a CTCF motif [20, 21, 30, 47] or mutations affecting the CTCF binding sequence [30]. Prior studies have shown that CTCF binding negatively correlates with DNA methylation [15, 48]. We collected reduced representation bisulfite sequencing (RRBS) data from T-ALL, BRCA, and CRC and whole-genome bisulfite sequencing (WGBS) data from LUAD and PRAD for their DNA methylation profiles in cancer cells and corresponding normal tissues, and focused on the subsets of lost or gained CTCF binding regions that have sufficient bisulfite sequencing reads to call DNA methylation levels (Fig. 4a,b). We calculated the differential levels of DNA methylation over a 300-bp region centered at each CTCF binding site. We noticed that the majority of lost CTCF binding sites are associated with increased DNA methylation (Fig. 4a) and many gained CTCF sites are associated with DNA methylation reduction (Fig. 4b), consistent with existing knowledge. Meanwhile, we also observed that in almost every cancer type, several lost CTCF sites (up to 68% for LUAD) do not associate with DNA methylation increase for at least 20%, and many (22%–85%) gained sites do not show at least 20% of DNA methylation decrease, regardless of whether the data are RRBS or WGBS. Such patterns are also consistently observed at both motif-present CTCF sites (Additional file 1: Fig. S9a) and motif-absent CTCF sites (Additional file 1: Fig. S9b). We then examined the genome-wide association between CTCF binding specificity and differential DNA methylation in each cancer type and found a consistent correlation across the genome (Fig. 4c). We concluded that a considerable portion of cancer-specific CTCF binding alteration events associate with DNA methylation change in the binding regions.

Stable CTCF binding is highly specific to the presence of its DNA-binding motif and can be compromised by mutations affecting the consensus motif sequence [20, 30]. We performed whole genome sequencing (WGS) in T-ALL samples with an average sequencing depth of ~ 37× (Additional file 1: Fig. S10a), and found very few genetic alterations at gained or lost binding loci that can change the CTCF motif (Additional file 1: Fig. S10b). Using WGS data for AML, BRCA, COAD, LUAD, and PRAD patient samples from the International Cancer Genome Consortium (ICGC) [49], we consistently observed that few CTCF loss or gain associates with mutations altering the consensus binding sequence (Additional file 1: Fig. S11). Compositing all mutations in each cancer type around CTCF binding sites, we did not observe an enrichment of mutation rate at the center of the cancer-specific lost/gained sites relative to the flanking 400-bp regions (Additional file 1: Fig. S12a-d). Although it was shown that integrated pan-cancer mutations from ICGC exhibit an enrichment at constitutive CTCF sites [20] (Additional file 1: Fig. S12e), such enriched mutation rate pattern was not observed at lost/gained CTCF sites from any cancer type (Additional file 1: Fig. S12f,g). These data show that cancer-specific CTCF binding events can rarely be attributed to DNA sequence mutations.

### Cancer-specific gained CTCF co-activates target genes with oncogenic transcription factors

**Fig. 5:**
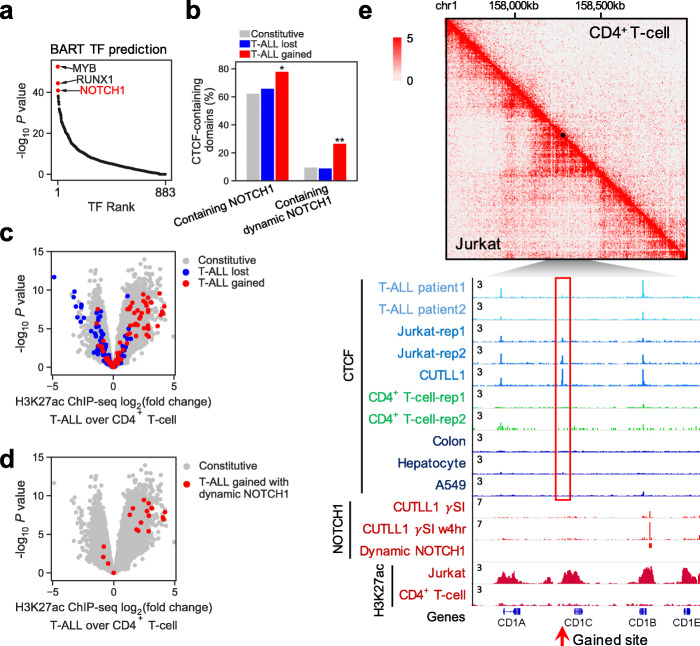
T-ALL_gained_ CTCF binding associates with oncogenic NOTCH1 binding and increased chromatin interaction. **a** BART-predicted transcription factors binding in genomic regions that have increased interaction with T-ALL_gained_ CTCF sites comparing Jurkat cells with normal CD4^+^ T cells. **b** Percentage of chromatin domains including different groups of CTCF binding that contain a NOTCH1 binding site or a dynamic NOTCH1 binding site. *, *p* < 0.05, **, *p* < 0.001, by two-tailed Fisher’s exact test. **c**, **d** T-ALL_gained_ sites associate with an increased H3K27ac level in Jurkat cells. **c** Volcano plot showing the differential H3K27ac level between Jurkat cells and normal CD4^+^ T cells measured by ChIP-seq; each point represents a 10-kb region surrounding a CTCF binding site. **d** Regions containing dynamic NOTCH1 binding sites are highlighted in red. **e** Example of Hi-C interaction maps and ChIP-seq tracks around a T-ALL_gained_ CTCF binding site

CTCF has been shown to co-bind DNA with other factors to establish DNA loops and control gene expression [19, 50]; thus, we looked for TFs potentially involved in cancer-specific CTCF gain events that associate with dynamic chromatin interaction and increased gene expression. Direct DNA sequence motif search in the lost/gained sites did not yield any motifs unambiguously enriched other than CTCF itself (Additional file 1: Fig. S13, Additional file 7: Table S6). Therefore, we sought to compare our in situ Hi-C [4, 5, 14, 51, 52] data in T-ALL with normal CD4^+^ T cells to identify genomic regions within the same chromatin domain that interact more frequently with T-ALL_gained_ CTCF sites (Additional file 1: Fig. S14a,b), and used *BART* [53] to identify putative transcriptional regulators that preferentially bind in these regions. BART analysis predicted MYB, RUNX1, and NOTCH1 as the top 3 ranked TFs with binding sites enriched in these regions (Fig. 5a, Additional file 8: Table S7). MYB has been shown as a key factor in T-ALL with super-enhancer functions together with TAL1 and RUNX1 [54, 55], while NOTCH1 is known to be a major oncogenic driver in T-ALL that also has super-enhancer functions [13]. Potential oncogenic TFs in CRC were also identified using the same approach (Additional file 1: Fig. S14c, Additional file 8: Table S7). Indeed, compared to normal CD4^+^ T cells, gained CTCF sites in T-ALL interact more frequently with “dynamic” NOTCH1 binding sites, previously defined as those sensitive to gamma-secretase inhibitor (γSI) treatment followed by inhibitor washout [13] (Additional file 1: Fig. S14d). Furthermore, beyond the identified T-ALL_gained_ CTCF sites, we found a genome-wide positive correlation between CTCF binding specificity in T-ALL and co-occurrence of NOTCH1 binding within the chromatin domain, using all CTCF sites in T-ALL as a background (Additional file 1: Fig. S14e). Indeed, both NOTCH1 (odds ratio = 2.1; *P* = 7e−3) and dynamic NOTCH1 sites (odds ratio = 3.4; *P* = 3.6e−5) are significantly enriched in chromatin domains containing T-ALL_gained_ CTCF sites (Fig. 5b, Additional file 1: Fig. S14f), although NOTCH1 and CTCF do not co-occupy the same loci (Additional file 1: Fig. S14g). Specifically, 56 (78%) out of 72 T-ALL_gained_ CTCF sites share the chromatin domain with at least a NOTCH1 site, and 19 (26%) share a domain with at least a dynamic NOTCH1 site (Fig. 5b, Additional file 1: Fig. S14f). These T-ALL_gained_ CTCF sites are also associated with increased levels of H3K27ac in T-ALL (*P* = 7.8e−47 by *t*-test), indicative of potential enhancer function (Fig. 5c,d). An example locus with TF binding patterns is shown in Fig. 5e, with its 3D chromatin organization Hi-C maps on larger scales shown in Additional file 1: Fig. S15.

### CTCF and NOTCH1 require each other to activate their oncogenic targets in T-ALL

**Fig. 6:**
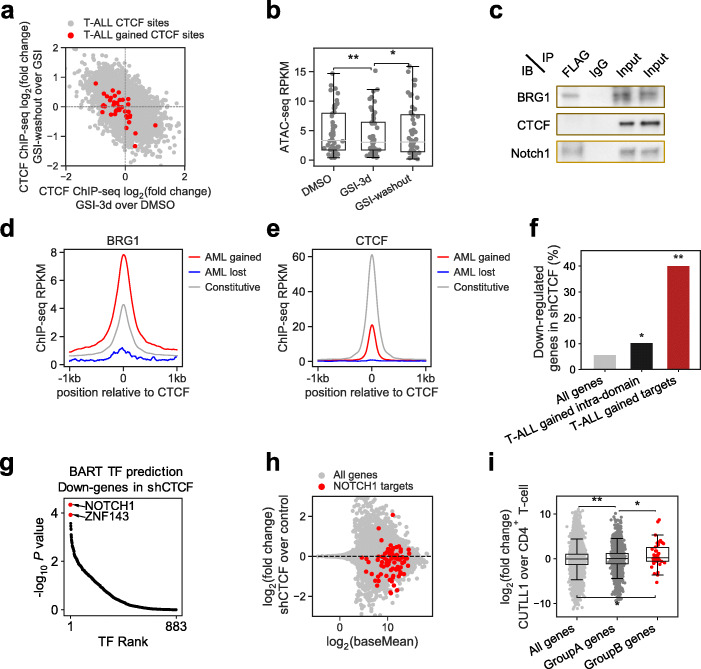
T-ALL_gained_ CTCF binding facilitates oncogenic NOTCH1 transcriptional activity. **a** Scatter plot of CTCF sites in T-ALL quantifying CTCF level changes in GSI and GSI washout experiment. Differential CTCF ChIP-seq signal (log2 fold change) in GSI washout vs. GSI (*y*-axis) is plotted against differential CTCF ChIP-seq signal in GSI vs. DMSO (*x*-axis). Red dots are T-ALL_gained_ sites. **b** ATAC-seq levels at T-ALL_gained_ CTCF sites in Jurkat cells at DMSO, GSI treated for 72 h, and GSI washout for 16 h. *, *p* < 0.05, **, *p* < 0.001, by paired two-tailed Student’s *t* test. **c** FLAG-NOTCH1 immunopurified proteins from control and NOTCH1-FLAG-expressing CUTLL1 cells were resolved on SDS-PAGE gels and interacting partners are visualized by western blot. IgG was immunopurified as a negative control. IB, immunoblot; IP, immunoprecipitation. **d**, **e** ChIP-seq signals for BRG1 (**d**) and CTCF (**e**) surrounding constitutive (gray), AML_lost_ (blue), and AML_gained_ (red) CTCF binding sites in AML cell line EOL1. Normalized ChIP-seq read counts (RPKM) covering 2-kb regions centered at CTCF binding sites were plotted per 10-bp non-overlapped bins. **f** Percentage of genes in different groups that are downregulated (log2FC < − 0.26, FDR < 0.001) in *shCTCF* experiment in CUTLL1. Black: Genes located in the T-ALL_gained_-CTCF-containing chromatin domains. Red: Genes located in the T-ALL_gained_-CTCF-containing domains that are also upregulated (log2FC > 0.26, FDR < 0.001) in T-ALL compared to normal T cell. *, *p* < 0.05, **, *p* < 0.001, by two-tailed Fisher’s exact test. **g** BART-predicted TFs that target the downregulated genes (log2FC < − 0.58, FDR < 0.01) upon *CTCF* silencing experiments in CUTLL1. **h** MA plot showing differential gene expression after *shCTCF* treatment in CUTLL1. Most NOTCH1 target genes (red) are downregulated. **i** Differential gene expression between CUTLL1 and normal T cells. Group A: genes located in dynamic-NOTCH1-containing domains. Group B: genes located in domains containing both dynamic-NOTCH1 and T-ALL_gained_ CTCF binding sites. *, *p* < 0.05, **, *p* < 0.001, by two-tailed unpaired Student’s *t* test

The association between T-ALL_gained_ CTCF binding and dynamic NOTCH1 binding suggests that CTCF might cooperate with NOTCH1 to activate gene expression in T-ALL. To test for dependency of T-ALL_gained_ CTCF binding on NOTCH1, we treated Jurkat cells with γSI for 72 h to inhibit the release and nuclear translocation of the intracellular, transcriptionally active domain of NOTCH1, and then washed out the inhibitor to allow for recovery of intracellular NOTCH1 levels for 16 h. CTCF ChIP-seq showed that γSI treatment abrogated CTCF binding at most (66%) T-ALL_gained_ sites (*P* = 1.5e−4, by *t*-test), and 68% of those γSI-sensitive binding events recovered upon γSI washout (Fig. 6a). Meanwhile, chromatin accessibility decreased at T-ALL_gained_ CTCF sites with γSI treatment compared to DMSO (*P* = 7.8e−4 by *t*-test) and significantly reversed after γSI washout (*P* = 2.2e−3 by *t*-test) (Fig. 6b). These results suggest that functional NOTCH1 binding is required for CTCF binding at T-ALL_gained_ sites.

As NOTCH1 and CTCF do not physically interact with each other (Fig. 6c) and do not co-bind at the same genomic loci (Additional file 1: Fig. S14g), we hypothesized that NOTCH1 may mediate the creation of an accessible chromatin configuration to allow for CTCF binding. Recent studies have shown that chromatin remodelers affect CTCF binding [56, 57], and NOTCH1 can interact with the catalytic subunit of the mammalian SWI/SNF chromatin remodeling complex BRG1 (SMARCA4), as well as other members of the BAF and PBAF chromatin remodeling complexes [58]. We confirmed the NOTCH1-BRG1 interaction in our T-ALL cell lines (Fig. 6c), which indicates that NOTCH1 may induce chromatin remodeling. Interestingly, BRG1 binding in the AML cell lines EOL1 and MOLM13 presents with higher enrichment at AML_gained_ CTCF sites than at constitutive CTCF sites (*P* < 1e−66) (Fig. 6d, Additional file 1: Fig. S16a) [59], although CTCF itself has lower binding levels at their corresponding gained sites in both AML and T-ALL than at constitutive sites (*P* = 0 for AML; *P* = 4.4e−9 for T-ALL) (Fig. 6e, Additional file 1: Fig. S16b,c), suggesting that BRG1 might preferentially localize to gained CTCF sites. Future work testing BRG1 function at T-ALL_gained_ sites could provide insights into whether BAF-mediated chromatin remodeling indeed occurs at these gained CTCF sites. Thus, a potential mechanism by which NOTCH1 permits T-ALL_gained_ CTCF binding could occur through BAF complex recruitment to open chromatin for CTCF binding.

The aforementioned findings suggest a potential role for T-ALL_gained_ CTCF in oncogenic transcription mediated by NOTCH1. To test whether CTCF is required for NOTCH1’s oncogenic transcription function, we knocked down CTCF with short hairpin RNAs (shRNA) in T-ALL cells (CUTLL1). Genes in the same chromatin domains containing T-ALL_gained_ CTCF binding sites, especially those genes with higher expression in T-ALL compared to normal CD4^+^ T cells, were significantly affected by CTCF silencing (odds ratio = 11.3, *P* = 4.6e−7) (Fig. 6f), indicating that T-ALL_gained_ CTCF sites are the most disrupted in our silencing study. Interestingly, BART analysis revealed that the *shCTCF*-downregulated genes are most likely regulated by NOTCH1 (*P* = 4.6e−5 from BART) (Fig. 6g). Thus, reducing CTCF levels may disrupt NOTCH1’s ability to activate its target genes. Indeed, 71% of NOTCH1 target genes in CUTLL1 are downregulated in *shCTCF* cells (Fig. 6h). Genes downregulated in *shCTCF* cells are also significantly enriched for genes downregulated in γSI-treated cells (odds ratio = 2.2, *P* = 2.6e−11) (Additional file 1: Fig. S16d), and reactivated after γSI washout (odds ratio = 3.2, *P* = 3.1e−38) (Additional file 1: Fig. S16e). These data show that CTCF is required for NOTCH1 to regulate its target genes. Additionally, we found that genes located in chromatin domains containing both dynamic NOTCH1 and T-ALL_gained_ CTCF sites are most upregulated in T-ALL compared to normal CD4^+^ T cells (effect size = 0.42, *P* = 1.7e−3) (Fig. 6i). Of these T-ALL-upregulated genes, those located in chromatin domains with increased interaction between dynamic NOTCH1 sites and T-ALL_gained_ CTCF sites are the ones whose expression is the most downregulated upon CTCF silencing (effect size = − 0.63, *P* = 0.26) (Additional file 1: Fig. S16f). Our collective findings suggest that NOTCH1 and CTCF cooperatively activate oncogenic transcriptional programs in T-ALL.

## Discussion

Through integrative analysis of multi-level genomic data collected from the public domain, we presented a comprehensive CTCF binding repertoire in the human genome, from which we identified specific CTCF binding patterns in six distinct cancer types. We characterized a series of genomic and epigenomic features of cancer-specific CTCF binding events using multi-omics profiling techniques including WGS, TF and histone modification ChIP-seq, RNA-seq, ATAC-seq, bisulfite sequencing, and in situ Hi-C. In contrast to previous studies that primarily focused on the effects of mutations or other modifications to CTCF itself or its binding sites [20, 21, 30, 47, 60, 61], we identified unique CTCF binding patterns in specific cancer types that can arise regardless of mutations or DNA methylation changes. Cancer-specific CTCF recruitment likely results from other TFs that indirectly open chromatin and alter chromatin conformation. CTCF at these sites functions cooperatively with other TFs to facilitate enhancer-promoter interactions and to activate oncogenic transcriptional programs. In T-ALL, we identified such a cooperative program occurring between NOTCH1 and CTCF, in which NOTCH1 binding is required for gained CTCF binding in the same chromatin domain. This potentially occurs through NOTCH1-induced opening of chromatin at the CTCF binding sites. Gained CTCF binding then cooperates with NOTCH1 to activate transcription of its target genes. Interestingly, we observed substantial enrichment of BRG1 at gained CTCF binding sites (Fig. 6d), as well as a direct protein-protein interaction between NOTCH1 and BRG1 (Fig. 6c). Although previous studies suggested that CTCF and BRG1 might physically interact [56], we did not find this to be the case in T-ALL (Additional file 1: Fig. S16g). Also, although direct pathway or gene ontology analysis on all genes near these identified lost/gained CTCF sites did not yield much insights into cancer functions (Additional file 1: Fig. S17), we did observe a clear pattern of differential gene expression associated with CTCF binding alteration.

**Fig. 7:**
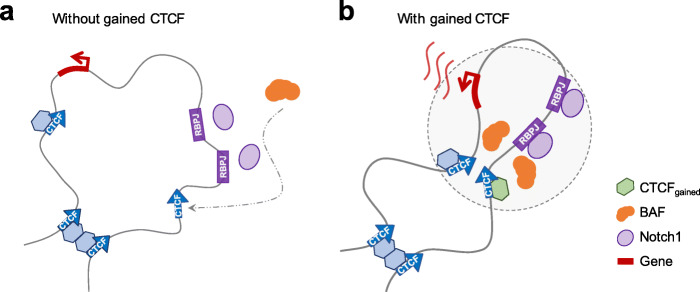
Schematic model of CTCF facilitated oncogenic transcriptional activation in T-ALL. **a** Without gained CTCF binding, intracellular NOTCH1 transcriptional complexes recognize RBPJ, the DNA-binding sequence motif, and recruit SWI/SNF / BAF complexes. **b** With gained CTCF binding, NOTCH1, BAF complexes, and CTCF protein molecules cooperatively alter the chromosome conformation and form a transcriptional condensate (dashed circle) to regulate expression of the target gene

The dynamic interactions involving multiple factors and novel CTCF binding within a single chromatin domain may indicate the formation of phase-separated transcriptional condensates at super-enhancers [62–64]. In T-ALL, NOTCH1 binding drives the establishment of super-enhancers [13]. Thus, T-ALL_gained_ CTCF binding may be recruited by clusters of TFs and co-activators including chromatin remodeling complexes within phase-separated transcriptional condensates around super-enhancers. The potential for NOTCH1 as a master TF to direct the formation of 3D spatial clusters has been reported recently [65]. Transcriptional condensates maintain a highly active environment, which is consistent with the enrichment of H3K27ac observed near T-ALL_gained_ CTCF sites (Fig. 5c). By inducing the frequency of chromatin contacts, gained CTCF binding may function to maintain the condensation state that helps drive transcription. A schematic model of the relationships between dynamic NOTCH1 binding, CTCF gain, and activation of NOTCH target genes in T-ALL is shown in Fig. 7.

Our work in T-ALL found that gains in CTCF binding are located in distal enhancer regions, while cancer-specific CTCF binding loss events are enriched at gene promoter regions and correlate with repressed transcription of these promoters and decreased chromatin interactions. Recently, an enhancer-docking mechanism described by Schuijers et al. [66] proposed that a single CTCF binding upstream of a promoter can function as a docking site for multiple distal enhancers; in this way, multiple enhancers loop to a single CTCF site to activate a single target gene promoter [66]. Loss of such a docking CTCF site then removes the ability to form these multiple enhancer loops, thus greatly reducing the ability to activate transcription. While our observations of cancer-specific lost CTCF sites are consistent with this “enhancer docking” model, further studies are required to understand the causal relationships between CTCF binding loss and gene repression.

Overall, our characterizations of identified gained/lost CTCF sites do not distinguish presence or absence of the CTCF motif. We do not discriminate CTCF binding sites based on the motif occurrence, based on a fundamental assumption that ChIP-seq data directly provide information about TF-DNA interactions, regardless of motif occurrence. We do not exclude the possibilities that many CTCF binding events may be through indirect interactions, but the function of these CTCF binding sites in inducing chromatin interactions and facilitating gene regulation is by and large similar, as shown in our analyses. In addition, it is worth noting that motif occurrence alone is not enough for TF binding. We identified 877,981 CTCF motif hits across the whole human genome. Among these hits, 639,704 (72%) are located outside any of the 688,429 union CTCF binding sites curated from ChIP-seq data. This indicates that there could still be large potential for CTCF to bind at novel loci across the genome, to play new roles in uncharacterized cell types or physiological states.

Our study is built upon integrative computational analyses of multi-source public data coupled with our multi-omics experimental validations using T-ALL as a model system. As a pan-cancer study, our work is limited by data availability and quality. Various numbers of available datasets might cause the large difference on the numbers of identified lost/gained CTCF sites across cancer types. Coverages and depths of bisulfite sequencing might lead to potential underestimation of differential DNA methylation. Although we validated that specific CTCF binding can be induced by other TFs, the causal relations between CTCF binding alteration and DNA methylation change still require further investigation in T-ALL. Also, our identified cancer-specific lost or gained CTCF sites are only a restricted portion in functional cancer epigenomes. To maintain a high specificity of CTCF binding patterns for each cancer type, we might have missed more general and commonly shared CTCF binding patterns across multiple cancers, which could be worth revisiting in the future. Our findings pave the way for further mechanistic studies of causal relationships between CTCF binding alteration and oncogenic TF activities in leukemia as well as other cancers. Following our proposed model, oncogenic drivers can lead to novel CTCF binding at distinct enhancer regions in the genome, thus creating a signature pattern of CTCF binding. Having observed evidence supporting this model in T-ALL, we believe that studying aberrant CTCF binding events in other cancer types can further our understanding of the underlying oncogenic transcriptional regulatory networks specific to that cancer. In conclusion, a unique aberrant CTCF binding pattern represents a novel epigenomic signature of cancer that can be independent of mutations or DNA methylation changes. Our work provides insights into a new angle of mechanistic research on cancer epigenomics.

## Methods

### Experimental procedure

#### Patient xenografting and cell culture

The human T-ALL cell lines include CUTLL1 (gift from Adolfo Ferrando, Columbia University) and JURKAT (American Type Culture Collection (ATCC), Manassas, VA, #CCL-119) [37, 67]. Cells were cultured in RPMI1640 medium with l-glutamine and 25 mM HEPES (Corning) supplemented with 10% heat-inactivated fetal bovine serum (Sigma-Aldrich), 10 U/mL of penicillin-streptomycin (Gibco), and 1× glutaMAX (Gibco) in a humidified incubator at 37 °C and 5% CO_2_. The cells are periodically tested for the presence of mycoplasma using the Lonza Walkersville MycoAlert Mycoplasma Detection Kit (last test in January 2020). The cell lines are kept in culture for a maximum of 20 passages and are authenticated using short-tandem repeats profiling (JURKAT) or using PCR to detect the TCRb-NOTCH1 translocation (TCRBJ2S4CUTLL1F:5′-GGACCCGGCTCTCAGTGCT-3′, NOTCH1CUTTL1R:5′-TCCCGCCCTCCAAAATAAGG-3′). Last cell authentication was performed in February 2020. Human CD4^+^ T cells were purchased from AllCells. Primary human samples were collected with informed consent and analyzed under the supervision of the Institutional Review Board of Padova University, the Associazone Italiana di Ematologia e Oncologia Pediatrica, and the Berlin-Frankfurt-Münster (AIEOP-BFM) ALL 2000/2006 pediatric clinical trials. Informed consent to use leftover material for research purposes was obtained from all of the patients at trial entry in accordance with the Declaration of Helsinki.

#### Antibodies and reagents

Western blots were performed using the following antibodies: Actin and CTCF from Millipore Sigma (clone C4; 07–729) and cleaved NOTCH1 (Val1744) from Cell Signaling Technology (4147). ChIP-seq were performed using the following antibodies: CTCF from Millipore Sigma (07-729), H3K27Ac (8173S), and H3K27me3 (9733S) from Cell Signaling Technology, and H3K4me1 (07-473) from Millipore.

#### In situ Hi-C

In situ Hi-C was performed on CD4+ T cells, Jurkat, CUTLL1, and patient xenografts as previously described [5]. In brief, cells were crosslinked with 1% formaldehyde for 10 min at room temperature. Per Hi-C reaction, 5 million cells were lysed and nuclei were permeabilized. DNA was digested with MboI from New England Biolabs (R0147M). Digested fragments were labeled with biotinylated d-ATP from Jena Bioscience (NU-835-BIO14-S) and ligated. After RNase treatment and Proteinase K treatment to reverse crosslinks, nuclei were sonicated using a Covaris E220 to produce an average fragment length of 400 bp. Streptavidin beads from Thermo Fisher Scientific (65001) were used to pull down biotin-labeled fragments. Following purification and isolation of DNA, final libraries were prepared using the NEBNext® Ultra™ II DNA Library Prep Kit for Illumina® and sequenced via paired end sequencing at a read length of 150 bp on an Illumina HiSeq 2500 to produce on average 400 million reads per sample.

#### ChIP-seq profiling

CD4+ T cells, Jurkat, CUTLL1, and patient xenografts were crosslinked with 1% formaldehyde and 1% fetal bovine serum in PBS for 10 min at room temperature. The reaction was quenched with 0.2 M glycine at room temperature for 5 min. Cells were then washed with PBS and pelleted.

For CTCF ChIPs, immunoprecipitation was performed based on a protocol described previously [68]. A pellet containing 50 million cells was lysed with 5 mL of lysis buffer (50 mM HEPES-KOH, pH 7.5, 140 mM NaCl, 1 mM EDTA, 10% glycerol, 0.5% NP-40, 0.25% Triton X-100) for 10 min at 4 °C. Nuclei were pelleted at 1350×*g* for 7 min and resuspended in 10 mM Tris pH 8, 1 mM EDTA, and 0.1% SDS. Chromatin was sheared with a Covaris E220 system to an average fragment length of 400 bp and spun at 15,000 rpm for 10 min to remove insoluble chromatin and debris. The supernatant was incubated with 20 μL of Dynabeads Protein G for 30 min before discarding the beads. One percent of the total volume was saved as input and the rest was incubated with anti-CTCF antibody overnight. In total, 100 μL of Dynabeads Protein G was added for 2 h. Bound fragments were washed twice with 1 mL of low salt buffer (20 mM Tris-HCl pH 8.0, 150 mM NaCl, 2 mM EDTA, 1% w/v Triton X-100, and 0.1% w/v SDS), once with high salt buffer (20 mM Tris-HCl pH 8.0, 500 mM NaCl, 2 mM EDTA, 1% w/v Triton X-100, and 0.1% w/v SDS), once with lithium chloride buffer (10 mM Tris-HCl pH 8.0, 250 mM LiCl, 1 mM EDTA, 1% w/v NP-40, and 1% w/v deoxycholic acid), and twice with TE (10 mM Tris pH 8, 1 mM EDTA).

For histone ChIPs, cells were lysed in 375 μL of nuclei incubation buffer (15 mM Tris pH 7.5, 60 mM KCl, 150 mM NaCl, 15 mM MgCl_2_, 1 mM CaCl_2_, 250 mM sucrose, 0.3% NP-40, 1 mM NaV, 1 mM NaF, and 1 EDTA-free protease inhibitor tablet (Roche)/10 mL in H_2_O) for 10 min on ice. Nuclei were washed once with digest buffer (10 mM NaCl, 10 mM Tris pH 7.5, 3 mM MgCl_2_, 1 mM CaCl_2_, 1 mM NaV, 1 mM NaF, and 1 EDTA-free protease inhibitor tablet (Roche)/10 mL in H_2_O) and resuspended in 57-μL Digest Buffer containing 4.5 units MNase (USB) for 1 h at 37 °C. MNase activity was quenched for 10 min on ice upon the addition of EDTA to a final concentration of 20 mM. Nuclei were pelleted and resuspended in 300-μL Nuclei Lysis Buffer (50 mM Tris-HCl pH 8.0, 10 mM EDTA pH 8.0, 1% SDS, 1 mM NaV, 1 mM NaF, and 1 EDTA-free protease inhibitor tablet (Roche)/10 mL in H_2_O) before sonication with a Bioruptor Pico (Diagenode) for 5 min (30 s on, 30 s off). Lysate was centrifuged at max speed for 5 min to remove debris. Nine volumes of IP Dilution Buffer (0.01% SDS, 1.1% Triton X-100, 1.2 mM EDTA pH 8.0, 16.7 mM Tris-HCl pH 8.0, 167 mM NaCl, 1 mM NaV, 1 mM NaF, and 1 EDTA-free protease inhibitor tablet (Roche)/10 mL in H_2_O) were added to the supernatant. In total, 50 μL of Dynabeads Protein G was added and the sample was incubated at 4 °C for 30 min, rotating. One percent of the sample was kept as input, and the remaining sample was split into 3 tubes. In total, 50 μL of Dynabeads Protein G conjugated to 15 μL of the appropriate antibody was added to each tube prior to overnight incubation at 4 °C, rotating. Bead-bound complexes were washed for 5 min each in 1 mL of low-salt buffer, high-salt buffer, LiCl buffer, and twice with TE.

To elute bead-bound complexes, beads were resuspended in 50 μL of elution buffer (100 mM NaHCO_3_, 1% w/v SDS) and incubated at 65 °C for 15 min, shaking at 1000 RPM on a thermomixer (Thermo Scientific). Elution was repeated a second time, and then 100 μL RNase Buffer (12 μL of 5 M NaCl, 0.2 μL 30 mg/mL RNase, and 88 μL TE) was added to each ChIP and input sample. Samples were incubated at 37 °C for 20 min, followed by the addition of 100 μL of proteinase K buffer (2.5 μL 20 mg/mL proteinase K, 5 μL 20% SDS, and 92.5 μL TE) overnight at 65 °C. An equal volume of phenol:chloroform solution was added and mixed thoroughly. The mixture was transferred to MaXtract High Density tubes (Qiagen) and centrifuged for 8 min at 15,000 rpm. The upper phase was transferred to new tubes and mixed with 1.5 μL 20 mg/mL glycogen, 30 μL 3M sodium acetate, and 800 μL ethanol. Samples were incubated at − 80 °C until frozen and then centrifuged at 15,000 rpm for 30 min at 4 °C. The supernatant was removed and pellets were washed in 800 μL 70% ice-cold ethanol and spun for 10 min at 4 °C at 15,000 rpm. Following careful removal of ethanol, pellets were air-dried and resuspended in 30 μL of 10 mM Tris at pH 8.

IP and input DNA were then quantified using a Qubit 3.0 fluorometer. Libraries were prepared using the KAPA HyperPrep Kit (KK8505) and sequenced with an Illumina NextSeq 500 to an average depth of 28 million reads per sample.

#### RNA-seq profiling

RNA was isolated from 3 million cells per sample using the Bio-Rad Aurum™ Total RNA Mini Kit and quantified with the Agilent RNA 6000 Nano Kit with the Agilent Bioanalyzer. Libraries were prepared by rRNA depletion using the Illumina TruSeq® Stranded mRNA Library Prep Kit for a low concentration of starting sample and sequenced by single end sequencing on an Illumina NextSeq 500 to an average depth of 18 million reads per sample.

#### DNA methylation profiling

Genomic DNA was isolated using the AllPrep DNA/RNA Micro Kit (Qiagen). To assess genome-wide DNA methylation status, we performed mRRBS [69]. Following fluorometric quantification using a Qubit 3.0 instrument, we digested genomic DNA with the restriction enzyme MspI (New England Biolabs) and size selected for fragments approximately 100–250 base pairs in length using solid phase reversible immobilization (SPRI) beads (MagBio Genomics). Resulting DNA underwent bisulfite conversion using the EZ DNA Methylation-Lightning Kit (Zymo Research). We created libraries from bisulfite-converted single-stranded DNA using the Pico Methyl-Seq Library Prep Kit (Zymo Research), which were then pooled for sequencing on an Illumina NextSeq 500 instrument using the NextSeq 500/550 V2 High Output reagent kit (1 × 75 cycles) to a minimum read depth of 50 million reads per sample.

#### Whole genome sequencing

Three million cells from cell lines or patient samples were pelleted and resuspended in 1 mL of Cell Lysis Solution (Qiagen) mixed with 500 μg of RNase A. The lysis reaction was carried out at 37 °C for 15 min. In total, 333 μL of Protein Precipitation Solution (Qiagen) was added to each sample which was then vortexed and then centrifuged at 2000×*g* for 10 min. The supernatant was mixed with 1 mL of isopropanol until DNA strands precipitated from solution. Upon discarding the supernatant, the DNA pellet was washed with 1 mL of 70% ethanol and centrifuged at 2000×*g* for 1 min. The ethanol was then poured out and the pellet was air-dried for 15 min before resuspension in 50 to 100 μL of DNA Hydration Solution (Qiagen). DNA was sequenced with paired-end Illumina sequencing at 30× coverage.

#### Immunoprecipitation

A total of 100 million cells for each immunoprecipitation reaction were pelleted and incubated in Buffer A (10 mM HEPES pH 8.0, 1.5 mM MgCl_2_, 10 mM KCl, 0.5 mM DTT) for 10 min on ice. Cells were then lysed upon 12 strokes with a 7-mL loose pestle tissue grinder (Wheaton, 357542) and centrifuged at 2000 rpm for 7 min. Nuclear pellets were resuspended in 5 volumes of TENT buffer (50 mM Tris pH 7.5, 5 mM EDTA, 150 mM NaCl, 1% Triton X-100, 5 mM MgCl_2_) and treated with benzonase for 30 min before 5 passages through a 25 g × 5/8 in. syringe. The insoluble fraction was removed following centrifugation at 2000 rpm for 7 min and incubated overnight with Dynabeads Protein G hybridized with antibody. A total of 2 million cells were removed for input. Beads and nuclei lysates were washed 6 times with TENT buffer and then eluted in 0.1 M glycine pH 2.5 with 100 mM Tris pH 8.0 prior. NuPAGE LDS sample buffer was added to eluates and inputs, which were then incubated at 70 °C for 15 min before analysis by western blot.

### Public data collection

Public CTCF ChIP-seq data were collected from Cistrome Data Browser [70] (for peak files) and NCBI GEO [71] (for fastq files, Additional file 2: Table S1). Histone modification ChIP-seq data were collected from NCBI GEO and ENCODE [72] (for bam files). Public RNA-seq data in multiple cell types were collected from ENCODE (for fastq files). DNA methylation profiling data were collected from ENCODE (for bed bedMethyl files) and NCBI GEO. Hi-C data were collected from NCBI GEO and ENCODE (for fastq files). ATAC-seq data were collected from NCBI GEO (for fastq files). Whole genome sequencing data for BRCA, COAD, LUAD, and PRAD samples were collected from International Cancer Genome Consortium (ICGC) Data Portal [49]. Detailed information including accession IDs of all public datasets collected in this work can be found in Additional file 6: Table S5.

### Data processing

#### ChIP-seq data analysis

Sequence alignment for ChIP-seq data in fastq files was performed using the same standard analysis pipeline as used in Cistrome DB [70], for consistence and reproducibility. All sequence data genomic alignment were performed using the Chilin [73] pipeline with default parameters ($ chilin simple -p narrow [--pe] -s hg38 --threads 8 -t IN.fq -i PRENAME -o OUTDIR). Briefly, sequence reads were aligned to the human reference genome (GRCH38/hg38) using BWA [74] ($ bwa aln -q 5 -l 32 -k 2 -t 8 INDEX IN.fq > PRENAME.sai $ bwa {samse | sampe} INDEX PRENAME.sai IN.fq > PRENAME.sam). Sam files were then converted into bam files using samtools [75] ($ samtools view -bS -q 1 -@ 8 PRENAME.sam > PRENAME.bam). For CTCF ChIP-seq datasets, MACS2 [76] was used to call peaks under the FDR threshold of 0.01 ($ macs2 callpeak --SPMR -B -q 0.01 --keep-dup 1 -g hs -t PRENAME.bam -n PRENAME --outidr OUTDIR). Peaks with fold enrichment of at least 4 were retained. Bigwiggle files were generated using BEDTools [77] and UCSC tools [78] ($ bedtools slop -i PRENAME.bdg -g CHROMSIZE -b 0|bedClip stdin CHROMSIZE PRENAME.bdg.clip $ LC_COLLATE=C sort -k1,1 -k2,2n PRENAME.bdg.clip > PRENAME.bdg.sort.clip $ bedGraphToBigWig PRENAME.bdg.sort.clip CHROMSIZE PRENAME.bw). Finally, only the CTCF ChIP-seq samples that have at least 2000 peaks were included in the downstream integrative analysis.

#### ATAC-seq data analysis

Trim Galore [79] was used to trim the raw sequencing reads ($ trim_galore --nextera --phred33 --fastqc --paired R1.fq R2.fq -o OUTDIR). Reads were aligned to the human reference genome (GRCH38/hg38) using Bowtie2 [80] ($ bowtie2 -p 10 -X 2000 -x INDEX -1 R1.fq -2 R2.fq -S PRENAME.sam). Sam files were then converted into bam files using samtools [75] ($ samtools view -bS -q 1 -@ 8 PRENAME.sam > PRENAME.bam). Bedtools was used to convert bam files into bed format ($ bamToBed -i PRENAME.bam -bedpe > PRENAME_PE.bed). Reads mapped to mitochondria DNA were discarded from downstream analysis.

#### RNA-seq data analysis

RNA-seq datasets were processed using Salmon [81] ($ salmon quant --gcBias -i INDEX -l A -p 8 {-1 R1.fq -2 R2.fq| -r IN.fq} -o OUTDIR). Transcriptome index was built on the human reference genome (GRCH38/hg38). Transcript-level abundance estimates were summarized to the gene level using the “tximport” [82] package for differential expression analysis. DESeq2 [83] was used to identify differentially expressed genes, and different thresholds used in different analysis were listed correspondingly in the manuscript.

#### Hi-C data analysis

Hi-C data were processed using HiC-Pro [84] ($ HiC-Pro -i INDIR -o OUTDIR -c CONFIG -p). Contact maps were generated at a resolution of 5 kb. Raw matrix data were normalized using the approach described in *Normalization of Chromatin Interactions*.

#### DNA methylation data analysis

DNA methylation data (for T-ALL cell lines and T-ALL patients) were demultiplexed with bcl2fastq followed by trimming of 10 base pairs from the 5′ end to remove primer and adaptor sequences using TrimGalore [79]. Sequence alignment to the GRCh38/hg38 reference genome and methylation calls were performed with Bismark [85] ($ bismark --multicore 8 --bowtie2 -q -N 1 INDEX INFILE.fq). Coverage (counts) files for cytosines in CpG context were generated using Bismark [85, 86] ($ bismark_methylation_extractor --multicore 8 --comprehensive --bedGraph INFILE_bismark_bt2.bam).

#### Whole genome sequencing data analysis

Mutations were identified for two T-ALL cell lines (Jurkat and CUTLL1) and two T-ALL patient samples from the whole genome sequencing data. We aligned the Illumina short-read sequences to the human reference genome (GRCH38/hg38) using BWA [74] mem. We used SAMBlaster [87] to identify the discordant pairs, split reads, and flag the putative PCR duplicates. We used SAMBAMBA [88] to convert the SAM aligned into the BAM format, and samtools [75] was used to sort those aligned to create a BAM file corresponding to each sample.

We used VarDict [89] to identify the variants that overlapped the union CTCF binding sites. We used all the default parameters except “-f 0.1” which was used to identify variants that were supported by greater than 10% of the reads at that location. We annotated the variants using Variant Effect Predictor (VEP) [90] and used custom scripts to identify the variants that influence TF binding.

We again used VarDict [89] to identify the variants in the CTCF and NOTCH1 genes for the four samples. We used all the default parameters except “-f 0.1” which was used to identify variants that were supported by greater than 10% of the reads at that location. We annotated the variants using Variant Effect Predictor (VEP) [90], and then filtered it to identify the mutations that were either (a) not seen in more than 1% of any normal human population, or (b) had a CADD score of deleteriousness > 20, or (c) was present in the COSMIC database.

### Integrative modeling and statistical analysis

#### Identification of CTCF binding repertoire in the human genome

For CTCF ChIP-seq, we collected a total of 793 datasets, including 787 public datasets and 6 datasets we generated (Additional file 2: Table S1). In total, 771 CTCF ChIP-seq datasets with peaks more than 2000 were used in this study. Each dataset can yield MACS2-identified CTCF peaks in the range between 2050 and 198,021, with a median of 46,451 and a total of 36,873,077 peaks (Additional file 1: Fig. S1a). The distribution of the interval lengths between adjacent CTCF peak summits of all 36,873,077 peaks from the 771 datasets has an inflection point at ~ 150 bp (Additional file 1: Fig. S1c) indicating the boundary between the same binding site and different binding sites [91]. Therefore, we used 150 bps as the cutoff to merge CTCF peaks. In practice, we extended ± 75 bps from each peak summit to generate a 150-bp region centered at the summit to represent each peak and merged all overlapping peak regions to generate a union set of CTCF binding sites, which contains 688,429 non-overlapping sites. Each binding site was assigned a CTCF occupancy score, defined as the tally of ChIP-seq datasets that exhibit a peak within the site. Accordingly, we defined the occupancy frequency as the ratio of the occupancy score over the total number of CTCF ChIP-seq datasets. To further ensuring the robustness of the identified CTCF binding sites, we selected 285,467 high-confidence sites with occupancy score ≥ 3 for downstream analyses. CTCF motifs within the union binding sites were searched by FIMO [92] with Jaspar [93] matrix (ID: MA0139.1), with a *p* value threshold of 1e−4. One motif with the smallest *p* value was retained for each CTCF binding site.

#### Identification of constitutive CTCF binding sites

The distribution of occupancy scores of all 285,467 CTCF binding sites (Additional file 1: Fig. S1d, blue curve) shows that the majority of the CTCF binding sites occur in only a few datasets, and the number of binding sites decreases with increasing occupancy score when the occupancy score is small. However, there are CTCF binding sites that are highly conserved across almost all datasets (e.g., binding sites with occupancy score greater than 600). We use a power law function to fit the distribution curve (blue) shown in Additional file 1: Fig. S1d to determine the cutoff for constitutive CTCF sites. We denote *O*_*i*_ as the number of observed CTCF binding sites with occupancy score equal to *i*, and *E*_*i*_ as the number of expected CTCF sites with occupancy score equal to *i*. The power law fitting to data *O*_*i*_ can be described as (Additional file 1: Fig. S1d, green):

$$ {E}_i=85767\ast {\left(i-1.37\right)}^{-1.25} $$

We define the cutoff *A* for constitutive CTCF binding sites as:

$$ A:= \min\ \left\{i|\frac{\sum_i^{771}\left({O}_i-{E}_i\right)}{\sum_i^{771}{E}_i}>5\right\} $$

In other words, the total observed CTCF sites with occupancy score greater than *A* should be 6 times more than expected. We then determined *A* = 615, and used an occupancy frequency cutoff of 80% to define 22,097 constitutive CTCF binding sites, which corresponds to the occupancy score ≥ 616 in all 771 CTCF ChIP-seq datasets.

#### Identification of cancer-specific gained/lost CTCF binding sites

We used the following 2 criteria to identify cancer-specific lost CTCF binding sites: (1) The CTCF binding site should have a lower occupancy frequency for datasets of that cancer type compared to the occupancy frequency for all datasets and (2) CTCF binding level (quantified as normalized ChIP-seq read counts) at the site is lower in cancer datasets than in other datasets. For gained CTCF sites, we used the vice versa set of criteria. Briefly, for each CTCF binding site in each cancer type, the occupancy score in the cancer datasets were calculated along with its occupancy score in all 771 datasets. CTCF binding levels were obtained from a normalized read count matrix in which the ChIP-seq read counts (RPKM) were first calculated for union CTCF binding sites in all datasets and then followed by quantile normalization. We used unpaired two-tailed Student’s *t* test to quantify the difference of binding levels between different groups of datasets, and the *p* value was then adjusted using the Benjamini-Hochberg procedure [94]. In addition, binding occupancy scores and binding levels were compared between cancer datasets and datasets from the matched normal tissue or cell types, in order to take into account the potential confounding factor of tissue specificity rather than cancer specificity. Detailed criteria for identifying cancer-specific CTCF binding sites are described below:

- *Cancer-specific lost CTCF binding sites*: (1) occupancy frequency ≤ 0.2 in cancer datasets; (2) occupancy frequency ≥ 0.7 in 771 datasets; (3) occupancy frequency ≥ 0.5 (with occupancy score ≥ 2) in matched normal tissue datasets; (4) CTCF levels are lower in cancer compared to all other datasets (statistic score < 0), (5) CTCF levels are lower in cancer compared to matched normal tissue datasets (statistic score < 0), (6) averaged CTCF binding signals (RPKM) < 5 in cancer datasets.
- *Cancer-specific gained CTCF binding sites*: (1) occupancy frequency ≥ 0.5 (with occupancy score ≥ 2) in cancer datasets, (2) occupancy frequency ≤ 0.2 in 771 datasets, (3) occupancy score = 0 in matched normal tissue datasets, (4) CTCF levels are significantly higher in cancer compared to all other datasets (FDR ≤ 0.01), (5) CTCF binding levels are significantly higher in cancer compared to matched normal tissue datasets (FDR ≤ 0.01), (6) averaged CTCF binding signals (RPKM) > 2 in cancer datasets.

The specific gained and lost CTCF binding sites for each cancer type are shown in Additional file 4: Table S3.

#### Quantification of differential chromatin accessibility

We used the processed data from Ref. [42] that include a matrix of normalized ATAC-seq insertion counts within the TCGA pan-cancer peak set to assess the differential chromatin accessibility around CTCF binding sites. For each cancer type among BRCA, CRC, LUAD, and PRAD, the pan-cancer ATAC-seq peaks that overlap with identified cancer-type-specific lost or gained CTCF binding sites were used for downstream analyses. The ATAC-seq differential score for each peak was quantified as the fold change of the average of the normalized ATAC-seq insertion counts from patient samples in the corresponding cancer type versus from patients in other cancer types, and the ATAC-seq differential score was then assigned to the peak overlapped CTCF binding site.

For consistency, we applied the same approach used for TCGA ATAC-seq data to analyze the collected ATAC-seq data from T-ALL cell line Jurkat and normal CD4+ T cells. A data matrix was generated using ATAC-seq raw read counts on union CTCF binding sites for all Jurkat and T cell datasets. Quantile normalization was applied on the log2 scaled matrix (pseudo count = 5). The ATAC-seq differential score was measured as the fold change of the averaged normalized ATAC-seq counts between datasets of Jurkat versus CD4+ T cell at each CTCF binding site.

#### Normalization of chromatin interactions

Given a Hi-C contact map *A* = {*a*_*ij*_}, the score *a*_*ij*_ reflects mapped reads between two genomic regions *i* and *j*. Suppose the bin size is 5 kb, regions *i* and *j* will have a genomic distance of ∣*i* − *j* ∣  × 5*kb*. Since the contact probability between two bins decreases with increasing genomic distance [95], we normalized the contact map as follows: for any given genomic distance *d*_*k*_ = *k* × 5*kb*, we quantify a normalization factor ${\overline{S}}_{d_k}$ as the averaged interactions among all bin pairs with the same genomic distance *d*_*k*_ in a same chromosome, e.g., ${\overline{S}}_{d_k}=\left({\sum}_{j-i=k}{a}_{ij}\right)/n$, where *n* is the total number of bin pairs with distance *d*_*k*_. The interaction score *a*_*ij*_ between two bins with distance *d*_*k*_ was then normalized by ${\overline{S}}_{d_k}$ as ${a}_{ij}^{\prime }={a}_{ij}/{\overline{S}}_{d_k}$. Using this approach, we normalized the matrix *A* into $A^{\prime }=\left\{{a}_{ij}^{\prime}\right\}$ within each chromosome.

#### Detection of differential chromatin interactions

We denoted the normalized Hi-C contact maps in the cancer dataset and the normal dataset as *C* = {*c*_*ij*_} and *N* = {*n*_*ij*_}, respectively. For a given CTCF binding site *x* (with coordinate *x*_*c*_) and a pre-defined genomic distance *L*, the chromatin interactions between *x* and its nearby non-overlapped 5-kb bins with genomic distance up to *L* are collected from *C* and *N* respectively. Specifically, interaction scores between *x* and its nearby 5-kb bins in *C* are collected as *IC* = {*c*_*ij*_} , while either *i* or *j* equals to ⌊*x*_*c*_/5*kb*⌋, and 0 < (*j* − *i*) × 5*kb* ≤ *L*. Similarly, the interaction scores between *x* and its nearby 5-kb bins in *N* were collected as *IN* = {*n*_*ij*_}. A paired two-tailed Student’s *t* test was then applied on *IC* and *IN* to quantify the differential interaction between cancer and normal cells surrounding CTCF binding site *x*.

#### Association of CTCF binding with gene expression

In total, 54 cell types for which both CTCF ChIP-seq data and RNA-seq data are publicly available were selected (Additional file 6: Table S5) for investigating the association between CTCF binding and gene expression for each CTCF-gene pair in the same chromosome. To obtain the CTCF binding level, a read count matrix was generated using reads per kilobase per million (RPKM) on union CTCF binding sites from ChIP-seq data. The read count matrix was scaled with square root of RPKM followed by quantile normalization. Gene expression level was measured for each gene using the square root of transcripts per million (TPM) from RNA-seq data. For each CTCF-gene pair, we quantified the association between the CTCF site and the gene across all 54 cell types using the correlation coefficient *R* between the normalized CTCF binding level and gene expression (Fig. 3a). CTCF-gene pairs were deemed “highly correlated” with *R*^2^ greater than 0.25, e.g., correlation coefficient greater than 0.5 or less than − 0.5, and the highly correlated CTCF-gene pairs contribute to 1.3% of all CTCF-gene pairs (Additional file 1: Fig. S8a).

#### Identification of constitutive CTCF-bounded chromatin domains

For each CTCF binding site, we defined its associated chromatin domain as the genomic region that (1) includes this specific CTCF binding site, (2) is bounded by a pair of constitutive CTCF binding sites with motifs of opposite orientations, and (3) occupies a minimum of 100 kb and a maximum of 1 MB region on each side of the CTCF binding site. Figure 3b contains schematic of how constitutive CTCF-bounded chromatin domains were defined.

#### Detection of DNA methylation changes surrounding CTCF binding sites

DNA methylation changes were detected within a 300-bp region centered at each CTCF binding site. Regions with at least 3 CpGs covered by at least 5 reads (≥ 5×) in both cancer cell lines and corresponding normal tissues were retained. A 300-bp region was detected as differentially methylated if the averaged differential methylation levels of all CpGs (≥ 5×) within this region were greater than 20% [96].

#### Detection of mutation rate and differential motif score

For each CTCF binding site, the raw mutation count was calculated as the occurrence of mutation events in all samples/patients at each single base pair within a 400-bp region centered at the CTCF binding site. The mutation rate for a group of CTCF binding sites was calculated as the averaged mutation count over the number of CTCF binding sites for each base pair within the 400-bp region.

Motif score was measured by scoring the CTCF position weight matrix (Jaspar [93], Matrix ID: MA0139.1) to a 19-bp DNA sequence centered at the CTCF motif or CTCF binding site using log likelihood ratios (with background nucleotide frequency as [0.275,0.225, 0.225, 0.275] for A,C,G,T). The differential motif score was calculated by comparing motif scores for the reference and the mutated sequences.

#### DNA sequence motif analysis

DNA sequence motif enrichment analysis was performed using MDSeqPos (version 1.0.0) on Cistrome [97] with default parameters (-cisrome -Homo Sapien or *Mus musculus*). De novo motif analyses were performed using HOMER (version 4.10) [98] with findmotifs.pl module and MEME (version 5.1.1) [99] with the following parameters: meme -dna -mod zoops -maxw 20 -evt -0.01.

#### Identification of CTCF intra-domain differentially interacted regions

For a given set of CTCF binding sites, the chromatin interaction changes between a CTCF site and each of its intra-domain non-overlapped bins, measured from normalized Hi-C contact maps in cancer cells over matched normal cells, were collected for each of the CTCF binding sites (Additional file 1: Fig. S14b). Regions with decreased interactions (log2 FC < −1, averaged log2 interaction > 0) with cancer-specific lost CTCF binding sites, and regions with increased interactions (log2 FC > 1, averaged log2 interaction > 0) with cancer-specific gained CTCF binding sites were used for downstream transcription factor (TF) enrichment analysis.

#### Transcription factor enrichment analysis

A revised version of the BART algorithm [53] was used for TF enrichment analysis. Briefly, a collection of union DNase I hypersensitive sites [100] (UDHS) was previously curated as a repertoire of all candidate cis-regulatory elements in the human genome, and 7032 ChIP-seq datasets were collected for 883 TFs [53], with each TF having one or more ChIP-seq datasets from multiple cell types or conditions. A binary profile was generated for each TF on UDHS indicating whether the TF has at least one peak from any of its ChIP-seq datasets locate within each of the UDHS. Binding enrichment analysis was applied for each TF by comparing the TF binding on a subset of UDHS overlapping the selected genomic regions versus the TF binding on UDHS. *p* value was obtained using two-tailed Fisher’s exact test.

## Supplementary information

**Additional file 1: Fig. S1.** Identification of cancer-specific CTCF binding sites. **Fig. S2.** Characterization of cancer-specific lost/gained CTCF binding sites. **Fig. S3.** Cancer-specific lost/gained CTCF binding sites associate with changed chromatin accessibility in patients. **Fig. S4.** Cancer-specific lost/gained CTCF binding sites associate with changed local chromatin interactions in different scales. **Fig. S5.** Lost/gained CTCF binding events associate with chromatin dynamics regardless of CTCF motif. **Fig. S6.** Histone modification patterns at cancer-specific lost and gained CTCF binding sites. **Fig. S7.** Histone modification patterns in normal CD4+ T-cell, T-ALL cell lines and T-ALL patients at T-ALL_lost_ and *T-ALLgained* CTCF binding sites. **Fig. S8.** Cancer-specific loss/gain of CTCF events correlate with gene expression. **Fig. S9.** Patterns of differential DNA methylation near cancer-specific lost and gained CTCF sites with and without CTCF motif. **Fig. S10.** CTCF binding loss/gain events in T-ALL cell lines and T-ALL patients do not associate with DNA sequence mutations. **Fig. S11.** CTCF binding loss/gain events in 5 cancer types do not associate with DNA sequence mutations observed in ICGC samples. **Fig. S12.** Mutation rates around lost/gained CTCF binding sites in 6 cancer types. **Fig. S13.** Sequence motif analysis on cancer-specific lost and gained CTCF binding sites. **Fig. S14.** Cancer-specific gained CTCF correlate with oncogenic transcription factor. **Fig. S15. **Hi-C interaction maps in T-ALL cell line Jurkat and CD4+ T-cell. **Fig. S16.** Cancer-specific gained CTCF binding sites correlate with oncogenic transcriptional activation. **Fig. S17.** Pathway and Gene Ontology analyses of the genes located in the same chromatin domain with gained/lost CTCF sites for each cancer.

**Additional file 2:** **Table S1.** List of collected CTCF ChIP-seq datasets.

**Additional file 3:** **Table S2.** Lists of CTCF ChIP-seq datasets in cancer cell lines and corresponding normal tissues for identification of cancer-specific CTCF binding sites.

**Additional file 4:** **Table S3.** Lists of cancer-specific lost and gained CTCF binding sites in six cancer types.

**Additional file 5:** **Table S4.** Jaccard Indexes for shared gain and Fisher *P*-values shared loss for identified CTCF sites comparing each pair of the six cancer types.

**Additional file 6:** **Table S5.** Lists of collected public multi-omics data, including ATAC-seq, DNA methylation, Hi-C, ChIP-seq and RNA-seq used in this study.

**Additional file 7:** **Table S6.** De novo motif analysis results on cancer-specific lost and gained CTCF binding sites in six cancer types, using HOMER and MEME.

**Additional file 8:** **Table S7.** BART prediction results for oncogenic TFs in T-ALL and CRC.

**Additional file 9.** Review history.

## Data Availability

The datasets generated in this study are available in NCBI GEO repository, under accession number GSE130140. https://www.ncbi.nlm.nih.gov/geo/query/acc.cgi?acc=GSE130140 (2020) [101]. The public data used and analyzed in this study are summarized in “Methods”, with the accession information included in Additional file 2: Table S1, Additional file 3: Table S2, and Additional file 6: Table S5. All source code for analyzing the data and generating the results and figures in this paper are available at GitHub, under the GNU General Public License v3.0. https://github.com/zanglab/CTCF_T-ALL_code (2020) [102].
