# Supplementary material for: Cancer-specific CTCF binding facilitates oncogenic transcriptional dysregulation
Source: Genome Biol. 2020 Sep 15;21:247. doi: 10.1186/s13059-020-02152-7 (PMC7493976; doi:10.1186/s13059-020-02152-7)
Supplement: Supplementary file 1 — Additional file 1: Fig. S1. Identification of cancer-specific CTCF binding sites. Fig. S2. Characterization of cancer-specific lost/gained CTCF binding sites. Fig. S3. Cancer-specific lost/gained CTCF binding sites associate with changed chromatin accessibility in patients. Fig. S4. Cancer-specific lost/gained CTCF binding sites associate with changed local chromatin interactions in different scales. Fig. S5. Lost/gained CTCF binding events associate with chromatin dynamics regardless of CTCF motif. Fig. S6. Histone modification patterns at cancer-specific lost and gained CTCF binding sites. Fig. S7. Histone modification patterns in normal CD4+ T-cell, T-ALL cell lines and T-ALL patients at T-ALLlost and T-ALLgained CTCF binding sites. Fig. S8. Cancer-specific loss/gain of CTCF events correlate with gene expression. Fig. S9. Patterns of differential DNA methylation near cancer-specific lost and gained CTCF sites with and without CTCF motif. Fig. S10. CTCF binding loss/gain events in T-ALL cell lines and T-ALL patients do not associate with DNA sequence mutations. Fig. S11. CTCF binding loss/gain events in 5 cancer types do not associate with DNA sequence mutations observed in ICGC samples. Fig. S12. Mutation rates around lost/gained CTCF binding sites in 6 cancer types. Fig. S13. Sequence motif analysis on cancer-specific lost and gained CTCF binding sites. Fig. S14. Cancer-specific gained CTCF correlate with oncogenic transcription factor. Fig. S15. Hi-C interaction maps in T-ALL cell line Jurkat and CD4+ T-cell. Fig. S16. Cancer-specific gained CTCF binding sites correlate with oncogenic transcriptional activation. Fig. S17. Pathway and Gene Ontology analyses of the genes located in the same chromatin domain with gained/lost CTCF sites for each cancer. [file 13059_2020_2152_MOESM1_ESM.pdf]

Additional file 1

Cancer-specific CTCF binding facilitates oncogenic transcriptional dysregulation

Celestia Fang[#], Zhenjia Wang[#], Cuijuan Han, Stephanie L. Safgren, Kathryn A. Helmin, Emmalee R. Adelman, Valentina Serafin, Giuseppe Basso, Kyle P. Eagen, Alexandre Gaspar-Maia, Maria E. Figueroa, Benjamin D. Singer, Aakrosh Ratan, Panagiotis Ntziachristos*, Chongzhi Zang*

Additional file 1 includes 17 Supplementary figures:

Fig. S1. Identification of cancer-specific CTCF binding sites.

Fig. S2. Characterization of cancer-specific lost/gained CTCF binding sites.

Fig. S3. Cancer-specific lost/gained CTCF binding sites associate with changed chromatin accessibility in patients.

Fig. S4. Cancer-specific lost/gained CTCF binding sites associate with changed local chromatin interactions in different scales.

Fig. S5. Lost/gained CTCF binding events associate with chromatin dynamics regardless of CTCF motif.

Fig. S6. Histone modification patterns at cancer-specific lost and gained CTCF binding sites.

Fig. S7. Histone modification patterns in normal CD4+ T-cell, T-ALL cell lines and T-ALL patients at $T\text{-}ALL_{lost}$ and $T\text{-}ALL_{gained}$ CTCF binding sites.

Fig. S8. Cancer-specific loss/gain of CTCF events correlate with gene expression.

Fig. S9. Patterns of differential DNA methylation near cancer-specific lost and gained CTCF sites with and without CTCF motif.

Fig. S10. CTCF binding loss/gain events in T-ALL cell lines and T-ALL patients do not associate with DNA sequence mutations.

Fig. S11. CTCF binding loss/gain events in 5 cancer types do not associate with DNA sequence mutations observed in ICGC samples.

Fig. S12. Mutation rates around lost/gained CTCF binding sites in 6 cancer types.

Fig. S13. Sequence motif analysis on cancer-specific lost and gained CTCF binding sites.

Fig. S14. Cancer-specific gained CTCF correlate with oncogenic transcription factor.

Fig. S15. Hi-C interaction maps in T-ALL cell line Jurkat and $CD4^+$ T-cell.

Fig. S16. Cancer-specific gained CTCF binding sites correlate with oncogenic transcriptional activation.

Fig. S17. Pathway and Gene Ontology analyses of the genes located in the same chromatin domain with gained/lost CTCF sites for each cancer.

Fig. S1

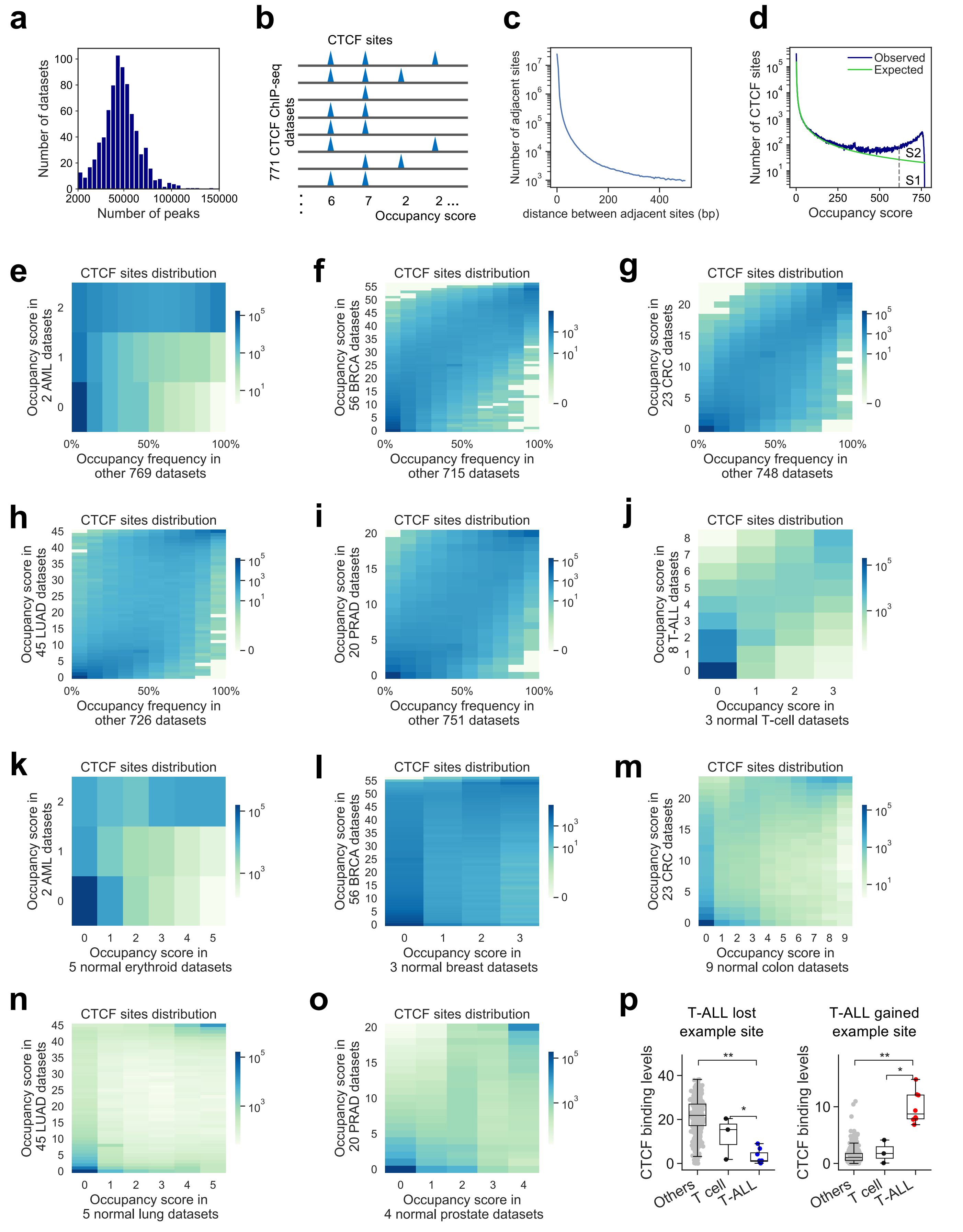
a
Number of datasets
Number of peaks
b
CTCF sites
771 CTCF ChIP-seq datasets
6
7
2
2 ...
Occupancy score
c
Number of adjacent sites
distance between adjacent sites (bp)
d
Number of CTCF sites
Observed
Expected
S2
S1
Occupancy score
e
CTCF sites distribution
Occupancy score in 2 AML datasets
Occupancy frequency in other 769 datasets
f
CTCF sites distribution
Occupancy score in 56 BRCA datasets
Occupancy frequency in other 715 datasets
g
CTCF sites distribution
Occupancy score in 23 CRC datasets
Occupancy frequency in other 748 datasets
h
CTCF sites distribution
Occupancy score in 45 LUAD datasets
Occupancy frequency in other 726 datasets
i
CTCF sites distribution
Occupancy score in 20 PRAD datasets
Occupancy frequency in other 751 datasets
j
CTCF sites distribution
Occupancy score in 8 T-ALL datasets
Occupancy score in 3 normal T-cell datasets
k
CTCF sites distribution
Occupancy score in 2 AML datasets
Occupancy score in 5 normal erythroid datasets
l
CTCF sites distribution
Occupancy score in 56 BRCA datasets
Occupancy score in 3 normal breast datasets
m
CTCF sites distribution
Occupancy score in 23 CRC datasets
Occupancy score in 9 normal colon datasets
n
CTCF sites distribution
Occupancy score in 45 LUAD datasets
Occupancy score in 5 normal lung datasets
o
CTCF sites distribution
Occupancy score in 20 PRAD datasets
Occupancy score in 4 normal prostate datasets
p
T-ALL lost example site
T-ALL gained example site
CTCF binding levels
Others
T cell
T-ALL

Fig. S1. Identification of cancer-specific CTCF binding sites.

a, Distribution of the number of identified peaks for all 771 CTCF ChIP-seq datasets. Only CTCF ChIP-seq datasets with peaks ≥2000 were included for further study.

b, Schematic of integrative analysis of CTCF binding sites from 771 ChIP-seq datasets. An occupancy score was assigned to each union binding site as the tally of ChIP-seq datasets exhibiting a peak within this binding region.

c, Distribution of the lengths of intervals between adjacent CTCF peak summits. A total of 36,873,077 CTCF peaks were collected from 771 ChIP-seq datasets.

d, Distribution of occupancy scores of all 688,429 union CTCF binding sites (blue), and a power law model fitting the distribution (green). The vertical dotted line represents the cutoff of 616 for constitutive CTCF binding sites. S1 represents the number of expected CTCF binding sites with occupancy score over 616, and S2 represents the number of observed CTCF binding sites with occupancy score over 616 excluding those model-expected ones.

e-i, Distribution of CTCF binding occupancy score in cancer cell lines (y-axis) vs. the CTCF binding occupancy frequency score in the other ChIP-seq datasets (x-axis). Color density in each element represents the number of CTCF binding sites with designated scores. **e**, AML, **f**, breast cancer (BRCA). **g**, colorectal cancer (CRC), **h**, lung cancer (LUAD), **i**, prostate cancer (PRAD).

j-o, Distribution of CTCF binding occupancy score in cancer cell lines (y-axis) vs. the occupancy score in the corresponding normal tissues (x-axis). Color density in each element represents the number of CTCF binding sites with designated scores. **j**, T-ALL, **k**, AML, **l**, breast cancer (BRCA). **m**, colorectal cancer (CRC), **n**, lung cancer (LUAD), **o**, prostate cancer (PRAD).

p, Quantile normalized CTCF read counts in normal $CD4^+$ T-cells (black), T-ALL cell lines Jurkat and CUTLL1 (blue for $T\text{-}ALL_{lost}$, red for $T\text{-}ALL_{gained}$) and the other datasets (grey) at a $T\text{-}ALL_{lost}$ site (left) and a $T\text{-}ALL_{gained}$ site (right). *, p<0.05, **, p<0.001, by two-tailed unpaired Student's *t*-test.

Fig. S2

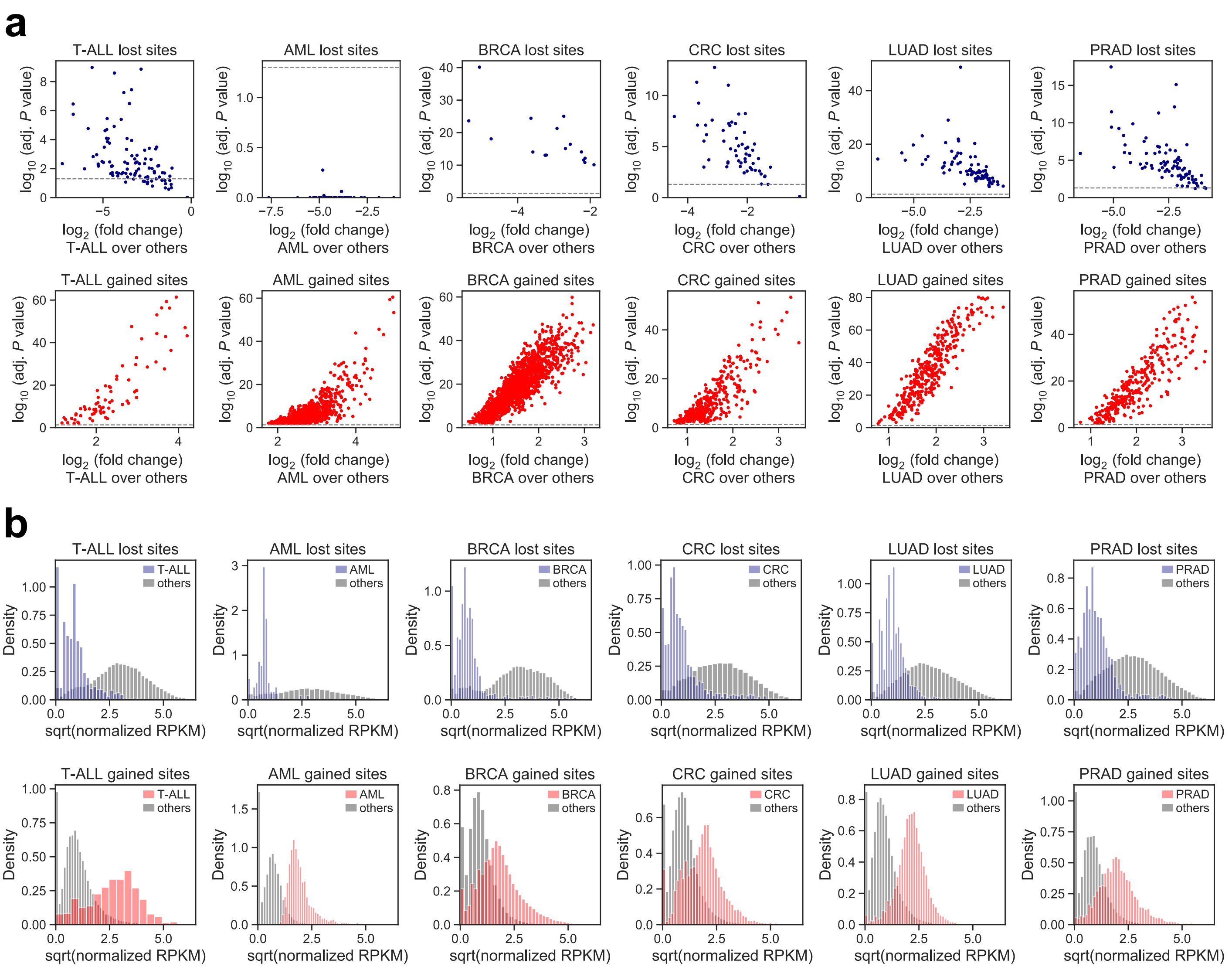

c

	effect size of lost sites	effect size of gained sites
T-ALL	-1.86	1.58
BRCA	-1.68	0.94
CRC	-1.24	0.93
LUAD	-1.52	1.62
AML	-1.87	1.69
PRAD	-1.41	1.21

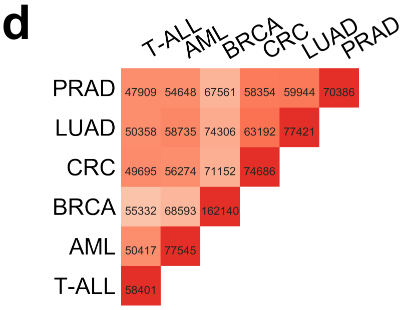

Fig. S2. Characterization of cancer-specific lost/gained CTCF binding sites.

a, Scatter plots of differential CTCF binding levels in cancer compared to all other samples for the identified cancer-specific lost (top) and gained (bottom) CTCF sites. Each data point represents a CTCF site, whose differential CTCF binding adjusted *P*-value (by Benjamini-Hochberg Procedure) (y-axis) is plotted against its binding fold change (x-axis). *P*-value was adjusted by t-test. Dashed line represents adj.*P*-value=0.05.

b, Distribution of CTCF binding levels in cancer samples and in other samples for cancer specific lost (top) and gained (bottom) sites.

c, Table of effect size of cancer specific gained and lost CTCF sites.

d, Number of shared CTCF peaks between every pair of cancer types. Color density in each square element is proportional to Jaccard Index.

Fig. S3

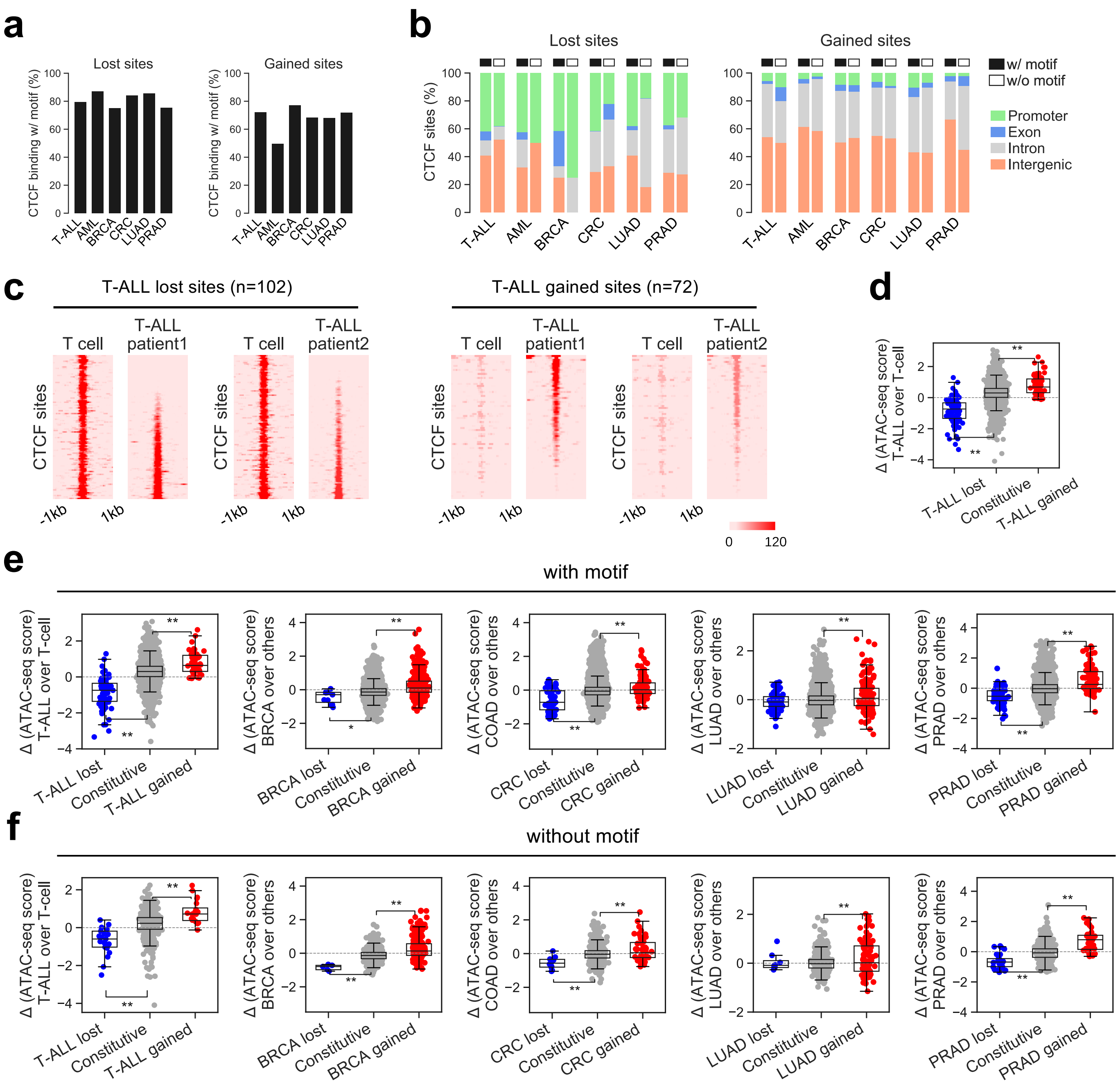

Fig. S3. Cancer-specific lost/gained CTCF binding sites associate with changed chromatin accessibility in patients.

a, Percentage of cancer specific lost (left) and gained (right) sites with CTCF motif.

b, Genomic distribution of identified lost (left) and gained (right) CTCF binding sites with or without CTCF motif in the 6 cancer types. Promoter regions are defined as +/-2kb from any TSS in the genome.

c, CTCF ChIP-seq signals at a 2kb region centered at $T\text{-}ALL_{lost}$ (left) and $T\text{-}ALL_{gained}$ (right) CTCF binding site in normal $CD4^+$ T-cells and two T-ALL patient samples.

d, Differential chromatin accessibility (ATAC-seq) in T-ALL cell line Jurkat compared to $CD4^+$ T cell at identified $T\text{-}ALL_{lost}$ (blue), $T\text{-}ALL_{gained}$ (red) and constitutive (grey) CTCF binding sites. *, $p<0.05$, **, $p<0.001$, by two-tailed unpaired Student's *t*-test.

e,f, Differential chromatin accessibility (ATAC-seq) in T-ALL cell line Jurkat compared to $CD4^+$ T cells and in TCGA patient samples comparing each cancer type to all other samples, at identified cancer-specific lost (blue), gained (red), and constitutive (grey) CTCF binding sites with (**e**) or without (**f**) CTCF motif. *, $p<0.05$, **, $p<0.001$, by two-tailed unpaired Student's *t*-test.

Fig. S4

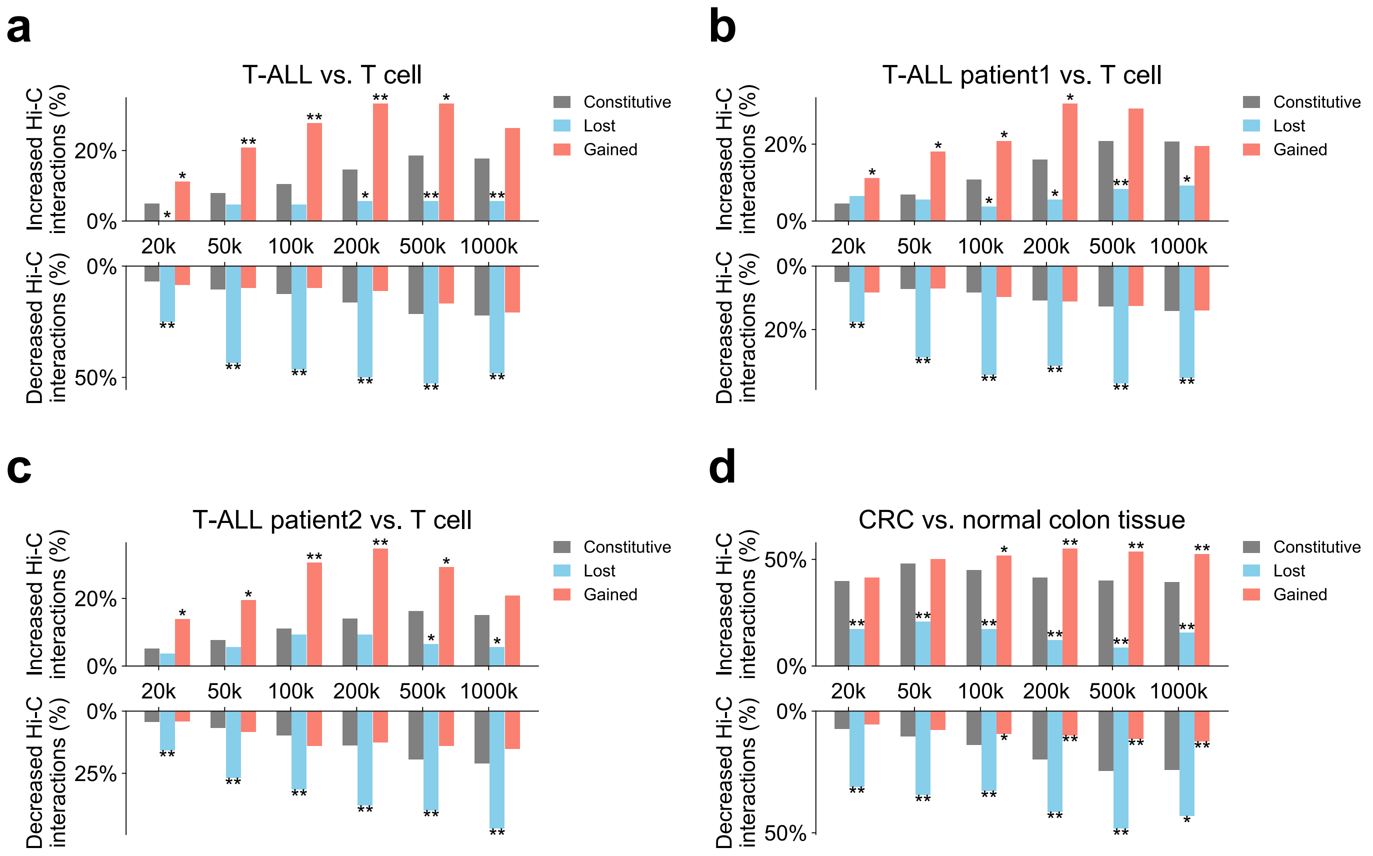

Fig. S4. Cancer-specific lost/gained CTCF binding sites associate with changed local chromatin interactions in different scales.
a-d, Percentage of cancer specific lost (blue), gained (red) and constitutive (grey) CTCF binding sites with increased (top) or decreased (bottom) local chromatin interactions in T-ALL cell line Jurkat (**a**), two T-ALL patients (**b**,**c**) and CRC (**d**) compared to corresponding normal matched tissue as observed in Hi-C. Local chromatin interactions are defined as interactions between a CTCF binding site and 5kb bins located within 20kb, 50kb, 100kb, 200kb, 500kb, 1000kb, respectively, from the CTCF site. *, p<0.05, **, p<0.001, by two-tailed Fisher's exact test.

Fig. S5

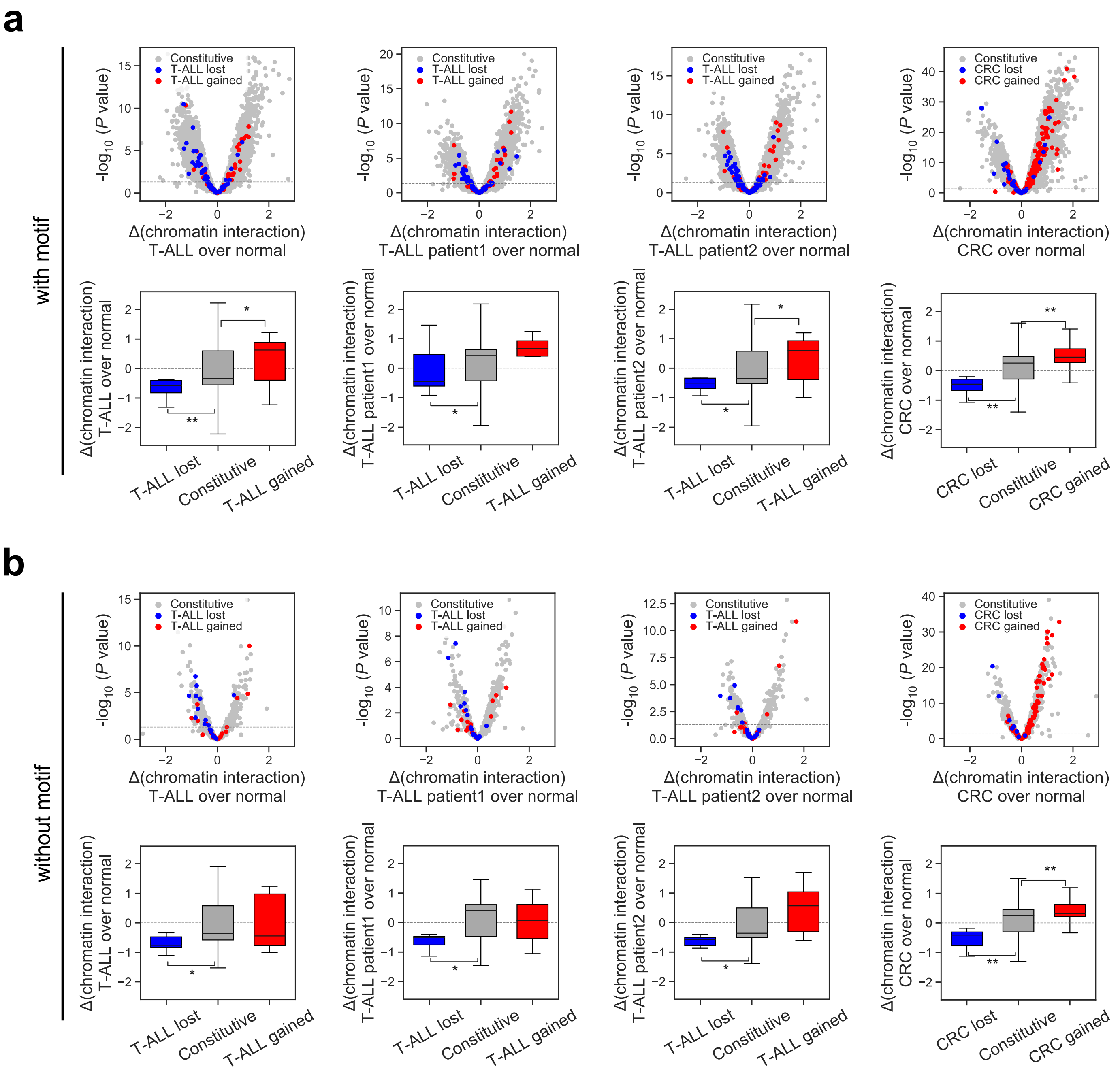

Fig. S5. Lost/gained CTCF binding events associate with chromatin dynamics regardless of CTCF motif.

a,b, Differential chromatin interaction levels between cancer and normal cells at cancer-specific CTCF sites that have (**a**) or do not have (**b**) CTCF motif. Top: Volcano plots showing differential chromatin interaction levels between cancer and normal cells at cancer-specific lost (blue), gained (red), and constitutive (grey) CTCF binding sites, measured by Hi-C. Each point represents the interaction changes between a CTCF binding site and 5kb bins located within 500kb from the site. Horizontal dotted line represents *P*-value cutoff of 0.05, by two-tailed paired Student's t-test. Bottom: Boxplots showing differential interaction frequencies between cancer and normal matched tissues for each group of CTCF binding sites. *, p<0.05, **, p<0.001, by two-tailed unpaired Student's t-test.

Fig. S6

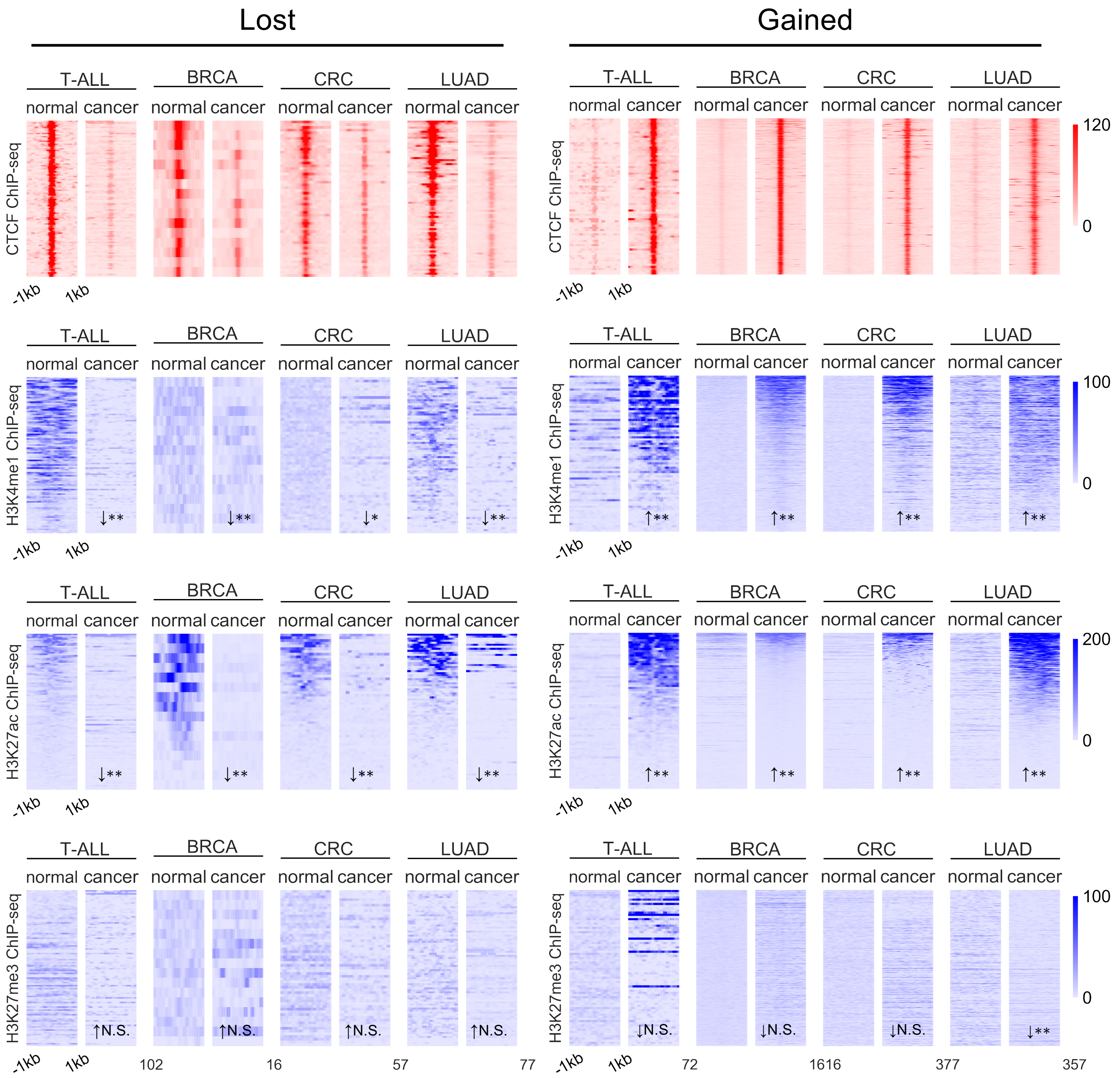

Fig. S6. Histone modification patterns at cancer-specific lost and gained CTCF binding sites.

Normalized ChIP-seq read counts of CTCF (1st row), H3K4me1 (2nd row), H3K27ac (3rd row), and H3K27me3 (4th row) surrounding identified cancer specific lost (left) and gained (right) CTCF binding sites comparing between normal tissue and cancer cell lines for T-ALL, BRCA, CRC and LUAD. ChIP-seq heatmaps cover 2kb regions centered at each CTCF site. Rows in corresponding ChIP-seq heatmaps in each cancer type are ranked identically. *, p<0.05, **, p<0.001, by one-tailed paired Student's t-test on averaged ChIP-seq levels. Arrowhead indicates the direction of the one-tailed test.

Fig. S7

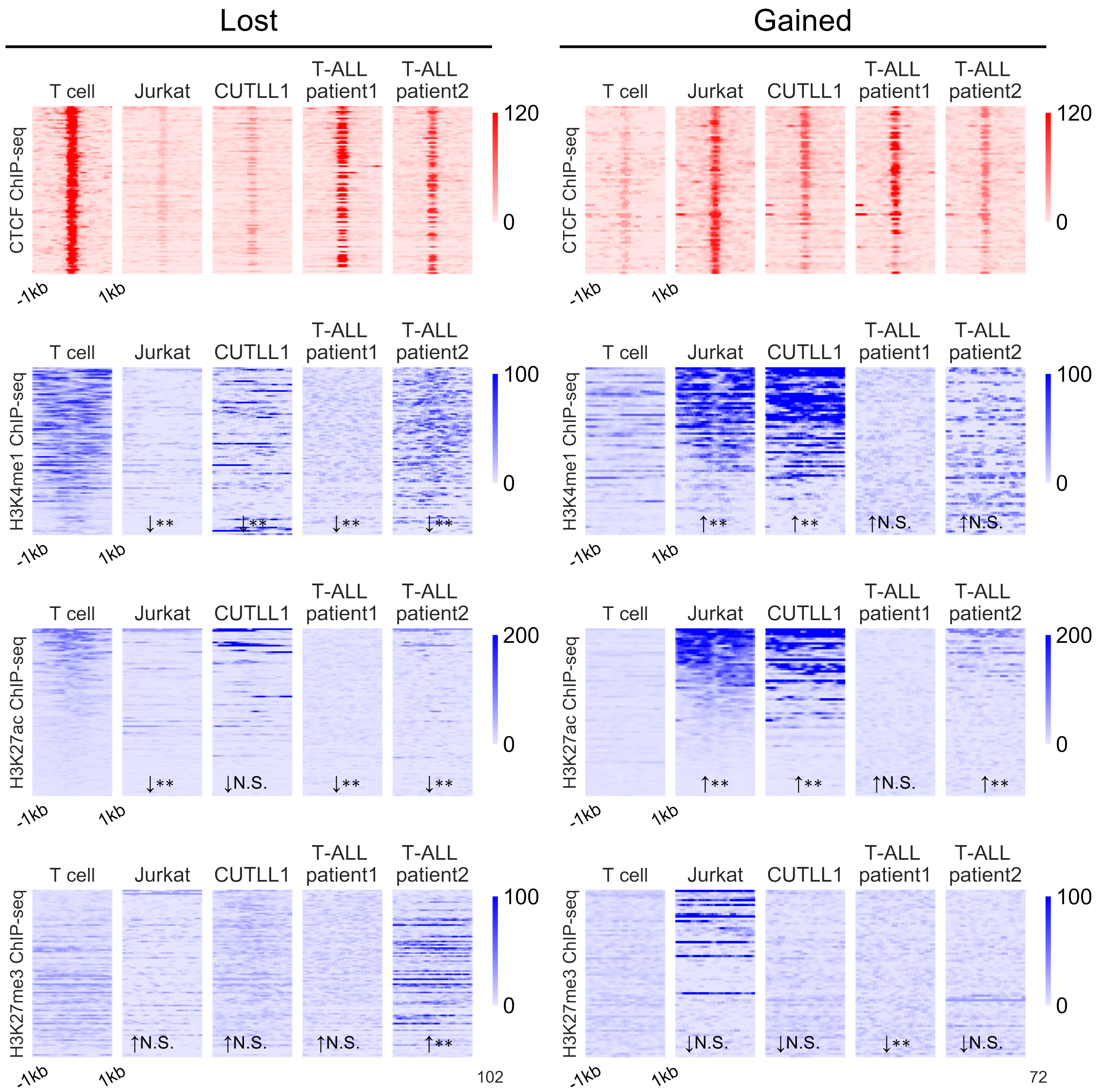

Fig. S7. Histone modification patterns in normal CD4⁺ T-cell, T-ALL cell lines and T-ALL patients at *T-ALL$_{lost}$* and *T-ALL$_{gained}$* CTCF binding sites.
Normalized ChIP-seq read counts of CTCF (1st row), H3K4me1 (2nd row), H3K27ac (3rd row), and H3K27me3 (4th row) surrounding identified *T-ALL$_{lost}$* (left) and *T-ALL$_{gained}$* (right) CTCF binding sites comparing normal CD4$^+$ T-cells, two T-ALL cell lines Jurkat and CUTLL1, and two T-ALL patient samples. ChIP-seq heatmaps cover 2kb regions centered at each CTCF site. Rows in corresponding ChIP-seq heatmaps are ranked identically. *, $p<0.05$, **, $p<0.001$, by one-tailed paired Student's t-test on averaged ChIP-seq levels. Arrowhead indicates the direction of the one-tailed test.

Fig. S8

a

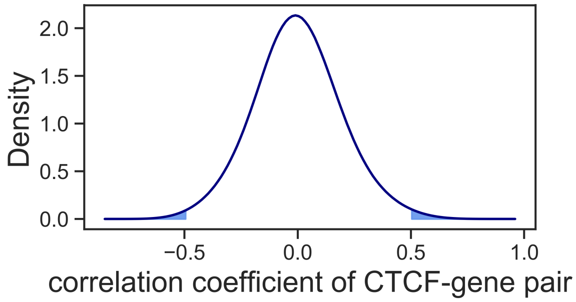

b

	No. of CTCF	No. of merged region	No. of merged region overlapped w/ HiC boundary	No. of HiC boundary (total 2947) overlapped w/ merged region
union	285,467	101	53 (52.5%)	2,946 (100.0%)
constitutive	22,097	2,295	1,753 (76.4%)	2,544 (86.3%)
boundary	13,771	2,842	2,064 (72.6%)	2,482 (84.2%)

c

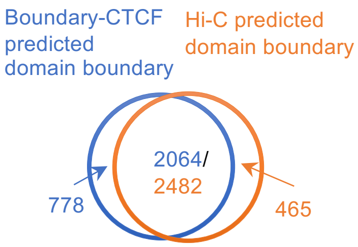

d

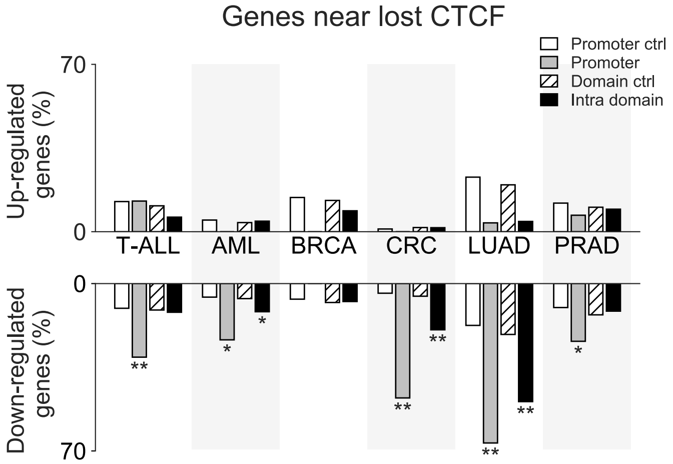

e

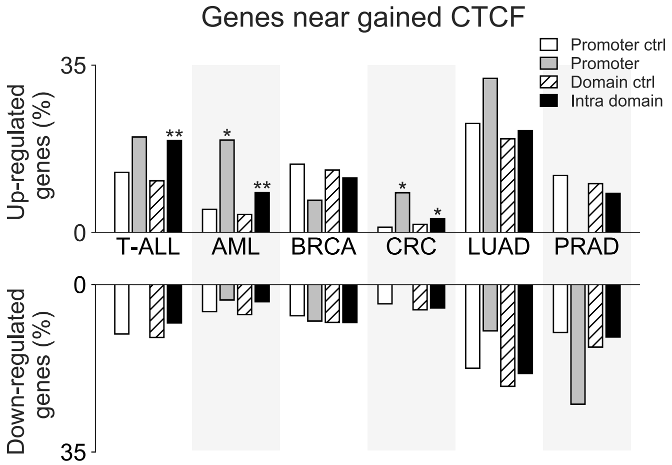

f

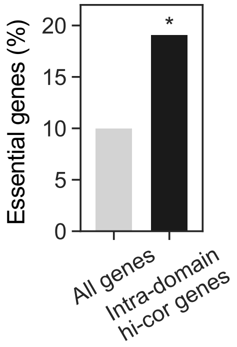

Fig. S8. Cancer-specific loss/gain of CTCF events correlate with gene expression.
a, Distribution of correlation coefficient values of all CTCF-gene pairs in the genome. Light blue shaded areas represent highly correlated pairs with correlation coefficient values greater than 0.5 or less than -0.5.
b, Comparison of union CTCF, constitutive CTCF and boundary CTCF defined domain boundaries and Hi-C map defined domain boundaries. Boundary CTCF sites are defined as constitutive CTCFs that are further used as domain boundaries. 40kb extended CTCF sites located within 200kb with each other were merged to generate the merged region in each group.
c, Venn diagram comparing boundary CTCF defined domain boundaries and Hi-C map defined domain boundaries.
d,e, Percentage of genes that are up-regulated (top, log2FC>1, FDR<1e-5) or down-regulated (bottom, log2FC<-1, FDR<1e-5) located in the chromatin domains containing cancer specific lost (**d**) or gained (**e**) CTCF binding sites in T-ALL, AML, BRCA, CRC, LUAD and PRAD. "Promoter" refers to genes whose promoter region (TSS +/-2kb) contains a CTCF binding site from a certain category. "Promoter ctrl" refers to genes whose promoter region contains a constitutive CTCF binding site as the control for cancer-specific gained/lost sites. "Intra-domain" refers to genes whose chromatin domain contains a CTCF binding site. "Domain ctrl" refers to genes whose chromatin domain contains a constitutive CTCF site as the control for those with cancer-specific gained/lost sites. *, p<0.05, **, p<0.001, by two-tailed Fisher's exact test.
f, Percentage of genes in each group that are essential genes in T47D cells. Essential genes are defined as genes with lowest β-scores from genome-wide CRISPR screens. Black, genes located in the chromatin domain containing $BRCA_{gained}$ CTCF sites and are highly correlated with the CTCF sites. *, p<0.05, **, p<0.001, by two-tailed Fisher's exact test.

Fig. S9

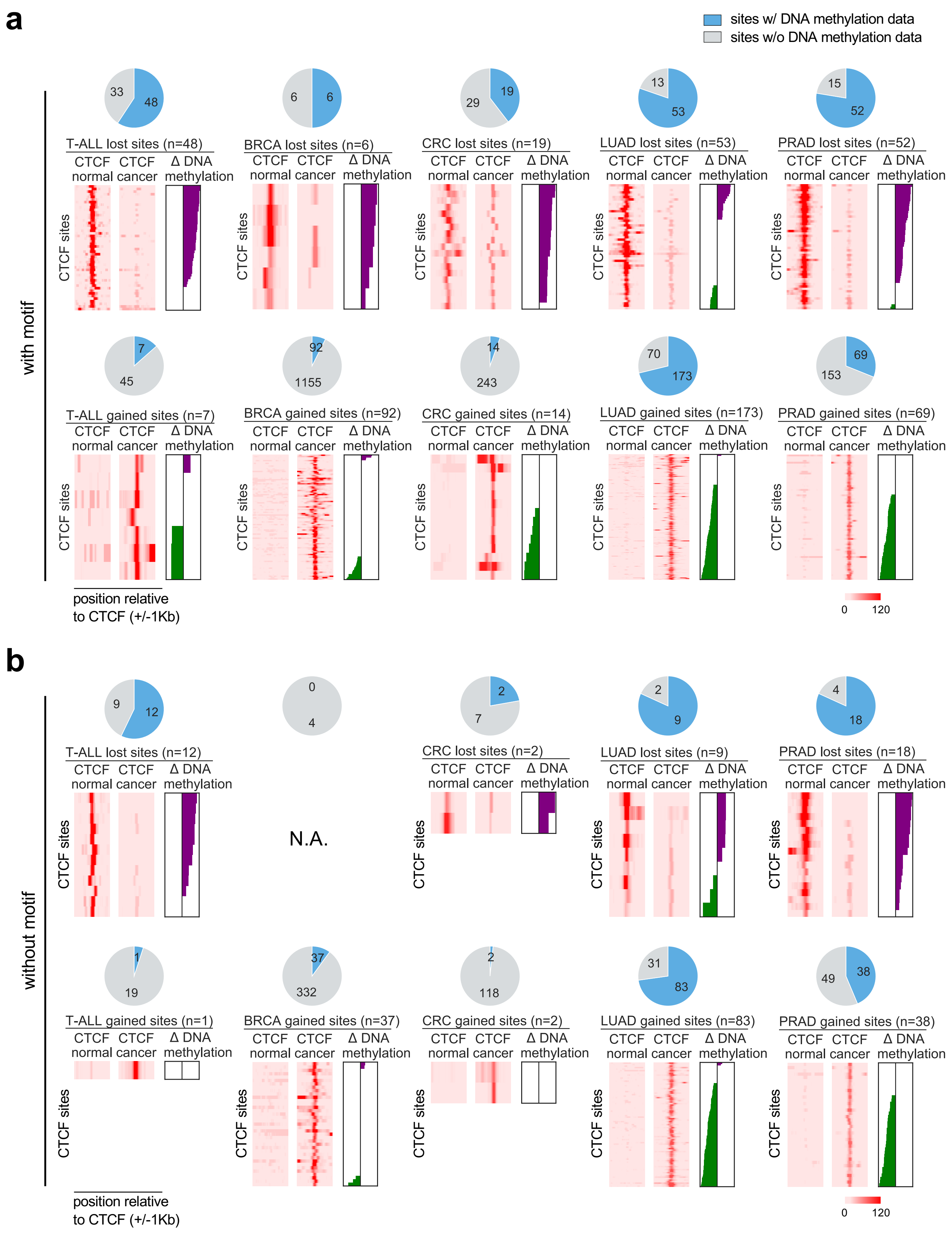
a
sites w/ DNA methylation data
sites w/o DNA methylation data
with motif
33
48
T-ALL lost sites (n=48)
6
6
BRCA lost sites (n=6)
29
19
CRC lost sites (n=19)
13
53
LUAD lost sites (n=53)
15
52
PRAD lost sites (n=52)
CTCF normal
CTCF cancer
Δ DNA methylation
CTCF sites
7
45
T-ALL gained sites (n=7)
92
1155
BRCA gained sites (n=92)
14
243
CRC gained sites (n=14)
70
173
LUAD gained sites (n=173)
69
153
PRAD gained sites (n=69)
position relative to CTCF (+/-1Kb)
0
120
b
without motif
9
12
T-ALL lost sites (n=12)
0
4
N.A.
2
7
CRC lost sites (n=2)
2
9
LUAD lost sites (n=9)
4
18
PRAD lost sites (n=18)
1
19
T-ALL gained sites (n=1)
37
332
BRCA gained sites (n=37)
2
118
CRC gained sites (n=2)
31
83
LUAD gained sites (n=83)
49
38
PRAD gained sites (n=38)
position relative to CTCF (+/-1Kb)
0
120

Fig. S9. Patterns of differential DNA methylation near cancer-specific lost and gained CTCF sites with and without CTCF motif.

a,b, ChIP-seq signals and differential DNA methylation levels surrounding specific lost (top) or gained (bottom) CTCF binding sites with (**a**) or without (**b**) CTCF motif in cancer versus matched normal tissues for each of the 5 cancer types. Top: Pie chart of binding sites with or without sufficient DNA methylation data. Bottom: ChIP-seq heatmaps cover 2kb regions centered at each CTCF site with sufficient DNA methylation data. Differential DNA methylation plots cover 300bp regions centered at each CTCF site. Purple bars represent increased and green bars represent decreased DNA methylation levels (with values in a range from 0 to 100). Rows in corresponding ChIP-seq and DNA methylation plots are ranked identically. N.A., no CTCF sites with sufficient DNA methylation data.

Fig. S10

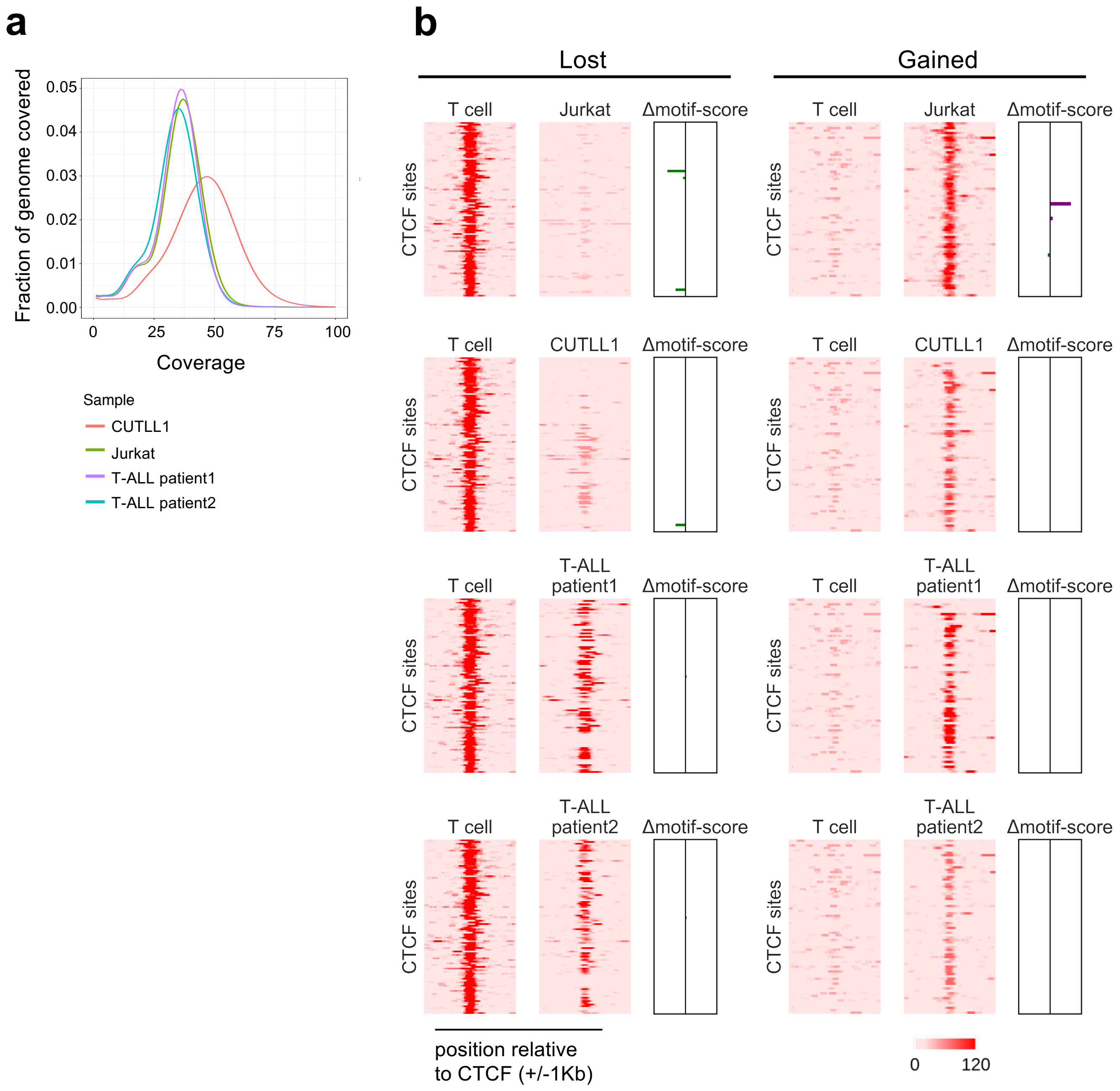

Fig. S10. CTCF binding loss/gain events in T-ALL cell lines and T-ALL patients do not associate with DNA sequence mutations.
a, Sequencing coverage distribution for WGS data in T-ALL cell lines Jurkat and CUTLL1 and two T-ALL patient samples.
b, CTCF ChIP-seq signals and motif score changes surrounding $T\text{-}ALL_{lost}$ (left) and $T\text{-}ALL_{gained}$ (right) CTCF sites comparing normal $CD4^+$ T-cell, two T-ALL cell lines Jurkat and CUTLL1 and two T-ALL patients. ChIP-seq heatmaps cover 2kb regions centered at each CTCF site. Differential motif score plots cover 19bp CTCF motif sequences. Purple bars represent increased and green bars represent decreased motif score (with values in a range from 0 to 6). Rows in corresponding ChIP-seq heatmaps and differential motif score plots are ranked identically.

Fig. S11

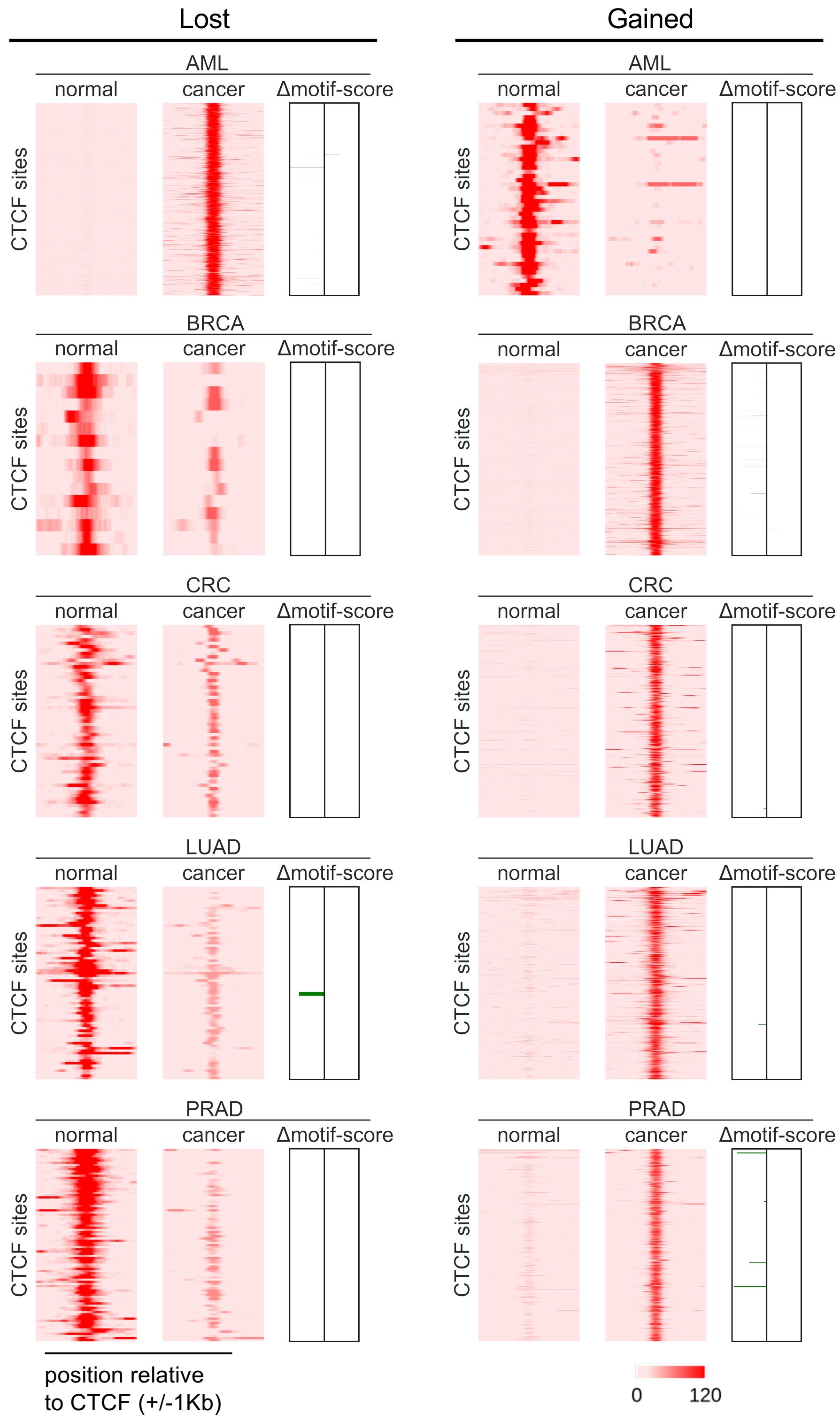

Fig. S11. CTCF binding loss/gain events in 5 cancer types do not associate with DNA sequence mutations observed in ICGC samples.

CTCF ChIP-seq signals and motif score changes surrounding cancer specific lost (left), and gained (right) CTCF sites comparing normal tissues and cancers in each of the 5 cancer types of AML, BRCA, CRC, LUAD and PRAD. ChIP-seq heatmaps cover 2kb regions centered at each CTCF site. Differential motif score plots cover 19bp motif sequences. Purple bars represent increased and green bars represent decreased motif score (with values in a range from 0 to 9). Rows in corresponding ChIP-seq heatmaps and differential motif score plots are ranked identically.

Fig. S12

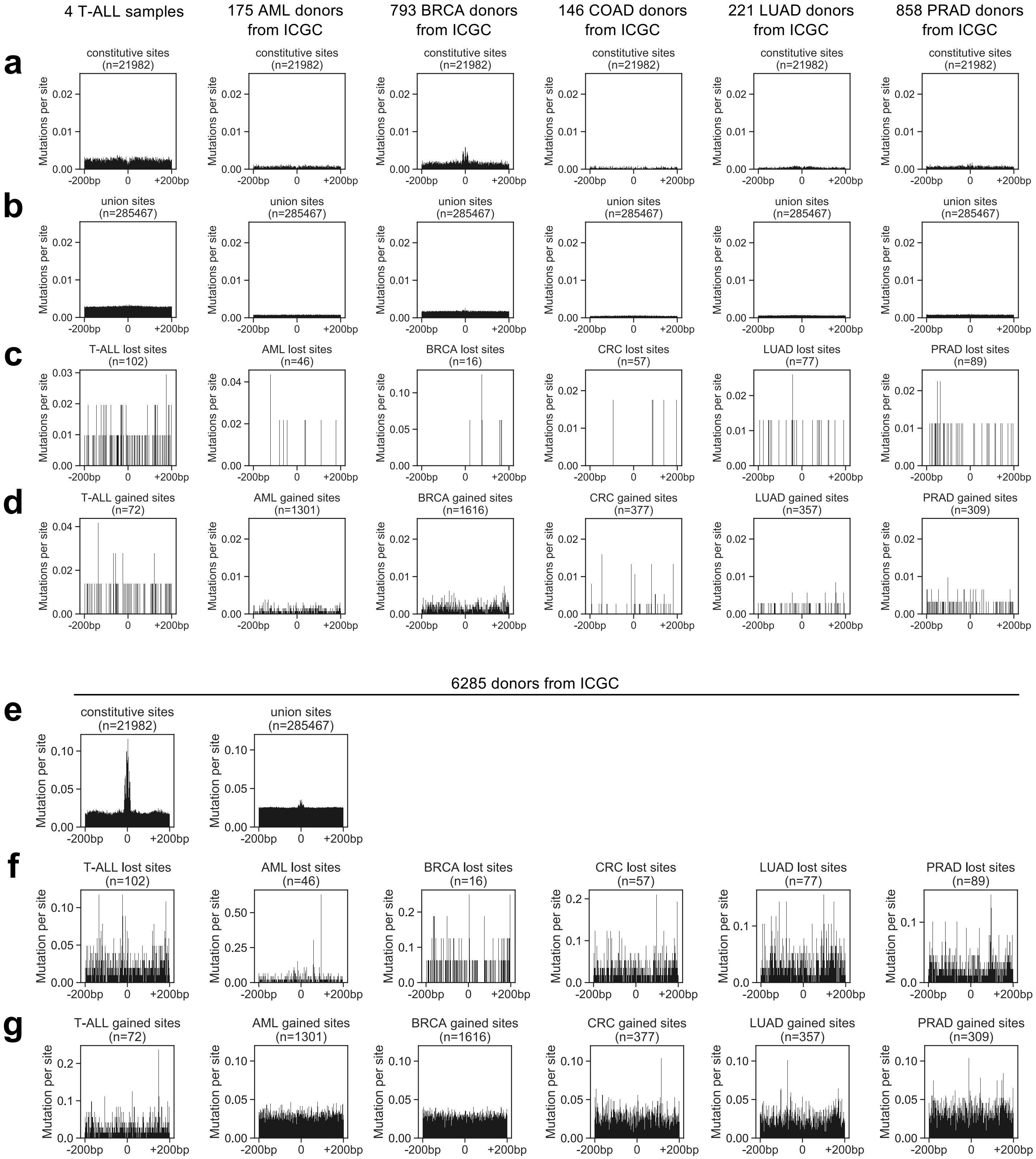
4 T-ALL samples
175 AML donors from ICGC
793 BRCA donors from ICGC
146 COAD donors from ICGC
221 LUAD donors from ICGC
858 PRAD donors from ICGC
a
constitutive sites (n=21982)
b
union sites (n=285467)
c
T-ALL lost sites (n=102)
AML lost sites (n=46)
BRCA lost sites (n=16)
CRC lost sites (n=57)
LUAD lost sites (n=77)
PRAD lost sites (n=89)
d
T-ALL gained sites (n=72)
AML gained sites (n=1301)
BRCA gained sites (n=1616)
CRC gained sites (n=377)
LUAD gained sites (n=357)
PRAD gained sites (n=309)
Mutations per site
-200bp
0
+200bp
6285 donors from ICGC
e
constitutive sites (n=21982)
union sites (n=285467)
f
T-ALL lost sites (n=102)
AML lost sites (n=46)
BRCA lost sites (n=16)
CRC lost sites (n=57)
LUAD lost sites (n=77)
PRAD lost sites (n=89)
g
T-ALL gained sites (n=72)
AML gained sites (n=1301)
BRCA gained sites (n=1616)
CRC gained sites (n=377)
LUAD gained sites (n=357)
PRAD gained sites (n=309)
Mutation per site

Fig. S12. Mutation rates around lost/gained CTCF binding sites in 6 cancer types.
a-d, Averaged mutation rate for each specific cancer type: Jurkat/CUTLL1 cell lines and two patient samples for T-ALL, ICGC AML/LAML for AML, ICGC BRCA for BRCA, ICGC COAD/COCA/READ for CRC, ICGC LUAD/LUSC for LUAD, and ICGC PRAD/EOPC for PRAD, surrounding constitutive (**a**), union (**b**), cancer-specific lost (**c**) and gained (d) CTCF sites. Mutation rate plots cover 400bp regions centered at each CTCF binding site.
e-g, Averaged mutation rate for all 6285 donors from ICGC database, surrounding constitutive and union (**e**), cancer-specific lost (**f**) and gained (**g**) CTCF sites. Mutation rate plots cover 400bp regions centered at each CTCF binding site.

Fig. S13

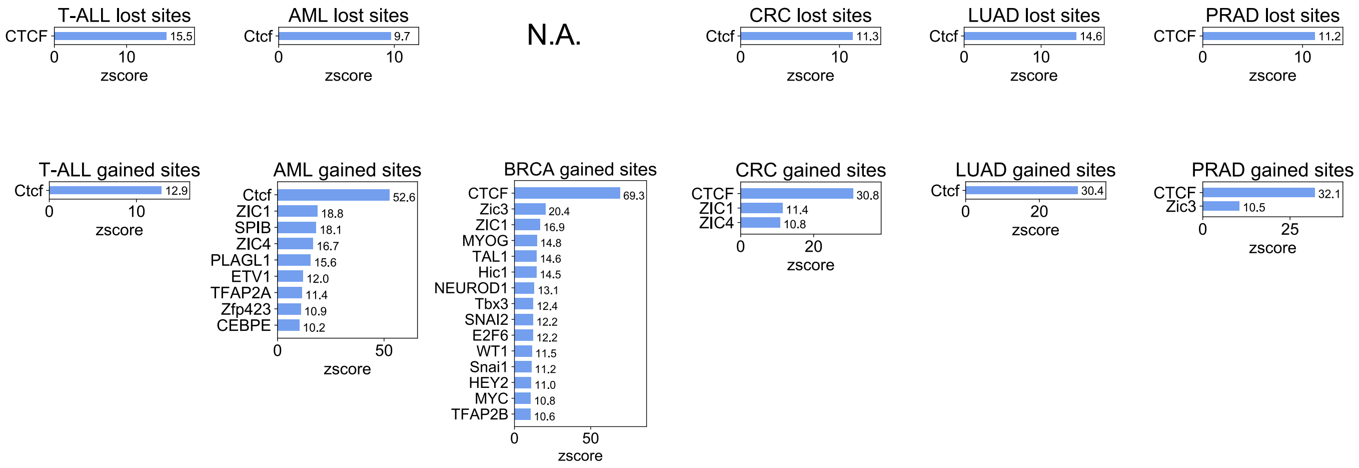

Fig. S13. Sequence motif analysis on cancer-specific lost and gained CTCF binding sites. MDSeqPos motif analysis results on cancer specific lost (top row) and gained (bottom row) CTCF sites in each cancer type. Motifs with Z-score >=10 were shown in each dataset. N.A., no motifs were reported.

Fig. S14

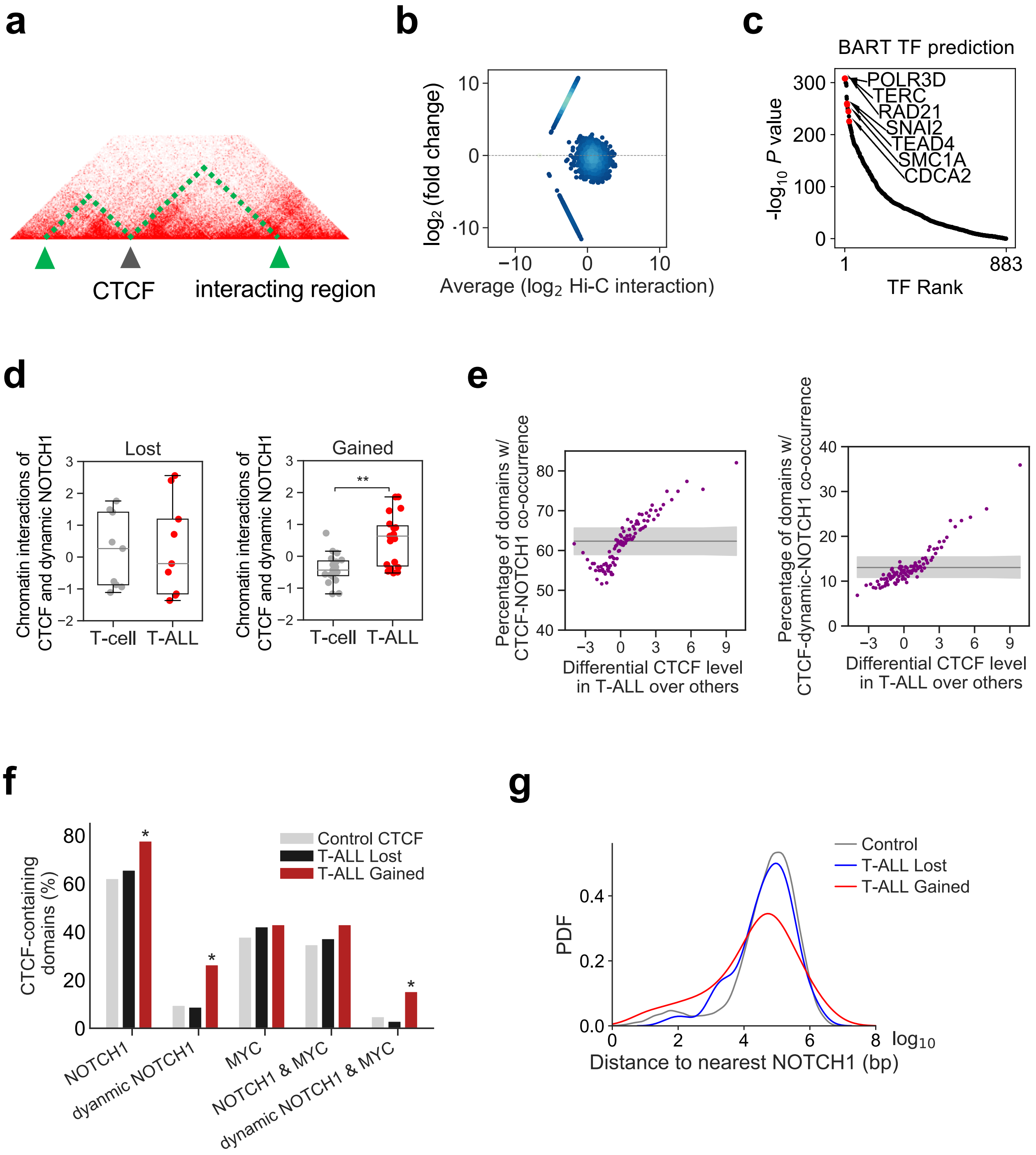
a
CTCF
interacting region
b
log2 (fold change)
Average (log2 Hi-C interaction)
c
BART TF prediction
POLR3D
TERC
RAD21
SNAI2
TEAD4
SMC1A
CDCA2
-log10 P value
TF Rank
d
Lost
Gained
Chromatin interactions of CTCF and dynamic NOTCH1
T-cell
T-ALL
**
e
Percentage of domains w/ CTCF-NOTCH1 co-occurrence
Percentage of domains w/ CTCF-dynamic-NOTCH1 co-occurrence
Differential CTCF level in T-ALL over others
f
CTCF-containing domains (%)
Control CTCF
T-ALL Lost
T-ALL Gained
NOTCH1
dyanmic NOTCH1
MYC
NOTCH1 & MYC
dynamic NOTCH1 & MYC
g
Control
T-ALL Lost
T-ALL Gained
PDF
log10
Distance to nearest NOTCH1 (bp)

Fig. S14. Cancer-specific gained CTCF correlate with oncogenic transcription factor.
a,b, Schematic of identification of cis-domain genomic regions that have changed chromatin interactions with cancer specific gained/lost CTCF sites comparing cancer cell lines and matched normal tissues. **a**, Chromatin interactions between a CTCF binding site and all of its intra-domain 5kb bins. **b**, MA plot showing differential chromatin interactions between cancer and normal cells at cancer specific CTCF binding sites. Each point represents the chromatin interaction changes between a CTCF site and one of its intra-domain 5kb bin.
c, BART-predicted transcription factors binding in the genomic regions that have increased interaction with CRC_{gained} CTCF sites comparing HCT116 cell line with the normal colon tissue.
d, Chromatin interaction levels between $T\text{-}ALL_{lost}$ (left) and $T\text{-}ALL_{gained}$ (right) CTCF binding site and their intra-domain dynamic NOTCH1 binding sites comparing normal $CD4^{+}$ T-cell (grey) and T-ALL cell line CUTLL1 (red) as measured by Hi-C. Chromatin interaction between a CTCF site and a dynamic NOTCH1 site was quantified as a Z-score using interactions between the CTCF site with all of its intra-domain regions as background. *, $p<0.05$, **, $p<0.001$, by two-tailed paired Student's *t*-test.
e, Scatter plots showing global association between T-ALL-specific CTCF and enrichment of NOTCH1 (left) or dynamic NOTCH1 (right) bindings within the chromatin domain. All CTCF binding sites in T-ALL cells were categorized into 100 groups based on their differential levels in T-ALL compared to other samples. In each group, the percentage of CTCF sites that have NOTCH1 binding (left) or dynamic NOTCH1 binding (right) within the same domain (y-axis) is plotted against the median differential CTCF binding level comparing T-ALL to other samples (x-axis). The horizontal line represents the average percentage and the grey shadow region represents the 95% confidence interval of finding CTCF-NOTCH1 co-occurrence if a group of CTCF sites were randomly sampled from the population based on a hypergeometric distribution.
f, Percentage of chromatin domains including different groups of CTCF binding sites that contain a NOTCH1 binding site, a dynamic NOTCH1 binding site, a MYC binding site, a NOTCH1 together with a MYC binding site, and a dynamic NOTCH1 together with a MYC binding site, respectively. *, $p<0.05$, **, $p<0.001$, by two-tailed Fisher's exact test.
g, Distribution of the distances between the CTCF binding sites in different groups and their nearest NOTCH1 binding sites in T-ALL cell line CUTLL1.

Fig. S15

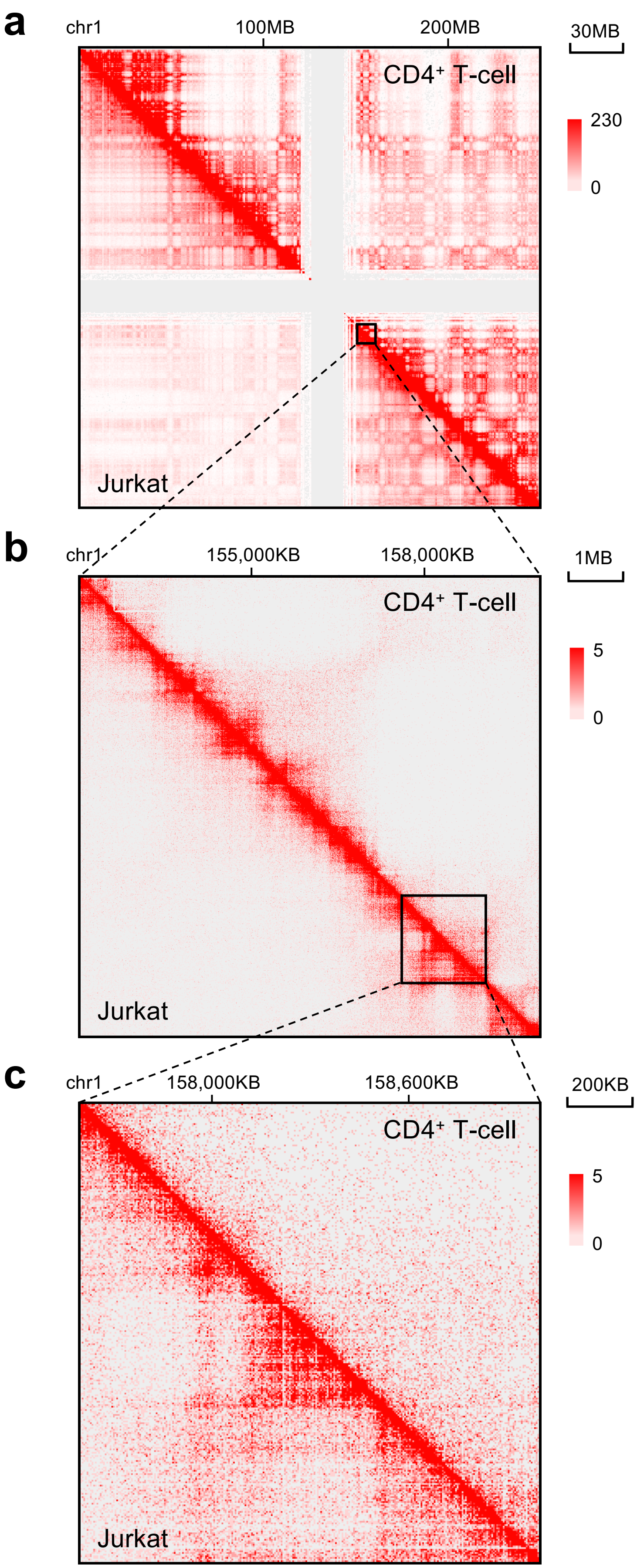

Fig. S15. Hi-C interaction maps in T-ALL cell line Jurkat and CD4⁺ T-cell.
a,b,c, Hi-C interaction maps comparing Jurkat and CD4⁺ T-cell in different scales: (**a**) whole chromosome 1, (**b**) MBs level and (**c**) hundred KBs level.

Fig. S16

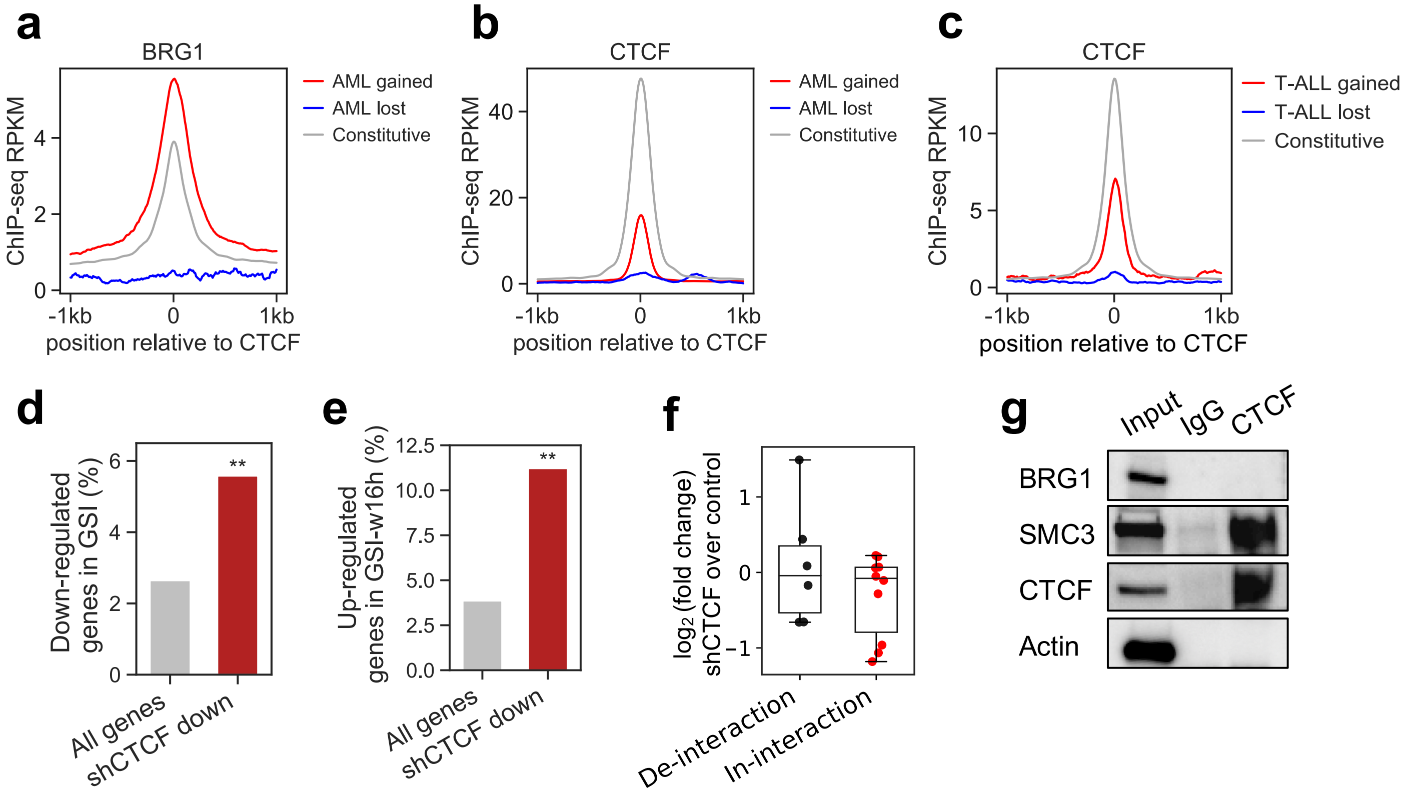

Fig. S16. Cancer specific gained CTCF binding sites correlate with oncogenic transcriptional activation.

a,b ChIP-seq signals for BRG1 (**a**) and CTCF (**b**) surrounding constitutive (grey), AML_{lost} (blue) and AML_{gained} (red) CTCF binding sites in AML cell line MOLM13. Normalized ChIP-seq read counts (RPKM) covering 2kb regions centered at CTCF binding sites were plotted per 10bp non-overlapped bins.

c, ChIP-seq signals for CTCF surrounding constitutive (grey), $T\text{-}ALL_{lost}$ (blue) and $T\text{-}ALL_{gained}$ (red) CTCF binding sites in T-ALL cell line Jurkat. Normalized ChIP-seq read counts (RPKM) covering 2kb regions centered at CTCF binding sites were plotted per 10bp non-overlapped bins.

d,e, Down-regulated genes after GSI treatment (**d**) and up-regulated genes after GSI washout treatment (**e**) are enriched for down-regulated genes in shCTCF treatment in T-ALL cell line CUTLL1. Differentially expressed genes were identified using thresholds of |log2FC|>0.58, FDR<0.01. *, p<0.05, **, p<0.001, by two-tailed Fisher's exact test.

f, Differential expression upon shCTCF for genes located in domains containing both dynamic-NOTCH1 and $T\text{-}ALL_{gained}$ CTCF binding sites and are up-regulated in CUTLL1 compared to $CD4^+$ T cell. Genes were separated into two groups by having their intra-domain dynamic NOTCH1 and $T\text{-}ALL_{gained}$ CTCF sites with decreased (black) or increased (red) chromatin interactions in CUTLL1 compared to $CD4^+$ T cell .

g, CTCF immunopurified proteins from Jurkat cells were resolved on SDS-PAGE gels and interacting partners are visualized by western blot. IgG was immunopurified as a negative control, and SMC3 was immunoblotted as a positive control. IB, immunoblot; IP, immunoprecipitation.

Fig. S17

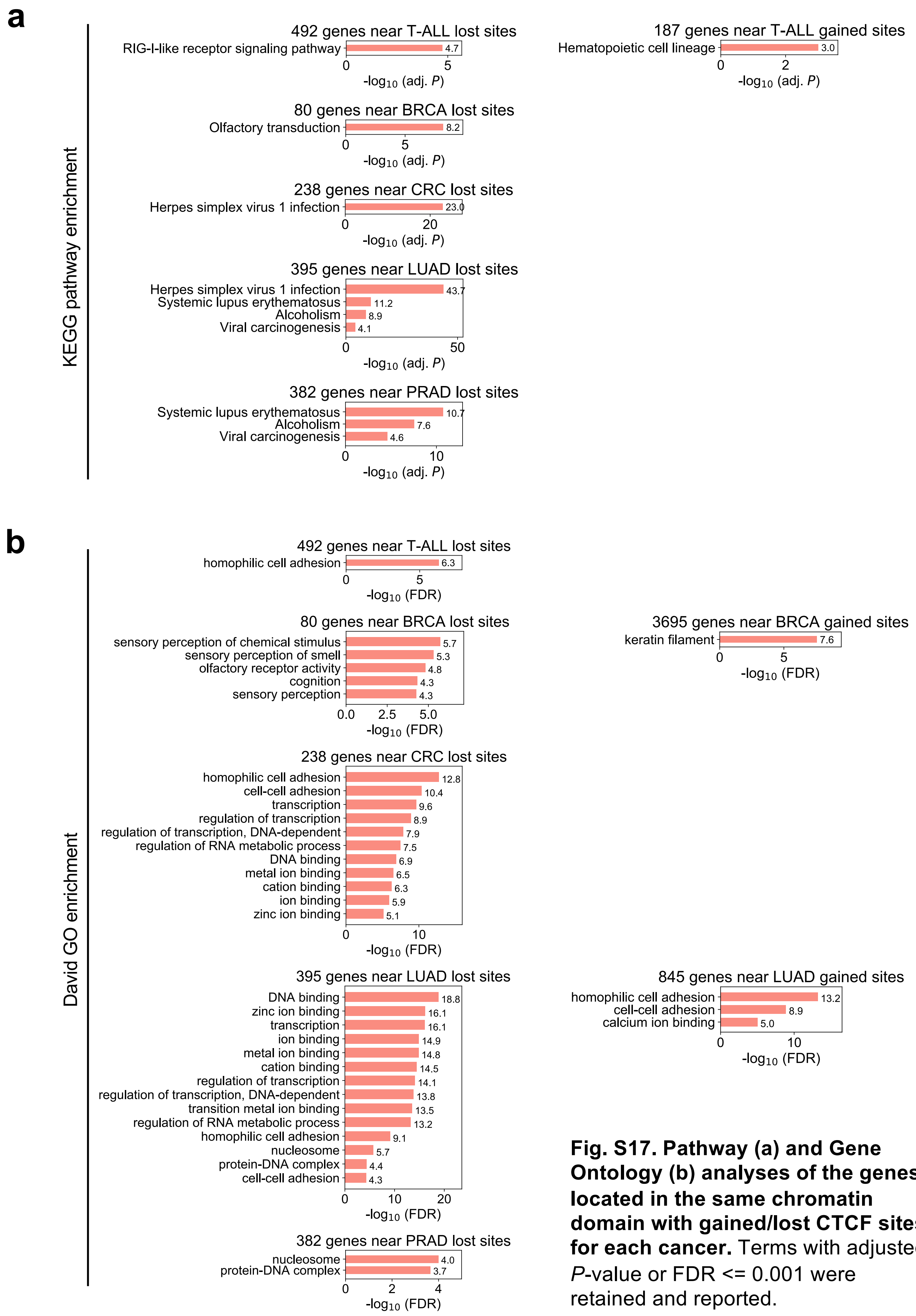

Fig. S17. Pathway (a) and Gene Ontology (b) analyses of the genes located in the same chromatin domain with gained/lost CTCF sites for each cancer. Terms with adjusted *P*-value or FDR <= 0.001 were retained and reported.
