# Supplementary material for: Cancer-specific CTCF binding facilitates oncogenic transcriptional dysregulation
Source: Genome Biol. 2020 Sep 15;21:247. doi: 10.1186/s13059-020-02152-7 (PMC7493976; doi:10.1186/s13059-020-02152-7)
Supplement: Supplementary file 7 — Additional file 7: Table S6. De novo motif analysis results on cancer-specific lost and gained CTCF binding sites in six cancer types, using HOMER and MEME. [file 13059_2020_2152_MOESM7_ESM.pdf]

Table S6. De novo motif analysis results on cancer-specific lost and gained CTCF binding sites in six cancer types, using HOMER and MEME.

HOMER Results

Top 10 motifs with p-value ≤1e-12 were shown for each dataset. N.A., no motifs were reported.

T-ALL lost CTCF binding sites

Rank	Motif	P-value	log P-pvalue	% of Targets	% of Background	STD(Bg STD)	Best Match/Details
1	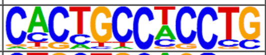	1e-20	-4.623e+01	43.52%	5.62%	177.9bp (157.3bp)	5-TGCCACCT(0.779)<br>More Information | Similar Motifs Found
2	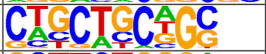	1e-19	-4.554e+01	65.74%	18.88%	166.8bp (203.3bp)	SeqBias: GCW-triplet(0.841)<br>More Information | Similar Motifs Found
3	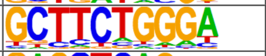	1e-16	-3.704e+01	36.11%	4.70%	197.9bp (180.3bp)	ZNF143|STAF(Zf)/CUTLL-ZNF143-ChIP-Seq(GSE29600)/Homer(0.733)<br>More Information | Similar Motifs Found
4	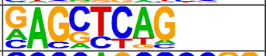	1e-15	-3.474e+01	63.89%	22.70%	175.4bp (208.6bp)	5-CAGAGCTC(0.778)<br>More Information | Similar Motifs Found
5	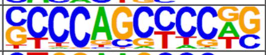	1e-13	-3.092e+01	49.07%	13.79%	155.5bp (202.5bp)	Sp1(Zf)/Promoter/Homer(0.679)<br>More Information | Similar Motifs Found
6	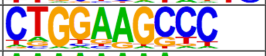	1e-12	-2.986e+01	39.81%	8.72%	125.9bp (208.3bp)	24-GGCTCCCA(0.730)<br>More Information | Similar Motifs Found
7	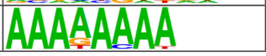	1e-12	-2.911e+01	29.63%	4.14%	215.2bp (175.6bp)	REM19(REM)/colamp-REM19-DAP-Seq(GSE60143)/Homer(0.941)<br>More Information | Similar Motifs Found

T-ALL gained CTCF binding sites

Rank	Motif	P-value	log P-pvalue	% of Targets	% of Background	STD(Bg STD)	Best Match/Details
1	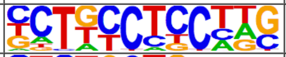	1e-21	-4.946e+01	61.11%	7.11%	92.0bp (94.0bp)	8-TTCCTCCC(0.695)<br>More Information | Similar Motifs Found
2	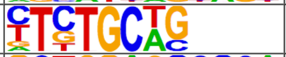	1e-17	-3.917e+01	47.22%	4.62%	109.9bp (142.0bp)	MafA(bZIP)/Islet-MafA-ChIP-Seq(GSE30298)/Homer(0.642)<br>More Information | Similar Motifs Found
3	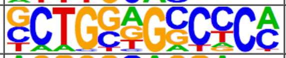	1e-16	-3.773e+01	48.61%	5.51%	74.4bp (88.6bp)	TCP1(TCP)/col-TCP1-DAP-Seq(GSE60143)/Homer(0.654)<br>More Information | Similar Motifs Found
4	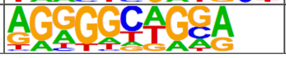	1e-15	-3.456e+01	38.89%	2.75%	122.8bp (72.2bp)	24-GCCCCTGCCCCT(0.691)<br>More Information | Similar Motifs Found

AML lost CTCF binding sites

Rank	Motif	P-value	log P-pvalue	% of Targets	% of Background	STD(Bg STD)	Best Match/Details
1	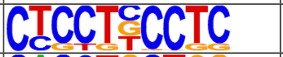	1e-13	-3.053e+01	65.31%	11.59%	160.3bp (218.1bp)	24-CCCCTTCCTC(0.873)<br>More Information | Similar Motifs Found
2	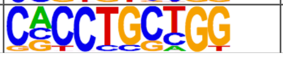	1e-13	-3.052e+01	44.90%	2.64%	104.3bp (184.1bp)	1-CCACYAGRGG(0.853)<br>More Information | Similar Motifs Found

AML gained CTCF binding sites

Rank	Motif	P-value	log P-pvalue	% of Targets	% of Background	STD(Bg STD)	Best Match/Details
1	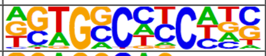	1e-208	-4.811e+02	53.70%	13.63%	87.5bp (134.9bp)	3-TGGCGCCA(0.688)<br>More Information | Similar Motifs Found
2	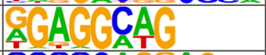	1e-191	-4.418e+02	60.33%	19.61%	95.9bp (143.3bp)	PU.1(ETS)/ThioMac-PU.1-ChIP-Seq(GSE21512)/Homer(0.744)<br>More Information | Similar Motifs Found
3	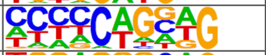	1e-143	-3.303e+02	85.07%	49.74%	99.4bp (137.2bp)	2-CCMCCWGGGGGC(0.777)<br>More Information | Similar Motifs Found
4	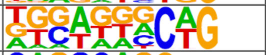	1e-111	-2.561e+02	61.30%	29.10%	107.9bp (155.7bp)	bZIP18(bZIP)/colamp-bZIP18-DAP-Seq(GSE60143)/Homer(0.625)<br>More Information | Similar Motifs Found
5	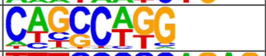	1e-105	-2.433e+02	32.00%	8.28%	108.7bp (117.7bp)	12-CGCCCGGCMG(0.645)<br>More Information | Similar Motifs Found
6	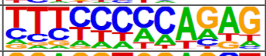	1e-98	-2.276e+02	69.80%	39.03%	102.1bp (134.5bp)	EBF1(EBF)/Near-E2A-ChIP-Seq(GSE21512)/Homer(0.621)<br>More Information | Similar Motifs Found
7	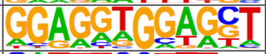	1e-92	-2.120e+02	38.91%	13.86%	104.6bp (131.1bp)	10-AGTTCCCCTTCC(0.711)<br>More Information | Similar Motifs Found
8	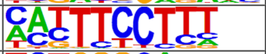	1e-91	-2.109e+02	35.11%	11.40%	101.5bp (136.2bp)	EWS:ERG-fusion(ETS)/CADO_ES1-EWS:ERG-ChIP-Seq(SRA014231)/Homer(0.837)<br>More Information | Similar Motifs Found
9	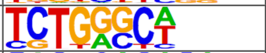	1e-86	-1.986e+02	49.34%	22.37%	104.6bp (141.5bp)	ZNF416(Zf)/HEK293-ZNF416.GFP-ChIP-Seq(GSE58341)/Homer(0.816)<br>More Information | Similar Motifs Found
10	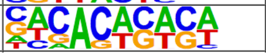	1e-80	-1.849e+02	51.21%	24.75%	105.1bp (124.3bp)	SeqBias: CA-repeat(0.792)<br>More Information | Similar Motifs Found

BRCA lost CTCF binding sites

N.A.

BRCA gained CTCF binding sites

Rank	Motif	P-value	log P-pvalue	% of Targets	% of Background	STD(Bg STD)	Best Match/Details
1	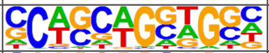	1e-333	-7.689e+02	71.81%	22.12%	149.3bp (231.2bp)	2-CCMCCWGGGGGC(0.827)<br>More Information | Similar Motifs Found
2	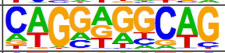	1e-255	-5.890e+02	75.86%	31.49%	140.3bp (195.3bp)	5-TGCCACCT(0.742)<br>More Information | Similar Motifs Found
3	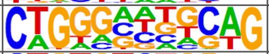	1e-236	-5.450e+02	63.82%	22.26%	160.6bp (194.6bp)	ZNF416(Zf)/HEK293-ZNF416.GFP-ChIP-Seq(GSE58341)/Homer(0.699)<br>More Information | Similar Motifs Found
4	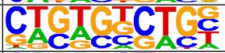	1e-206	-4.753e+02	73.38%	33.40%	144.3bp (189.0bp)	ZNF322(Zf)/HEK293-ZNF322.GFP-ChIP-Seq(GSE58341)/Homer(0.679)<br>More Information | Similar Motifs Found
5	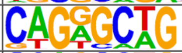	1e-195	-4.500e+02	66.97%	28.35%	157.5bp (192.4bp)	ZNF416(Zf)/HEK293-ZNF416.GFP-ChIP-Seq(GSE58341)/Homer(0.694)<br>More Information | Similar Motifs Found
6	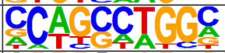	1e-171	-3.945e+02	58.58%	23.35%	164.2bp (177.0bp)	6-GCTCCAGGCCGG(0.687)<br>More Information | Similar Motifs Found
7	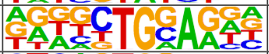	1e-162	-3.734e+02	57.68%	23.45%	157.9bp (175.7bp)	TEAD3(TEA)/HepG2-TEAD3-ChIP-Seq(Encode)/Homer(0.660)<br>More Information | Similar Motifs Found
8	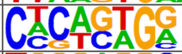	1e-141	-3.269e+02	79.18%	46.59%	149.7bp (192.0bp)	10-CCACAGTG(0.718)<br>More Information | Similar Motifs Found
9	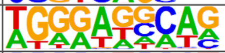	1e-141	-3.262e+02	58.86%	26.43%	156.6bp (188.9bp)	24-GGCTCCCA(0.742)<br>More Information | Similar Motifs Found
10	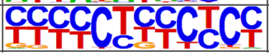	1e-136	-3.139e+02	58.81%	26.94%	181.6bp (197.3bp)	FRS9(ND)/col-FRS9-DAP-Seq(GSE60143)/Homer(0.822)<br>More Information | Similar Motifs Found

CRC lost CTCF binding sites

N.A.

CRC gained CTCF binding sites

Rank	Motif	P-value	log P-pvalue	% of Targets	% of Background	STD(Bg STD)	Best Match/Details
1	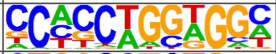	1e-80	-1.853e+02	82.08%	29.52%	98.7bp (204.9bp)	2-CCMCCWGGGGGC(0.933)<br>More Information | Similar Motifs Found
2	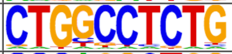	1e-54	-1.253e+02	72.99%	29.54%	109.8bp (177.7bp)	Erra(NR)/HepG2-Erra-ChIP-Seq(GSE31477)/Homer(0.790)<br>More Information | Similar Motifs Found
3	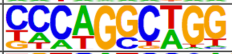	1e-53	-1.222e+02	42.86%	8.30%	116.7bp (214.3bp)	6-GCTCCAGGCCGG(0.707)<br>More Information | Similar Motifs Found
4	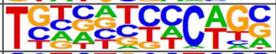	1e-45	-1.056e+02	84.16%	45.27%	128.9bp (180.3bp)	Six2(Homeobox)/NephronProgenitor-Six2-ChIP-Seq(GSE39837)/Homer(0.588)<br>More Information | Similar Motifs Found
5	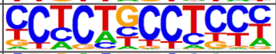	1e-44	-1.015e+02	59.22%	21.60%	137.1bp (192.0bp)	24-CCCCTTCCTC(0.765)<br>More Information | Similar Motifs Found
6	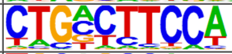	1e-39	-9.006e+01	41.04%	10.67%	103.8bp (161.9bp)	TEAD3(TEA)/HepG2-TEAD3-ChIP-Seq(Encode)/Homer(0.679)<br>More Information | Similar Motifs Found
7	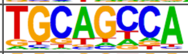	1e-33	-7.823e+01	72.99%	38.65%	124.2bp (176.4bp)	ERF15(AP2EREBP)/colamp-ERF15-DAP-Seq(GSE60143)/Homer(0.699)<br>More Information | Similar Motifs Found
8	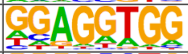	1e-33	-7.744e+01	57.66%	24.58%	110.6bp (169.4bp)	5-TGCCACCT(0.788)<br>More Information | Similar Motifs Found
9	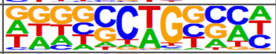	1e-32	-7.533e+01	48.31%	17.68%	102.3bp (154.0bp)	ZNF416(Zf)/HEK293-ZNF416.GFP-ChIP-Seq(GSE58341)/Homer(0.691)<br>More Information | Similar Motifs Found
10	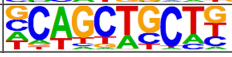	1e-32	-7.452e+01	55.32%	23.21%	147.7bp (162.3bp)	MyoD(bHLH)/Myotube-MyoD-ChIP-Seq(GSE21614)/Homer(0.861)<br>More Information | Similar Motifs Found

LUAD lost CTCF binding sites

Rank	Motif	P-value	log P-pvalue	% of Targets	% of Background	STD(Bg STD)	Best Match/Details
1	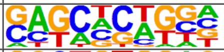	1e-17	-3.941e+01	80.77%	27.20%	251.4bp (311.2bp)	5-CAGAGCTC(0.748)<br>More Information | Similar Motifs Found
2	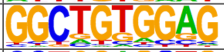	1e-14	-3.344e+01	57.69%	12.87%	203.5bp (265.8bp)	AT3G57600(AP2EREBP)/col-AT3G57600-DAP-Seq(GSE60143)/Homer(0.753)<br>More Information | Similar Motifs Found
3	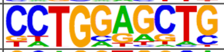	1e-14	-3.344e+01	48.72%	7.95%	240.4bp (601.6bp)	5-ATCTCCAGGA(0.759)<br>More Information | Similar Motifs Found
4	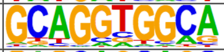	1e-14	-3.340e+01	51.28%	9.26%	198.9bp (277.3bp)	5-TGCCACCT(0.865)<br>More Information | Similar Motifs Found
5	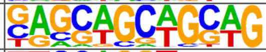	1e-14	-3.284e+01	37.18%	3.06%	319.6bp (113.3bp)	SeqBias: GCW-triplet(0.837)<br>More Information | Similar Motifs Found
6	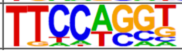	1e-12	-2.916e+01	57.69%	15.38%	223.6bp (184.0bp)	EHF(ETS)/LoVo-EHF-ChIP-Seq(GSE49402)/Homer(0.795)<br>More Information | Similar Motifs Found

LUAD gained CTCF binding sites

Rank	Motif	P-value	log P-pvalue	% of Targets	% of Background	STD(Bg STD)	Best Match/Details
1	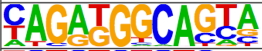	1e-45	-1.038e+02	33.70%	4.91%	93.1bp (174.1bp)	7-TACTGCCCCCTA(0.833)<br>More Information | Similar Motifs Found
2	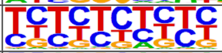	1e-41	-9.647e+01	73.26%	33.73%	150.0bp (196.3bp)	SeqBias: G/A bias(0.876)<br>More Information | Similar Motifs Found
3	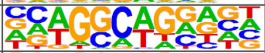	1e-40	-9.418e+01	53.76%	17.66%	134.6bp (223.4bp)	NF1-halfsite(CTF)/LNCaP-NF1-ChIP-Seq(Unpublished)/Homer(0.699)<br>More Information | Similar Motifs Found
4	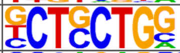	1e-37	-8.585e+01	57.66%	21.99%	154.0bp (182.9bp)	14-CTKCTGGC(0.804)<br>More Information | Similar Motifs Found
5	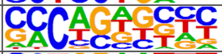	1e-32	-7.537e+01	46.80%	15.75%	158.4bp (186.8bp)	ZNF416(Zf)/HEK293-ZNF416.GFP-ChIP-Seq(GSE58341)/Homer(0.687)<br>More Information | Similar Motifs Found
6	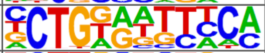	1e-26	-6.080e+01	34.54%	10.10%	121.1bp (183.5bp)	RUNX-AML(Runt)/CD4+-PolII-ChIP-Seq(Barski_et_al.)/Homer(0.628)<br>More Information | Similar Motifs Found
7	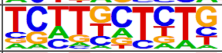	1e-26	-6.041e+01	32.59%	8.99%	116.1bp (150.8bp)	15-CTCTTGCT(0.689)<br>More Information | Similar Motifs Found
8	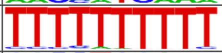	1e-24	-5.651e+01	12.53%	0.60%	116.3bp (164.1bp)	SeqBias: polyA-repeat(0.999)<br>More Information | Similar Motifs Found
9	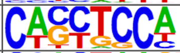	1e-24	-5.559e+01	45.13%	18.25%	143.6bp (184.5bp)	ZNF189(Zf)/HEK293-ZNF189.GFP-ChIP-Seq(GSE58341)/Homer(0.750)<br>More Information | Similar Motifs Found
10	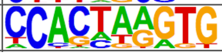	1e-23	-5.362e+01	16.16%	1.87%	106.1bp (147.8bp)	1-CCACYAGRGG(0.776)<br>More Information | Similar Motifs Found

PRAD lost CTCF binding sites

Rank	Motif	P-value	log P-pvalue	% of Targets	% of Background	STD(Bg STD)	Best Match/Details
1	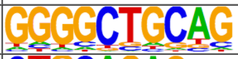	1e-18	-4.232e+01	59.34%	12.81%	196.2bp (217.2bp)	4-GGGCGCTG(0.646)<br>More Information | Similar Motifs Found
2	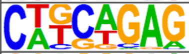	1e-18	-4.210e+01	60.44%	13.58%	209.2bp (170.2bp)	SCL(bHLH)/HPC7-Scl-ChIP-Seq(GSE13511)/Homer(0.681)<br>More Information | Similar Motifs Found
3	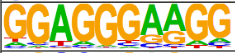	1e-15	-3.673e+01	46.15%	7.78%	166.8bp (243.1bp)	E2F6(E2F)/Hela-E2F6-ChIP-Seq(GSE31477)/Homer(0.737)<br>More Information | Similar Motifs Found
4	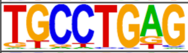	1e-14	-3.406e+01	50.55%	11.10%	192.3bp (256.8bp)	AP-2gamma(AP2)/MCF7-TFAP2C-ChIP-Seq(GSE21234)/Homer(0.690)<br>More Information | Similar Motifs Found
5	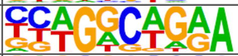	1e-14	-3.401e+01	47.25%	9.13%	213.6bp (275.5bp)	2-CGTTCTACCT(0.681)<br>More Information | Similar Motifs Found
6	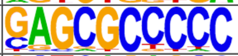	1e-14	-3.375e+01	32.97%	2.96%	160.6bp (500.2bp)	2-AGCGCCCCCT(0.927)<br>More Information | Similar Motifs Found
7	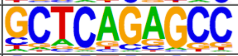	1e-13	-3.204e+01	41.76%	7.10%	172.5bp (246.4bp)	4-GGGCGCTG(0.684)<br>More Information | Similar Motifs Found
8	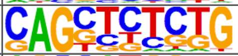	1e-12	-2.988e+01	51.65%	13.53%	191.1bp (165.7bp)	MyoG(bHLH)/C2C12-MyoG-ChIP-Seq(GSE36024)/Homer(0.654)<br>More Information | Similar Motifs Found
9	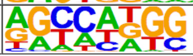	1e-12	-2.819e+01	68.13%	27.27%	214.0bp (176.1bp)	RARg(NR)/ES-RARg-ChIP-Seq(GSE30538)/Homer(0.584)<br>More Information | Similar Motifs Found

PRAD gained CTCF binding sites

Rank	Motif	P-value	log P-pvalue	% of Targets	% of Background	STD(Bg STD)	Best Match/Details
1	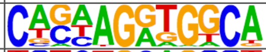	1e-67	-1.562e+02	37.86%	1.93%	47.2bp (86.6bp)	CTCF(Zf)/CD4+-CTCF-ChIP-Seq(Barski_et_al.)/Homer(0.838)<br>More Information | Similar Motifs Found
2	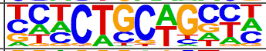	1e-43	-1.010e+02	58.90%	18.00%	107.0bp (146.2bp)	SCL(bHLH)/HPC7-Scl-ChIP-Seq(GSE13511)/Homer(0.760)<br>More Information | Similar Motifs Found
3	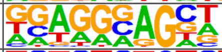	1e-40	-9.310e+01	64.08%	23.38%	100.2bp (143.4bp)	15-GCTCCCTCAC(0.752)<br>More Information | Similar Motifs Found
4	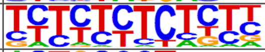	1e-34	-7.934e+01	61.17%	23.78%	111.5bp (123.0bp)	SeqBias: GA-repeat(0.869)<br>More Information | Similar Motifs Found
5	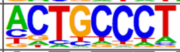	1e-25	-5.774e+01	59.55%	27.48%	97.9bp (145.5bp)	TBP3(MYBrelated)/col-TBP3-DAP-Seq(GSE60143)/Homer(0.793)<br>More Information | Similar Motifs Found
6	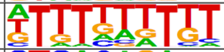	1e-22	-5.187e+01	37.86%	12.34%	97.8bp (129.2bp)	VRN1(ABI3VP1)/col-VRN1-DAP-Seq(GSE60143)/Homer(0.876)<br>More Information | Similar Motifs Found
7	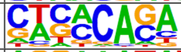	1e-20	-4.799e+01	70.23%	40.47%	110.6bp (150.6bp)	bZIP18(bZIP)/colamp-bZIP18-DAP-Seq(GSE60143)/Homer(0.711)<br>More Information | Similar Motifs Found
8	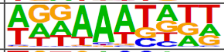	1e-18	-4.298e+01	36.89%	13.52%	106.6bp (108.0bp)	EWS:ERG-fusion(ETS)/CADO_ES1-EWS:ERG-ChIP-Seq(SRA014231)/Homer(0.727)<br>More Information | Similar Motifs Found
9	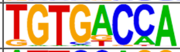	1e-17	-4.113e+01	23.95%	6.00%	94.1bp (106.3bp)	Unknown1(NR/Ini-like)/Drosophila-Promoters/Homer(0.808)<br>More Information | Similar Motifs Found
10	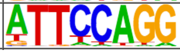	1e-15	-3.510e+01	32.69%	12.48%	100.4bp (128.3bp)	TEAD2(TEA)/Py2T-Tead2-ChIP-Seq(GSE55709)/Homer(0.836)<br>More Information | Similar Motifs Found

MEME Results

Top 10 motifs with p-value ≤1e-2 were shown for each dataset. N.A., no motifs were reported.

T-ALL lost CTCF binding sites

		Logo	E-value	Sites	Width
1.	+ -	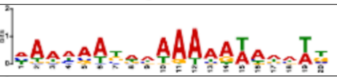	3.6e-030	45	20
2.	+ -	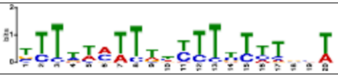	2.1e-023	34	20
3.	+ -	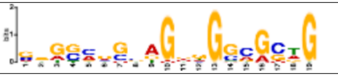	1.7e-011	84	19
4.	+ -	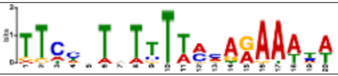	3.0e-008	16	20
5.	+ -	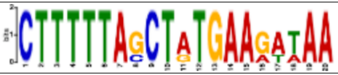	5.2e-003	4	20
6.	+ -	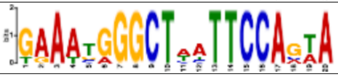	2.2e-003	6	20

T-ALL gained CTCF binding sites

		Logo	E-value	Sites	Width
1.	+ -	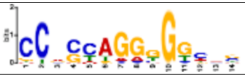	9.0e-019	64	14
2.	+ -	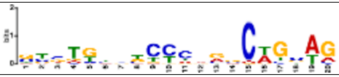	8.8e-003	72	20

AML lost CTCF binding sites

		Logo	E-value	Sites	Width
1.	+ -	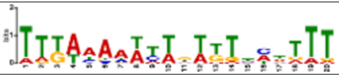	8.7e-011	14	20
2.	+ -	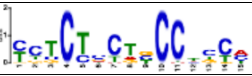	1.3e-005	38	15

AML gained CTCF binding sites

	Logo	E-value	Sites	Width
1.	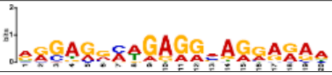	7.4e-066	368	20
2.	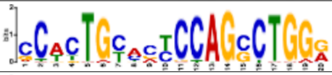	2.4e-078	86	20
3.	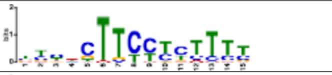	2.6e-062	729	15
4.	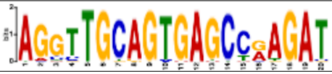	3.7e-061	51	20
5.	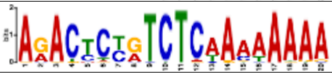	2.8e-048	58	20
6.	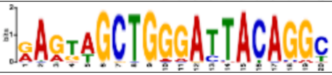	1.7e-050	48	20
7.	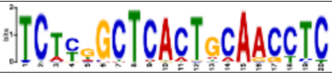	3.4e-048	57	20
8.	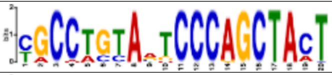	9.0e-047	43	20
9.	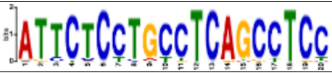	2.2e-033	34	20
10.	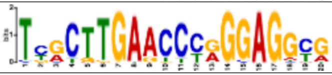	6.5e-023	39	20

BRCA lost CTCF binding sites

N.A.

BRCA gained CTCF binding sites

	Logo	E-value	Sites	Width
1.	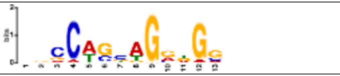	3.4e-064	1773	13
2.	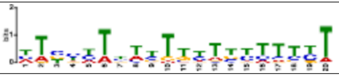	3.2e-010	321	20
3.	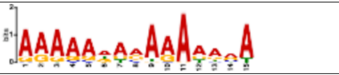	8.7e-018	215	15
4.	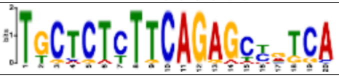	2.4e-008	13	20
5.	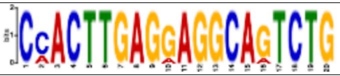	1.0e-005	8	20
6.	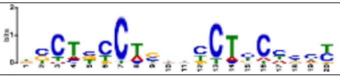	1.7e-005	409	20
7.	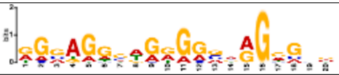	1.3e-005	291	20
8.	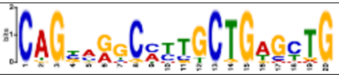	9.1e-003	14	20

CRC lost CTCF binding sites

N.A.

CRC gained CTCF binding sites

	Logo	E-value	Sites	Width
1.	+ - 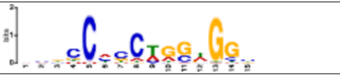	4.6e-067	381	15
2.	+ - 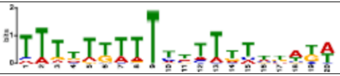	1.2e-040	95	20
3.	+ - 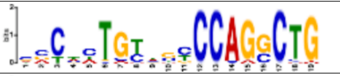	2.5e-029	30	19
4.	+ - 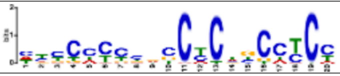	6.4e-027	88	20
5.	+ - 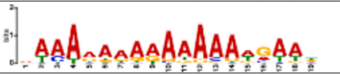	3.8e-020	75	19
6.	+ - 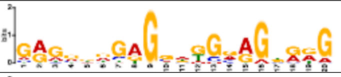	1.9e-031	107	20
7.	+ - 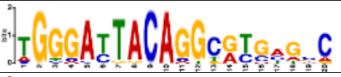	7.1e-012	16	20
8.	+ - 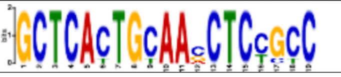	2.9e-009	7	19

LUAD lost CTCF binding sites

	Logo	E-value	Sites	Width
1.	+ - 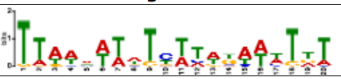	1.1e-011	23	20
2.	+ - 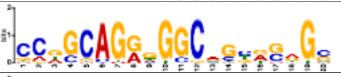	2.4e-011	32	20
3.	+ - 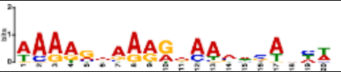	3.3e-008	51	20

LUAD gained CTCF binding sites

	Logo	E-value	Sites	Width
1.	+ - 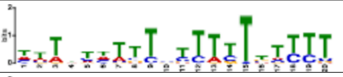	2.5e-040	127	20
2.	+ - 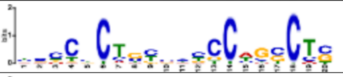	1.6e-024	112	20
3.	+ - 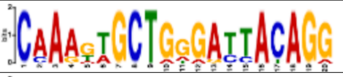	4.8e-021	11	20
4.	+ - 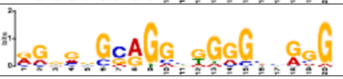	1.3e-015	57	20
5.	+ - 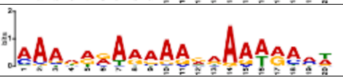	6.1e-015	118	20
6.	+ - 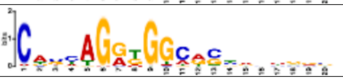	6.2e-013	127	20
7.	+ - 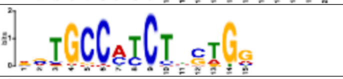	1.2e-011	58	15
8.	+ - 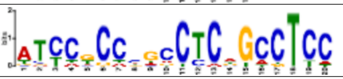	4.4e-012	20	20
9.	+ - 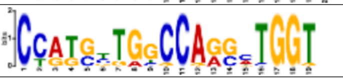	8.9e-009	14	19

PRAD lost CTCF binding sites

	Logo	E-value	Sites	Width
1.	+ - 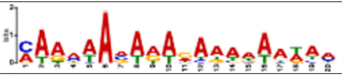	4.6e-021	26	20
2.	+ - 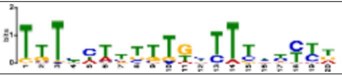	2.8e-012	32	20
3.	+ - 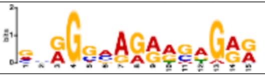	1.1e-007	59	15

PRAD gained CTCF binding sites

	Logo	E-value	Sites	Width
1.	+ - 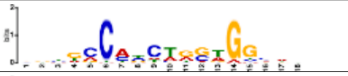	1.3e-104	309	18
2.	+ - 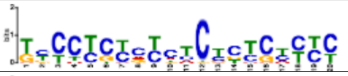	1.7e-016	38	20
3.	+ - 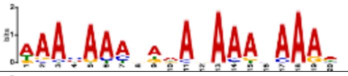	6.0e-014	45	20
4.	+ - 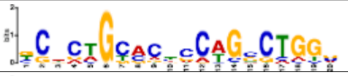	1.8e-011	34	20
5.	+ - 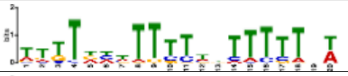	4.0e-010	78	20
6.	+ - 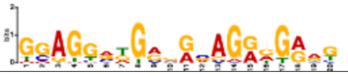	2.8e-005	31	20
