# Supplementary material for: Cancer-specific CTCF binding facilitates oncogenic transcriptional dysregulation
Source: Genome Biol. 2020 Sep 15;21:247. doi: 10.1186/s13059-020-02152-7 (PMC7493976; doi:10.1186/s13059-020-02152-7)
Supplement: Supplementary file 9 — Additional file 9. Review history. [file 13059_2020_2152_MOESM9_ESM.docx]

Review History

**First round of review**

**Reviewer 1**

In this paper, Zang and colleagues used a multi-omic approach to further establish differential CTCF binding as a signature of cancer. They also provide a functional example of CTCF interacting with a transcription factor (NOTCH1) to impact oncogene expression. They analyzed ~800 CTCF ChIP-seq datasets in addition to several RNA-seq, ChIP and HiC datasets to study pan-cancer genome-wide CTCF binding gain and loss events. I was impressed by the comprehensiveness of their multi-omic approach. The study revealed several interesting patterns, including that while the gain and loss of CTCF lead to gain and loss of chromatin interactions as expected (in general), the underlying cause is not DNA methylation or sequence mutation change. However, there are numerous limitations in the individual lines of evidence provided throughout the manuscript, making almost all points they claim a stretch, reducing the rigor of their overall conclusion, and dampening my enthusiasm towards this otherwise strong manuscript. Below I outline some of these limitations.

1.     The overall sensitivity, specificity, and effect size of cancer specific events are lacking. In T-ALL, which the study is largely based on, only 102 lost and 72 gained sites are reported. In Page 6 line 9-11 "Different cancer types share few commonly lost or gained sites, indicating cancer type specificity of the identified CTCF binding patterns". How to rule out that this is due to low sensitivity in detecting common changes? What the commonality among all the peaks? Are the gained / lost sites more (or less) shared compare to all CTCF peaks?

2.     The small number of CTCF alternation reported in T-ALL also casts uncertainty on some of the major genome-wide trend that the authors tried to claim. For example, in Fig5b the authors reported 20% gained CTCF sites are associated with dynamic NOTCH1. That's 15 sites in total. It is hard to generalize this as a genome-wide pattern.

3.     The authors did not show occupancy frequency comparison between cancer type versus corresponding normal tissue (Fig. 1c, Figure S1e-j). Figure S1j: What * and ** mean in the plot?

4.     Page 6 line 45-49 "Specific CTCF binding pattern may also be indicative of clinical outcomes, as patients with elevated chromatin accessibility at gained CTCF binding sites have lower overall survival rate". The conclusion not well supported. Only LUAD (one out of 3 tested) showed a significant p-value.

5.     The authors noted that for a CTCF-gene pair to be considered "highly correlated" it needed a correlation coefficient of 0.25. This seems very low to me, and such a low cut-off warrants an explanation, which is not provided in the paper. I think they should provide a distribution of correlation values to give some context for this cut-off.

6.     The coverage and sequencing depths of RRBS are not sufficient to study the relationship between DNA methylation changes and altered CTCF binding. In Fig. 4a, b, the authors included lots of regions that do not have enough DNA methylation data to support (grey area). If the authors have used only the regions with enough DNA methylation data, association would have been noticed more clearly.

7.     Figure3 d-g what are the promoter ctrl and domain ctrl? Explanations need to be included in the figure legends.

8.     Figure S4: Is there any statistical test to support the statement that cancer-specific gained CTCF binding associates with increased levels of enhancer marks H3K4me1 and H3K27ac?

9.     "analysis of T-ALL patient samples yielded similar results" in figure S5 is just false. There is somewhat reduced gain of CTCF, but there is no visible indication of H3K4me1 or H3K27ac gain.

10.     Page 9 line 29, "The result was confirmed in T-ALL patient samples (FS7)". The conclusion is not supported since almost all the CTCF binding sites are in grey without enough reads. And in general one may reach the conclusion that there's a visible correlation between the change of DNA methylation and the gain / loss of CTCF from figure 4, especially when focusing on regions that have enough WGBS reads. I feel the interpretation offered in page 9 (line 22 to 29), especially the wording "most gained CTCF sites do not associate with DNA methylation reduction" is mis-leading.

11.     For the claim that there is no association between mutation rate and altered CTCF biding, I would like to see more convincing evidence to support that conclusion. Hnisz et al. (2016 Science) used Jurkat cell to show higher mutation rates in boundary CTCF sites. How many of mutated CTCF sites contributed to lost CTCF binding in Jurkat cell? The authors also generated their own WGS data from T-ALL samples. Which samples did they use? Was sequencing depth enough to get mutations?

12.     The authors claim that NOTCH1 is within their top candidates from the BART analysis, however, based on table S5, it ranks around number 200 on the list of associated transcription factors in terms of p-value. I understand they chose NOTCH1 due to its documented association with T-ALL, and they made a convincing case as to its functional interaction with CTCF.  However, I am curious about the function of all of these other transcription factors, that at least statistically have a stronger interaction with CTCF.

13.     I was surprised to see almost no classic HiC patterns (triangular-domains, checkerboard-compartments) in Fig 5e CD4+ T cell. Can the authors comment as to why?

14.     Page 11 line 12-13 "... a portion of those gamma-SI sensitive binding events recovered upon washout". The washout experiment is interesting, but the conclusion is not supported. The loss of CTCF after gamma-SI treatment is visible from the figures, but the recovery is not. Figure S11a shows that after washout, only very limited number of sites gain CTCF and more than half of the sites lose CTCF. There's also no evidence suggesting that the regions gain CTCF after washout are the regions that loss CTCF upon gamma-SI treatment.

15.     Page 11 "BRG1 in the AML cell lines has higher enrichment at AML gained CTCF sites than at constitutive CTCF site,  although CTCF itself has lower binding levels at their respective gained sites in both AML and T-ALL than at constitutive sites, suggesting that gained CTCF binding might need BRG1 to open chromatin". The conclusion is not supported by the observations.

**Reviewer 2**

There exists a tight link between 3D genome architecture, gene regulation, and cancer. While CTCF is known to have a central role in establishing DNA topologies, little is known about how CTCF functions differently in cancer. To address this point, Fang et al. first characterized a comprehensive set of cancer-specific CTCF binding sites from publicly available ChIP-seq data. After discounting several common mechanisms of differential CTCF binding signatures, the researchers uncovered an interesting regulatory relationship between CTCF and the factors NOTCH1 and BRG1. Overall, I found the manuscript to be thorough, mostly well-written, and an important example of how multiple factors can interact to regulate gene expression in cancer that merits further study. However, I have some serious concerns that need to be addressed. Specific comments to follow:

Major

As written, it is unclear to me whether there is a CTCF sequence motif underlying each identified peak. I see Fig 1b, but how does motif presence/absence figure into the rest of the paper and conclusions? Whether there is an underlying CTCF motif is a major concern because it would presumably reflect indirect interactions, and it would be great to know more about which these are (or if this is obvious to the authors, to have it more clearly explained). If cancer-specific CTCF peaks have a clear motif, it would be nice to know why (at the sequence level) this subset of sites are not constitutively bound. Additionally, how do the key results look when you parse things out by binding sites that do vs. don't have an underlying CTCF sequence motif?

On a related point, I would like to see more analyses of how, at the sequence-level, subsets of CTCF sites are distinguished. For example (but this is not necessarily the only place where it could/should be done), it would be great to know if there is anything about the CTCF sites or adjacent sequence context that distinguishes cancer-specific subsets of sites (i.e. both lost & gained subsets). More detailed motif analysis would add value (both of CTCF and any possibly adjacent motifs). As far as I can tell, you are using ChIP-seq data (e.g. for NOTCH) for characterizing subsets of sites. Knowing whether or not specific DNA-level motifs corresponding to particular TFs (or nuances of the CTCF sites) are enriched in these regions feels key for understanding what is really driving these cancer-specific gain/loss subsets.

Additionally, I do not think the manuscript did an adequate job relating the gained or lost CTCF sites to cancer. What are the genes proximal to these sets of cancer-specific CTCF sites? Are they known to have a role in cancer? To this end, gene pathway enrichment analysis would add value to the manuscript.

A second major concern is that throughout the paper, the authors frequently refer to "significantly" or "tend to do this" or "strongly associated" but do not provide fold-effect-sizes nor p-values. It is key that all such references to tests or significance or comparisons include numbers to go along with them. Both fold-changes/effect-sizes and p-values or equivalent are important to note. Frankly it is very difficult for me to judge/review the paper with clarity given that these numbers are almost completely absent.

Minor

The claim of a relationship between CTCF peaks and cancer patient survival is tangential and speculative (on page 6, line 24). Please remove this claim.

The authors refer to peaks with low/high occupancy as cell-type specific/conserved on page 5, line 20. I am under the impression that the ChIP-seq data sets come from not only cell types but also tissues. Therefore, I would suggest changing this to sample-specific/conserved peaks.

**Authors Response**

**Point-by-point responses to the reviewers’ comments:**

In this paper, Zang and colleagues used a multi-omic approach to further establish differential CTCF binding as a signature of cancer. They also provide a functional example of CTCF interacting with a transcription factor (NOTCH1) to impact oncogene expression. They analyzed ~800 CTCF ChIP-seq datasets in addition to several RNA-seq, ChIP and HiC datasets to study pan-cancer genome-wide CTCF binding gain and loss events. I was impressed by the comprehensiveness of their multi-omic approach. The study revealed several interesting patterns, including that while the gain and loss of CTCF lead to gain and loss of chromatin interactions as expected (in general), the underlying cause is not DNA methylation or sequence mutation change. However, there are numerous limitations in the individual lines of evidence provided throughout the manuscript, making almost all points they claim a stretch, reducing the rigor of their overall conclusion, and dampening my enthusiasm towards this otherwise strong manuscript. Below I outline some of these limitations.” Response: We appreciate the reviewer for recognizing the “comprehensiveness” of the work. While accepting certain limitations of this computational study primarily on public data, we have now addressed the reviewer’s comments with more thorough data analyses and have provided additional evidence or clarifications on the points we want to make to improve the rigor of the conclusions.

“1. The overall sensitivity, specificity, and effect size of cancer specific events are lacking. In T-ALL, which the study is largely based on, only 102 lost and 72 gained sites are reported. In Page 6 line 9-11 "Different cancer types share few commonly lost or gained sites, indicating cancer type specificity of the identified CTCF binding patterns". How to rule out that this is due to low sensitivity in detecting common changes? What the commonality among all the peaks? Are the gained / lost sites more (or less) shared compare to all CTCF peaks?”

Response: We used a comprehensive approach to identify cancer-specific lost or gained CTCF sites. This approach includes integration of multiple layers of statistical assessments to make sure that the identified cancer-specific CTCF binding events are true. Briefly, we combined a binding occupancy measure with a quantitative signal comparison measure by integrating over 700 ChIP-seq samples. This approach accounts for both binding specificity across many cell types and differential binding levels in different cell types. Besides what has already been described in the Method section, we perform additional analysis to characterize the sensitivity, specificity and effect size of the identified cancer-specific binding events. First, we examined the differential binding level of the identified CTCF sites compared to all the other cell type samples, and present the results as “half” volcano plots, showing fold change difference as well as significance as quantified by Benjamini-Hochberg adjusted pvalue or FDR (by t-test). The results are presented in Fig. S2a. The lost/gained sites have significantly reduced/elevated binding levels compared to other ChIP-seq samples, with most having an FDR less than 5% and fold change over 2, indicating the accuracy of the identified CTCF sites. Second, we examine the absolute effect sizes of identified lost/gained CTCF sites comparing their normalized ChIP-seq signal level in corresponding cancer samples with all other samples (Fig. S2b). The absolute effect sizes range between 0.93 and 1.87 across different datasets, with an average of 1.46 (Fig. S2c), indicating clear separation between cancer and other samples. We have included these analyses in the revised manuscript.

Based on the current criteria, it is possible that there are certain CTCF binding loss or gain events in a cancer compared to its matched normal cell type that are not identified in our study, because they might not be specific to this cancer. Because of the extensive heterogeneity across cancer types and cell/tissue types, this approach does not aim to detect common patterns in all cancers, but to focus on events that are specific to each individual cancer type.

To answer the reviewer’s latter questions, the commonality of all peaks is described in Fig. 1b using occupancy score measurement. The occupancy measurements described in Fig. 1c and Fig. S1e-o guarantees that cancer-specific lost CTCF sites are among constitutive binding sites (common across most samples) and cancer-specific gained sites do not belong to constitutive sites. When comparing any two cancer types, the cancer-specific gained sites are less shared than all CTCF peaks between the two cancer types, as shown in Fig. S2d & Fig. 1g. The Jaccard Indexes of shared all CTCF peaks are between 0.33 and 0.71, while the Jaccard Indexes of shared gained peaks are at most 0.027 (between BRCA and PRAD) (Table S4).

For cancer-specific lost sites, we assess the significance of shared loss between each pair of cancer types using the Fisher exact tests, using all constitutive CTCF sites as the population pool. Interestingly, although the numbers of shared lost sites are small, we do find that many shared lost sites are significant, with 7 out of the 15 pair-wise comparisons having a p-value < 0.01 (See Table S4). We have revised the text by including these observations in the manuscript.

“2. The small number of CTCF alternation reported in T-ALL also casts uncertainty on some of the major genome-wide trend that the authors tried to claim. For example, in Fig5b the authors reported 20% gained CTCF sites are associated with dynamic NOTCH1. That's 15 sites in total. It is hard to generalize this as a genome-wide pattern.”

Response: In the revised manuscript, we further examine if there is a global association between the gain of CTCF and enrichment of NOTCH1 or dynamic NOTCH1 bindings in T-ALL. We quantify the differential binding level for each CTCF site by applying the Student’s t-test to the normalized binding levels between 8 T-ALL datasets and other datasets. We rank all 285,467 CTCF sites by the differential binding level and evenly categorized them into 100 groups. We find that in a genome-wide scale, those CTCF sites with higher binding levels in T-ALL over other datasets have a higher chance to include a NOTCH1 or dynamic NOTCH1 binding within the same domain regions (Fig. S14e). Although the manuscript focuses on the most confident set of T-ALLgained CTCF sites, the global trend of positive correlation between higher CTCF binding in T-ALL and more NOTCH1 association is clearly shown. On the other hand, 20%-40% of gained CTCF sites are indeed just a portion. We accept that NOTCH1 is only one transcription factor among many factors that can induce CTCF binding gain. As shown by computational prediction in Fig. 5a, there are other factors that potentially have the similar function. We have included this discussion in the revised manuscript.

“3. The authors did not show occupancy frequency comparison between cancer type versus corresponding normal tissue (Fig. 1c, Figure S1e-j). Figure S1j: What * and ** mean in the plot?”

Response: We have added the comparison of occupancy frequency between a cancer type versus its corresponding normal tissue as Fig. S1j-o, as requested. We have also added the missing legends for “*” and “**” signs. *, p<0.05, **, p<0.001, by two-tailed unpaired Student’s t-test. To clarify, as described in details in the Methods section, we use both occupancy frequency and the actual binding levels (measured by ChIP-seq signal) comparing a cancer type with all other samples as well as with its corresponding normal tissue to identify cancer-specific gained/lost sites. The comparison with all other samples can yield a higher specificity than comparison with only the corresponding normal tissue. The comparison of the peak levels between cancer and normal can rule out the cases where the binding site is only tissue-specific but not cancer-specific.

“4. Page 6 line 45-49 "Specific CTCF binding pattern may also be indicative of clinical outcomes, as patients with elevated chromatin accessibility at gained CTCF binding sites have lower overall survival rate". The conclusion not well supported. Only LUAD (one out of 3 tested) showed a significant p-value.”

Response: We agree with this comment and have removed this statement along with the associated Supplementary Figure in the revised manuscript. “5. The authors noted that for a CTCF-gene pair to be considered "highly correlated" it needed a correlation coefficient of 0.25. This seems very low to me, and such a low cut-off warrants an explanation, which is not provided in the paper. I think they should provide a distribution of correlation values to give some context for this cutoff.”

Response: We provided the distribution of correlation values for all CTCF-gene pairs shown as Fig. S8a. To clarify, the cutoff of 0.25 is not for correlation coefficient, but for coefficient of determination, or R-squared, which is equivalent to a correlation coefficient of greater than 0.5 or less than -0.5. We have edited the text to make it more clear. As shown in the figure, the current cutoff yields to only 1.3% (3,567,870 out of 270,382,447) of all CTCF-gene pairs to be considered as highly correlated (blue shaded area), and we believe it is a fair threshold to make rigorous conclusions.

“6. The coverage and sequencing depths of RRBS are not sufficient to study the relationship between DNA methylation changes and altered CTCF binding. In Fig. 4a, b, the authors included lots of regions that do not have enough DNA methylation data to support (grey area). If the authors have used only the regions with enough DNA methylation data, association would have been noticed more clearly.”

Response: We agree that the coverage of RRBS might not be high enough to cover all the altered CTCF binding sites we identified in the genome. Except for LUAD for which we collected WGBS data, we were only able to collect RRBS data from the public domain for the other cancer types. Following the reviewer’s suggestion, we regenerate Figure 4 using only the regions with sufficient DNA methylation data to show the associations, and add a pie chart for each cancer type to show the numbers of lost or gained CTCF sites with sufficient DNA methylation data. Indeed, within the subsets of sites that have adequate RRBS data coverage, most CTCF binding alteration are associated with differential DNA methylation for most cancer types. However, in most cancer types, we still observe some (up to 68% for LUAD) lost CTCF sites without increased DNA methylation and some (22%-85%) gained sites without decrease of DNA methylation. Given that the FDR for lost/gained CTCF sites is 5% and that the threshold for detecting differential methylation is 20%, we are confident that these observations should not be due to lack of data coverage or random noise. Therefore, our conclusion still holds that differential DNA methylation might not be the sole cause that explains all altered CTCF binding events.

“7. Figure3 d-g what are the promoter ctrl and domain ctrl? Explanations need to be included in the figure legends.”

Response: We have revised the figure legends to include a more detailed explanation for the data presented in Figure 3 and Figure S8. “Promoter” refers to genes whose promoter region (TSS +/-2kb) contains a CTCF binding site from a certain category. “Promoter ctrl” refers to genes whose promoter region contains a constitutive CTCF binding site as the control for cancer-specific gained/lost sites. “Intra-domain” refers to genes whose chromatin domain contains a CTCF binding site. “Domain ctrl” refers to genes whose chromatin domain contains a constitutive CTCF site as the control for those with cancer-specific gained/lost sites.

“8. Figure S4: Is there any statistical test to support the statement that cancerspecific gained CTCF binding associates with increased levels of enhancer marks H3K4me1 and H3K27ac?”

Response: Yes, we added the statistical test as suggested. We applied the one-tailed paired Student’s t-test on the normalized histone mark ChIP-seq signals within 2kb regions centered at the CTCF sites between the cancers and the corresponding normal tissues. P-values of the tests were shown in Fig. S6, where * indicates p<0.05, and ** indicates p<0.001. This comparison with significance assessment supports our conclusion that cancer-specific gained CTCF binding associates with increased levels of H3K4me1 and H3K27ac.

“9. "analysis of T-ALL patient samples yielded similar results" in figure S5 is just false. There is somewhat reduced gain of CTCF, but there is no visible indication of H3K4me1 or H3K27ac gain.”

Response: We have reexamined the ChIP-seq data in patient samples and have corrected the corresponding descriptions in the main text. We have updated the figures currently shown as Figure S7, ( *, p<0.05, **, p<0.001, by one-tailed paired Student’s ttest).

“10. Page 9 line 29, "The result was confirmed in T-ALL patient samples (FS7)". The conclusion is not supported since almost all the CTCF binding sites are in grey without enough reads. And in general one may reach the conclusion that there's a visible correlation between the change of DNA methylation and the gain / loss of CTCF from figure 4, especially when focusing on regions that have enough WGBS reads. I feel the interpretation offered in page 9 (line 22 to 29), especially the wording "most gained CTCF sites do not associate with DNA methylation reduction" is mis-leading.”

Response: Consistent with the response to Point 6 above, we have reanalyzed the RRBS data focusing only on the altered CTCF sites that have sufficient bisulfite sequencing read coverage. As shown in current Figure 4, while there are indeed associations between the increase/decrease of DNA methylation and the loss/gain of CTCF binding at most sites, we still observe some lost and gained CTCF sites without changes of DNA methylation. Our conclusion is that differential DNA methylation might not be the sole reason to explain all altered CTCF binding events. The coverage of the RRBS data for the T-ALL patient samples are not high enough to include as many altered CTCF sites as the cell line RRBS data are. Therefore, we have revised the text and have removed the original Figure S7 from the manuscript.

“11. For the claim that there is no association between mutation rate and altered CTCF biding, I would like to see more convincing evidence to support that conclusion. Hnisz et al. (2016 Science) used Jurkat cell to show higher mutation rates in boundary CTCF sites. How many of mutated CTCF sites contributed to lost CTCF binding in Jurkat cell? The authors also generated their own WGS data from T-ALL samples. Which samples did they use? Was sequencing depth enough to get mutations?”

Response: Our WGS data were generated from both Jurkat and CUTLL1 cell lines and two T-ALL patient samples. The average sequencing coverage is ~37x, with majority of the genome between 25x and 50x, sufficient for genetic variation detection (Fig. S10a). In all four samples, we show that there are very few genetic alterations at T-ALL specific CTCF sites (Fig. S10b).

Following the reviewer’s suggestion, we use a similar approach as in the Hnisz et al. 2016 Science paper to examine the mutation rates around CTCF binding sites and present the results as Fig. S12. In fact, Hnisz 2016 Science paper did not use Jurkat cell WGS data. Instead, they used mutation data from all samples combining all cancer types from ICGC database (Fig. 4B-D from Hnisz 2016). While reproducing the mutation rate distribution pattern at constitutive CTCF sites (Fig. S12e), we do not see an enriched mutation rate at the identified cancer-specific gained or lost CTCF sites. We further used mutation data for each cancer type (instead of combining all cancers) from ICGC for AML, BRCA, CRC, LUAD and PRAD and our own WGS data for T-ALL, to examine the mutation rate distribution at gained/lost sites specific to each cancer, and got a similar result (Fig. S12a-d). In conclusion, mutation rates at identified cancerspecific gained/lost CTCF sites are not as high as constitutive bound CTCF sites, suggesting that sequence mutation is not a main contributor to CTCF binding alteration events.

“12. The authors claim that NOTCH1 is within their top candidates from the BART analysis, however, based on table S5, it ranks around number 200 on the list of associated transcription factors in terms of p-value. I understand they chose NOTCH1 due to its documented association with T-ALL, and they made a convincing case as to its functional interaction with CTCF. However, I am curious about the function of all of these other transcription factors, that at least statistically have a stronger interaction with CTCF.”

Response: Table S5 (now Table S6) includes 2 separate data sheets displaying the BART analysis results for T-ALL and colorectal cancer (CRC), respectively. NOTCH1 ranks No. 3 in T-ALL, and ranks No. 260 in CRC, among 883 transcriptional regulators. We wonder whether the reviewer might have seen this from the CRC sheet, as there is no evidence supporting the function of NOTCH1 in CRC. As for T-ALL, we follow the reviewer’s comments and discuss the 2 factors, MYB and RUNX1, that rank higher than NOTCH1. MYB is shown to be a key factor in the initiation of a super-enhancer upstream of the TAL1 gene in a subset of T-ALLs by recruiting CPB and facilitating binding of the TAL1 complex, which contains RUNX1 (Mansour et al., Science, 2014). Meanwhile, a genome wide co-occupancy of MYB and CPB were observed in most super-enhancers present in Jurkat (Mansour et al., Science, 2014).

“13. I was surprised to see almost no classic HiC patterns (triangular-domains, checkerboard-compartments) in Fig 5e CD4+ T cell. Can the authors comment as to why?”

Response: The absence of typical HiC patterns like triangular domains and compartments is due to the scale of this Fig. 5e. At a zoomed-out view on a larger scale, one can see the classic HiC patterns. We also added these analysis results as Suppl. Fig. S15.

“14. Page 11 line 12-13 "... a portion of those gamma-SI sensitive binding events recovered upon washout". The washout experiment is interesting, but the conclusion is not supported. The loss of CTCF after gamma-SI treatment is visible from the figures, but the recovery is not. Figure S11a shows that after washout, only very limited number of sites gain CTCF and more than half of the sites lose CTCF. There's also no evidence suggesting that the regions gain CTCF after washout are the regions that loss CTCF upon gamma-SI treatment.”

Response: We present the data in a slightly different way to clarify the observation that supports the conclusion. We updated Fig. 6a, in which the differential binding level (represented by log2 fold-change) of every CTCF binding site for washout experiment (y-axis) is plotted against the differential binding in GSI treatment (x-axis). The identified T-ALLgained sites are labeled in red. In this plot, it is visible that most TALLgained sites have decreased CTCF binding level after GSI treatment (with negative value on x-axis). Among these decreased CTCF sites, most have an increased CTCF signal after GSI-washout (in the upper-left quadrant). These data support the conclusion that dynamic NOTCH1 binding might induce the dynamic binding change of CTCF.

“15. Page 11 "BRG1 in the AML cell lines has higher enrichment at AML gained CTCF sites than at constitutive CTCF site, although CTCF itself has lower binding levels at their respective gained sites in both AML and T-ALL than at constitutive sites, suggesting that gained CTCF binding might need BRG1 to open chromatin". The conclusion is not supported by the observations.”

Response: We have revised the text to clarify this point. We observed higher BRG1 level at identified gained CTCF sites than constitutive CTCF sites, suggesting that BRG1 might preferentially localize to gained CTCF sites. Future work testing BRG1 function at T-ALLgained sites could provide insights into whether BAF-mediated chromatin remodeling indeed occurs at these gained CTCF sites.

“Reviewer #2: There exists a tight link between 3D genome architecture, gene regulation, and cancer. While CTCF is known to have a central role in establishing DNA topologies, little is known about how CTCF functions differently in cancer. To address this point, Fang et al. first characterized a comprehensive set of cancerspecific CTCF binding sites from publicly available ChIP-seq data. After discounting several common mechanisms of differential CTCF binding signatures, the researchers uncovered an interesting regulatory relationship between CTCF and the factors NOTCH1 and BRG1. Overall, I found the manuscript to be thorough, mostly wellwritten, and an important example of how multiple factors can interact to regulate gene expression in cancer that merits further study. However, I have some serious concerns that need to be addressed. Specific comments to follow:”

Response: We thank the reviewer for the thorough review and overall positive comments to the manuscript. We address the concerns point-by-point below.

“Major As written, it is unclear to me whether there is a CTCF sequence motif underlying each identified peak. I see Fig 1b, but how does motif presence/absence figure into the rest of the paper and conclusions? Whether there is an underlying CTCF motif is a major concern because it would presumably reflect indirect interactions, and it would be great to know more about which these are (or if this is obvious to the authors, to have it more clearly explained). If cancer-specific CTCF peaks have a clear motif, it would be nice to know why (at the sequence level) this subset of sites are not constitutively bound. Additionally, how do the key results look when you parse things out by binding sites that do vs. don't have an underlying CTCF sequence motif?”

Response: In general, all observations on identified gained/lost CTCF sites in this study does not distinguish presence or absence of the CTCF motif. We have added several supplementary figures (Figures S3, S5, S9) to show that the key results are consistent if we look at CTCF sites with or without motifs separately. Although some results are no longer deemed significant based on the P-values, mainly because the data size are smaller after being separated, we do see that the trends are in the same direction. Overall, we did not discriminate CTCF binding sites based on the motif occurrence, based on a fundamental assumption that ChIP-seq data directly provide information about TF-DNA interactions, regardless of motif occurrence. We do not exclude the possibilities that many CTCF binding events may be through indirect interactions, but the function of these CTCF binding sites in inducing chromatin interactions and facilitating gene regulation is by and large similar, according our analyses. In addition, it is worth noting that motif occurrence alone is not enough for TF binding. Under a uniform threshold (scanned using FIMO for CTCF motif PWM from Jasper database under the default P-value cutoff of 1e-4), we identified 877,981 CTCF motif hits across the whole human genome. Among these hits, 639,704 (72%) are located outside any of the 688,429 union CTCF binding sites curated from ChIP-seq data. It is not surprising to identify new CTCF binding sites from a new cell type, with or without a motif. We have included these clarifications and explanations in the revised manuscript in Results and Discussions sections.

“On a related point, I would like to see more analyses of how, at the sequence-level, subsets of CTCF sites are distinguished. For example (but this is not necessarily the only place where it could/should be done), it would be great to know if there is anything about the CTCF sites or adjacent sequence context that distinguishes cancer-specific subsets of sites (i.e. both lost & gained subsets). More detailed motif analysis would add value (both of CTCF and any possibly adjacent motifs). As far as I can tell, you are using ChIP-seq data (e.g. for NOTCH) for characterizing subsets of sites. Knowing whether or not specific DNA-level motifs corresponding to particular TFs (or nuances of the CTCF sites) are enriched in these regions feels key for understanding what is really driving these cancer-specific gain/loss subsets.”

Response: We did not present motif analysis results not because we did not do it, but because we did not find any positive results from sequence motif analysis. We actually started with sequence motif analysis when characterizing the identified gained/lost CTCF sites in cancer. However, we did not find any motif, other than CTCF itself, enriched in a 600bp region surrounding the T-ALLgained or T-ALLlost CTCF sites (new added Fig. S13). We could not find interesting motif enriched in the gained/lost CTCF peak regions for most other cancer types either. That is exactly the reason why we sought to scan further regions beyond the identified CTCF binding sites, namely, genomic regions that show differential chromatin interactions with these CTCF binding sites. Hi-C determined chromatin interaction regions have a resolution of 5Kb, making sequence motif analysis difficult or lack of specificity, so we used our novel method, BART, for TF identification based on publicly available ChIP-seq data. We showed that the BART approach indeed worked in identifying NOTCH1 among other factors from TALL. The data presented in this work actually demonstrate that ChIP-seq based approaches can be more efficient when sequence motif approaches do not work.

“Additionally, I do not think the manuscript did an adequate job relating the gained or lost CTCF sites to cancer. What are the genes proximal to these sets of cancer-specific CTCF sites? Are they known to have a role in cancer? To this end, gene pathway enrichment analysis would add value to the manuscript.”

Response: Following the reviewer’s suggestion, we perform pathway enrichment and gene ontology analyses for the genes located in the same chromatin domains with the gained/lost CTCF sites specific to each cancer. The results are shown as Fig. S17.

“A second major concern is that throughout the paper, the authors frequently refer to "significantly" or "tend to do this" or "strongly associated" but do not provide fold-effectsizes nor p-values. It is key that all such references to tests or significance or comparisons include numbers to go along with them. Both fold-changes/effect-sizes and p-values or equivalent are important to note. Frankly it is very difficult for me to judge/review the paper with clarity given that these numbers are almost completely absent.”

Response: We agree with the comment that statistical significance measures are extremely important, and have added the missing statistical assessments including pvalues and/or fold-change values for all comparisons throughout the manuscript.

“Minor

The claim of a relationship between CTCF peaks and cancer patient survival is tangential and speculative (on page 6, line 24). Please remove this claim.”

Response: We agree with this comment and have removed this claim from the manuscript.

“The authors refer to peaks with low/high occupancy as cell-type specific/conserved on page 5, line 20. I am under the impression that the ChIP-seq data sets come from not only cell types but also tissues. Therefore, I would suggest changing this to samplespecific/ conserved peaks.”

Response: We have revised the manuscript following the reviewer’s suggestion.

**Second round of review**

**Reviewer 1**

I thank the authors for addressing many of my questions. The authors did an excellent job revising the paper. The manuscript is indeed much improved, in particular in its rigor and clarity.

Given the broad scale of the analysis included in the manuscript, I would encourage the authors to exercise stronger caution in stating the facts and in drawing conclusions. This is mainly a comment on their writing.

For example, the authors continue to emphasize that lost and gain of CTCF bindings are not always associated with DNA methylation. I understand that the authors wish to claim “unexpected findings”, thus have taken such a tune in describing the results. However, the more carefully stated conclusion, “differential DNA methylation might not be the sole cause that explains all altered CTCF binding events”, is a useless one. In genomics one can always find numerous exceptions to even the most established rules. This conclusion does not advance the field. For a paper that is almost entirely built on correlation analyses, the correlation between methylation and CTCF binding (figure 4, which is a beautiful figure with strong results) is probably among the strongest, yet here the authors chose to essentially say the correlation is not perfect. Moreover, this is based on lack of observing more than 20% of DNA methylation, thus the evidence is fairly weak. There is really no need to sensationalize the results, which mostly fit expectations (at least in the context of CTCF and methylation change). Readers are probably more interested in knowing whether gained CTCF sites are unmethylated to begin with, if there is no strong evidence of reduction of DNA methylation. The authors have to further characterize the differences between sites with DNA methylation change and sites without DNA methylation change, if they wish to make a rigorous conclusion.

There are a few such places where the authors might want to write with similar caution, e.g., cancer type specific pattern (what explains the large differences across the cancer types, revealed by new figure S2? How does number of datasets influence rate of discovery?), genome-wide association between CTCF and Notch1 (what is your positive control and negative control?), association with histone marks (what’s going on with H3K27me3?), etc. Again, given the already very large scale of the analysis in this paper, I don’t expect the authors to take every point home. However, the writing should reflect facts, and care should be taken to avoid stretching conclusions and potentially misleading readers.

**Reviewer 2**

The authors have reasonably addressed my concerns.

Regarding the response to my comment about sequence motif analysis. I recognize that the motif enrichment was fruitless, but was de novo motif analysis performed? If not, then it would be interesting to see if there are any additional new motif hits and include the results in the relevant supplementary figure.

**Authors Response**

**Point-by-point responses to the reviewers’ comments:**

We thank the reviewer for acknowledging the improvement of the revised manuscript. We agree with the reviewer about using caution when drawing conclusions, and have further revised the manuscript by adding some clarifications with supporting evidence. Specifically,

1) Regarding association between CTCF binding loss/gain and DNA methylation: We understand the reviewer’s concern, and have further revised the manuscript for clarification accordingly. We did observe that loss and gain of CTCF binding partially associate with DNA methylation changes, both at the identified lost/gained sites (Fig. 4a,b) and across all CTCF sites (newly added Fig. 4c). While others (e.g., Flavahan et al. Nature 2015, Nature 2019) established a causal relationship between DNA methylation change and CTCF binding alteration in several cancer types, our work showed that oncogenic TF binding such as NOTCH1 in T-ALL can be an alternative cause of aberrant CTCF binding events observed in cancer. Existing data from the public domain and our validation experiments do not provide sufficient evidence to determine the association between oncogenic TF induced CTCF binding events and DNA methylation reduction. This can be an interesting question for further studies. Indeed, we do not intend to argue the established genomic correlation between DNA methylation and CTCF binding nor to overstate some exceptions, but want to interpret our findings in an objective and rigorous manner.

2) Regarding large differences across cancer types influenced by the number of datasets: We have added a statement in the corresponding paragraph in the Results section: “It is worth noting that the large difference across cancer types on the number of lost/gained CTCF sites identified under the same statistical criteria is possibly due to the various number of available samples for different cancer types (Fig. S1e-o) and the wide range of CTCF peak numbers across samples (Fig. S1a).” and in the Discussion section: “Various numbers of available datasets might cause the large difference on the numbers of identified lost/gained CTCF sites across cancer types.”

3) Regarding control for genome-wide association between CTCF and Notch1 in TALL: We have revised Figure S14e by adding a background control. The data shown in Fig. S14e indicates that if a CTCF site is more specific in T- ALL over other samples, there is a higher chance to find a NOTCH1 or dynamic NOTCH1 binding within the same chromatin domain. A background control of this association can be the chance of observing NOTCH1 or dynamic NOTCH1 binding in the same domain of any CTCF site in T-ALL. By randomly sampled from all CTCF sites in T-ALL, the number of sites associated with NOTCH1 in a group of CTCF sites follows a hypergeometric distribution, for which the mean level and 95% confidence interval have now been plotted in the revised Fig. S14e. With this control, one can see that the T-ALL-specific CTCF sites do have a significant higher chance to be associated with NOTCH1 and dynamic NOTCH1.

4) Regarding H3K27me3 patterns surrounding lost/gained CTCF sites: We have added a statement in the corresponding paragraph in the Results section: “We did not observe a consistent trend of change in H3K27me3 level at either gained or lost CTCF sites.”

Response to Reviewer #2:

Following the reviewer’s suggestion, we have included the de novo motif analysis results from two different methods, HOMER and MEME, in Supplementary Table S6. We did not find any new motifs with unambiguous enrichment. Nevertheless, we include the data in Supplementary Materials as a resource for readers.

**Third round of review**

**Reviewer 1**

I have no further comments.

**Reviewer 2**

All my comments have been addressed
